# Supplementary material for: Antibacterial and Antioxidant Activity of Synthetic Polyoxygenated Flavonoids
Source: Int J Mol Sci. 2024 May 30;25(11):5999. doi: 10.3390/ijms25115999 (PMC11172986; doi:10.3390/ijms25115999)
Supplement: Supplementary file 1 [file ijms-25-05999-s001.zip › ijms-3022711-supplementary.pdf]

Antibacterial and antioxidant activity of synthetic polyoxygenated flavonoids

Mauricio Osorio-Olivares [1,*], Yesseny Vásquez-Martínez [2], Katy Díaz [3], Lautaro Taborga [4], Luis Espinoza [1].

Representative images of the bacteria used in this study

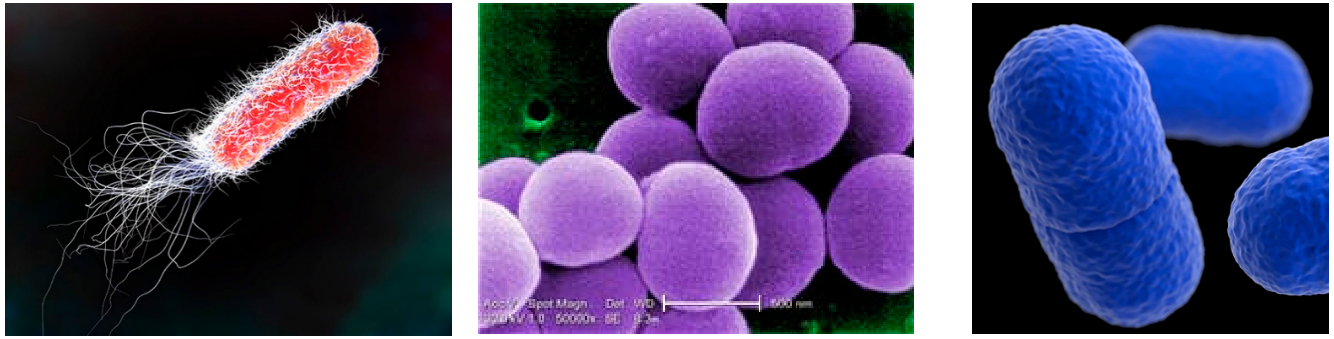

A B

C

Figure S1. Representative images of the bacteria used in this study. *Escherichia coli* (A)[36], *Staphylococcus aureus* (B)[37], *Listeria monocytogenes* (C)[38].

Physical Data of all synthesized compounds

Flavanones (**FV1 – FV11**).

2-(4-hydroxy-3-methoxyphenyl)-5,7-dimethoxychroman-4-one (**FV1**) was obtained as racemic mixtures because these was synthesized from chalcone (**C1**) (1.36 mmol), NaOAc (9.52 mmol) and EtOH/H_2O, then under heated to reflux as described above. This crude was then hydrolyzed with a mixture of aqueous HCl (1 N)/ethanol/isopropanol at reflux and then purified by column chromatography using ethyl acetate-hexane in a isocratic system (80 % ethyl acetate in hexane) as the mobile phase to afford **FV1** as a pale yellow powder (277 mg, 62% from chalcone **C1**); mp: 144.3 — 145.6 °C (lit. [39] 145 — 146 °C); IR (film): ν_{max} cm^{-1}: 3338, 2939, 1667, 1607, 1571, 1519, 1457, 1424, 1433, 1355, 1268, 1216, 1158, 1110, 1071, 1035, 820. ^{1}H-NMR (DMSO-d_6) δ ppm: 9.13 (s, 1H, Ar-O*H*-4'); 7.07 (d, 1H, *J* = 1.3 Hz, Ar*H*-2'); 6.89 (dd, 1H, *J* = 8.1 and 1.4 Hz, Ar*H*-6'); 6.77 (d, 1H, *J* = 8.0 Hz, Ar*H*-5'); 6.19 (m, 2H, Ar*H*-6,8); 5.36 (dd, 1H, *J*=12.9 and 2.6 Hz, C*H*-2); 3.78 — 3.76 (m, 9H, CH_3O-5,7,3'); 3.09 (dd, 1H, J_1 = 16.4 and 13.0 Hz, CH*H*-3); 2.54 (dd, 1H, *J* = 16.4 and 2.8 Hz, C*H*H-3). ^{13}C-NMR (DMSO-d_6) δ ppm: 188.2 (4-*C*=O); 165.4 (Ar*C*-7); 164.5 (Ar*C*-5); 161.8 (Ar*C*-1a); 147.5 (Ar*C*-3');

146.8 (Ar*C*-4′); 129.7 (Ar*C*-1′); 119.5 (Ar*C*-6′); 115.2 (Ar*C*-5′); 111.1 (Ar*C*-2′); 105.4 (Ar*C*-4a); 93.7 (Ar*C*-6); 92.8 (Ar*C*-8); 78.6 (*C*H-2); 55.8 (*C*H_3O-3′); 55.7 (*C*H_3O-5,7); 44.8 (*C*H_2-3).

2-(3,4-dihydroxyphenyl)-5,7-dihydroxychroman-4-one (**FV2**; (±)-Eriodictyol) was obtained as racemic mixtures because these was synthesized from chalcone (**C2**) (1.1 mmol), NaOAc (7.7 mmol) and EtOH/H_2O, then under heated to reflux as described above. This crude was then hydrolyzed with a mixture of aqueous HCl (1 N)/ethanol/isopropanol at reflux and then purified by column chromatography using ethyl acetate-hexane in a isocratic system (50 % ethyl acetate in hexane) as the mobile phase to afford **FV2** as a white solid (218.7 mg, 69% from chalcone **C2**); mp: 267 — 269°C dec. (lit. [40] 282 — 284 °C). IR (KBr): ν max cm^{-1}: 3363, 3143, 1636, 1605, 1534, 1476, 1451, 1393, 1344, 1311, 1258, 1219, 1187, 1158, 1119, 1085, 1068, 1013, 825, 734, 552. ^{1}H-NMR (DMSO-*d*$_6$) δ ppm: 12.13 (s, 1H, Ar-O*H*-5); 10.75 (s, 1H, Ar-O*H*-7); 9.03 (s, 2H, Ar-O*H*-3′,4′); 6.86 (s, 1H, Ar*H*-6′); 6.73 (s, 2H, Ar*H*-2′,5′); 5.86 (s, 2H, Ar*H*-6,8); 5.36 (dd, 1H, *J*=12.4 and 2.4 Hz, C*H*-2); 3.17 (dd, 1H, , *J*=17.1 and Hz, CH*H*-3); 2.66 (dd, 1H, *J* = 17.1 and 2.6 Hz, C*H*H-3. ^{13}C-NMR (DMSO-*d*$_6$) δ ppm: 196.3 (4-*C*=O); 166.7 (Ar*C*-7); 163.5 (Ar*C*-5); 162.9 (Ar*C*-8a); 145.7 (Ar*C*-4′); 145.2 (Ar*C*-3′); 129.4 (Ar*C*-1′); 117.9 (Ar*C*-6′); 115.3 (Ar*C*-5′); 114.3 (Ar*C*-2′); 101.8 (Ar*C*-4a); 95.7 (Ar*C*-6); 94.9 (Ar*C*-8); 78.4 (*C*H-2); 42.0 (*C*H_2-3).

5,7-dihydroxy-2-(4-hydroxy-3-methoxyphenyl)chroman-4-one (**FV3;** ±-Homoeriodictyol) was obtained as racemic mixtures because these was synthesized from chalcone (**C3**) (0.92 mmol), NaOAc (6.44 mmol) and EtOH/H_2O, then under heated to reflux as described above. This crude was then hydrolyzed with a mixture of aqueous HCl (1 N)/ethanol/isopropanol at reflux and then purified by column chromatography using ethyl acetate-hexane in a isocratic system (50 % ethyl acetate in hexane) as the mobile phase to afford **FV3** as a white solid (127.9 mg, 46% from chalcone **C3**); mp: 225.6 — 227.3 °C (lit. [41] 228 — 230 °C). IR (KBr): $ν_{max}$ cm^{-1}: 3464, 3129, 2937, 2640, 1636, 1614, 1583, 1524, 1498, 1434, 1352, 1335, 1310, 1294, 1275, 1260, 1192, 1163, 1088, 1069, 1031, 1017, 878, 845, 814, 781, 755, 744, 679, 656, 637, 562, 546. ^{1}H-NMR (DMSO-*d*$_6$) δ ppm: 12.15 (s, 1H, Ar-O*H*-5); 10.76 (s, 1H, Ar-O*H*-7);); 9.16 (s, 1H, Ar-O*H*-4′); 7.08 (s, 1H, Ar*H*-2′); 6.89 (d, 1H, *J* = 8.2 Hz ,Ar*H*-6′); 6.78 (d, 1H, *J* = 8.2 Hz, Ar*H*-5′); 5.87 (d, 2H, *J* = 2.8 Hz, Ar*H*-6,8); 5.41 (dd, 1H, *J*=12.8 and 2.5 Hz, C*H*-2); 3.77 (s, 3H, C*H*$_3$O-3′); 3.31 (dd, 1H, *J*=17.1 and 12.9 Hz; CH*H*-3); 2.67 (dd. 1H, *J*=17.1 and 2.7 Hz, C*H*H-3). ^{13}C-NMR (DMSO-*d*$_6$) δ ppm: 196.4 (4-*C*=O); 166.7 (Ar*C*-7); 163.5 (Ar*C*-5); 162.9 (Ar*C*-8a); 147.5 (Ar*C*-3′); 147.0 (Ar*C*-4′); 129.4 (Ar*C*-1′); 119.7 (Ar*C*-2′); 115.2 (Ar*C*-5′); 111.2 (Ar*C*-6′); 101.7 (Ar*C*-4a); 95.8 (Ar*C*-6); 95.0 (Ar*C*-8); 78.7 (*C*H-2); 55.7 (*C*H_3O-3′); 42.1 (*C*H_2-3).

5,7-dihydroxy-2-(2,4,6-trimethoxyphenyl)chroman-4-one (**FV4**) was obtained as racemic mixtures because these was synthesized from chalcone (**C4**) (1.61 mmol), NaOAc (11.3 mmol) and EtOH/H_2O, then under heated to reflux as described above. This crude was then hydrolyzed with a mixture of aqueous HCl (1 N)/ethanol/isopropanol at reflux and then purified by column chromatography using ethyl acetate-hexane in isocratic system (50 % ethyl acetate in hexane) as the mobile phase to afford **FV4** as a white solid (78.1 mg, 14% from chalcone **C4**); mp: 263.5 – 266.7 °C. IR (KBr): $ν_{max}$ cm^{-1}: 3461, 3134, 2940, 2846, 1627, 1601, 1500, 1461, 1391, 1353, 1299, 1229, 1206, 1293, 1179, 1164, 1129, 1090, 1066, 1037, 803, 753, 733. ^{1}H-NMR (DMSO-*d*$_6$) δ ppm: 12.24 (s, 1H, Ar-O*H*-5); 10.75 (br. s, 1H, Ar-O*H*-7);); 6.27 (s, 2H, Ar*H*-3′,5′); 5.84 — 5.80 (m, 3H, Ar*H*-6,8, C*H*-2); 3.80 (s, 3H, C*H*$_3$O-4′); 3.76 (s, 6H, C*H*$_3$O-2′,6′); 2.42 (dd, 1H, *J* = 17.5 and 3.0 Hz, CH*H*-3). ^{13}C-NMR (DMSO-*d*$_6$) δ ppm: 197.3 (4-*C*=O); 166.4 (Ar*C*-7); 163.8 (Ar*C*-5); 163.7 (Ar*C*-

8a); 161.8 (Ar*C*-4′); 159.7 (Ar*C*-2′,6′); 105.4 (Ar*C*-1′); 101.5 (Ar*C*-4a); 95.5 (Ar*C*-6); 94.8 (Ar*C*-8); 91.3 (Ar*C*-3′,5′); 70.8 (C*H*-2); 55.9 (CH_3O-2′,6′); 55.4 (CH_3O-4′); 39.3 (CH_2-3).

5,7-dihydroxy-2-(4-hydroxyphenyl)chroman-4-one (**FV5**; ±-Naringenin) was obtained as racemic mixtures because these was synthesized from chalcone (**C5**) (1.39 mmol), NaOAc (9.73 mmol) and EtOH/H_2O, then under heated to reflux as described above. This crude was then hydrolyzed with a mixture of aqueous HCl (1 N)/ethanol/isopropanol at reflux and then purified by column chromatography using methanol in methylene chloride in a isocratic system (90 % methylene chloride acetate / 10 % methanol) as the mobile phase to afford **FV5** as a white solid (211.9 mg, 56% from chalcone **C5**); mp: 253.4 – 255.0 °C (lit. [42] 252 – 253 °C. IR (KBr): ν_{max} cm^{-1}: 3287, 3114, 3036, 2920, 2831, 2699, 1629, 1601, 1519, 1498, 1462, 1422, 1389, 1337, 1312, 1249, 1181, 1157, 1083, 1064, 1014, 970, 890, 842, 832, 760, 730, 667, 564, 532. ^{1}H-NMR (DMSO-*d*$_6$) δ ppm: 12.14 (s, 1H, Ar-O*H*-5); 10.78 (s, 1H, Ar-O*H*-7); 9.58 (s, 1H, Ar-O*H*-4′); 7.30 (d, 2H, *J*=8.4 Hz, Ar*H*-2′,6′); 6.78 (d, 2H, *J*=8.4 Hz, Ar*H*-3′,5′); 5.87 (s, 2H, Ar*H*-6,8); 5.43 (dd, 1H, *J*=12.8 and 2.5 Hz, C*H*-2); 3.26 (dd, 1H, , *J*=17.1 and 12.9 Hz, CH*H*-3); 2.67 (dd, 1H, *J* = 17.1 and 2.8 Hz, C*H*H-3). ^{13}C-NMR (DMSO-*d*$_6$) δ ppm: 196.4 (4-*C*=O); 166.6 (Ar*C*-7); 163.4 (Ar*C*-5); 162.9 (Ar*C*-8a); 157.7 (Ar*C*-4′); 128.8 (Ar*C*-1′); 128.3 (Ar*C*-2′,6′); 115.1 (Ar*C*-3′,5′); 101.8 (Ar*C*-4a); 95.8 (Ar*C*-6); 95.0 (Ar*C*-8); 78.4 (CH-2); 42.0 (CH_2-3).

5,7-dihydroxy-2-(4-(methylthio)phenyl)chroman-4-one (**FV6**) was obtained as racemic mixtures because these was synthesized from chalcone (**C6**) (5.1 mmol), NaOAc (35.7 mmol) and EtOH/H_2O, then under heated to reflux as described above. This crude was then hydrolyzed with a mixture of aqueous HCl (1 N)/ethanol/isopropanol at reflux and then purified by column chromatography using ethyl acetate in hexane in a gradient system (0 — 50 % ethyl acetate in hexane) as the mobile phase to afford **FV6** as a white powder (591.7 mg, 51% from chalcone **C6**); mp: 236.8 — 238.0 °C; HRMS *m*/*z*, observed: 303.0691; $C_{16}H_{14}O_4S$ [M + H]$^+$ requires: 303.0686. IR (film): ν_{max} cm^{-1}: 3166, 2919, 2840, 1635, 1600, 1493, 1434, 1341, 1311, 1299, 1176, 1161. ^{1}H-NMR (DMSO-*d*$_6$) δ ppm: 12.11 (s, 1H, ArO*H*-5); 10.82 (s, 1H, ArO*H*-7); 7.44 (d, 2H, *J* = 8.2 Hz, Ar*H*-3′,5′); 7.30 (d, 2H, *J* = 8.2 Hz, Ar*H*-2′,6′); 5.91 (d, 1H, *J* = 1.6 Hz, Ar*H*-8); 5.89 (s, 1H, Ar*H*-6); 5.54 (dd, 1H, *J* = 12.4 and 2.6 Hz, C*H*-2); 3.24 (dd, 1H, *J* = 17.1 and 12.5 Hz, C*H*H-3); 2.75 (dd, 1H, *J* = 17.1 and 3.0 Hz, CH*H*-3); 2.48 (s, 3H, CH_3S-4′). ^{13}C-NMR (DMSO-*d*$_6$) δ ppm: 196.0 (4-*C*=O); 166.7 (Ar*C*-7); 163.5 (Ar*C*-5); 162.7 (Ar*C*-8a); 138.7 (Ar*C*-4′); 135.1 (Ar*C*-1′); 127.3 (Ar*C*-3′,5′); 125.8 (Ar*C*-2′,6′); 101.8 (Ar*C*-4a); 95.9 (Ar*C*-6); 95.0 (Ar*C*-8); 78.0 (CH-2); 41.9 (CH_2-3); 14.6 (CH_3S-4′).

5-hydroxy-2-(4-(methylthio)phenyl)chroman-4-one (**FV7**) was obtained as racemic mixtures because these was synthesized from chalcone (**C7**) (1.82 mmol), NaOAc (12.7 mmol) and EtOH/H_2O, then under heated to reflux as described above. This crude was then hydrolyzed with a mixture of aqueous HCl (1 N)/ethanol/isopropanol at reflux and then purified by column chromatography using ethyl acetate in hexane in a isocratic system (50 % ethyl acetate in hexane) as the mobile phase to afford **FV7** as a white powder (200.1 mg, 58% from chalcone **C7**); mp: 114.9 — 117.0 °C; HRMS *m*/*z*, observed: 287.0738; $C_{16}H_{14}O_3S$ [M + H]$^+$ requires: 287.0736. IR (film): ν_{max} cm^{-1}: 3421, 2923, 1667, 1621, 1576, 1465, 1352, 1338, 1205, 1049, 825, 729. ^{1}H-NMR (DMSO-*d*$_6$) δ ppm: 11.74 (s, 1H, ArO*H*-5); 7.45 — 7.49 (m, 3H, Ar*H*-3′,5′,7); 7.32 (d, 2H, *J* = 8.3 Hz, Ar*H*-2′,6′); 6.53 (dd, *J* = 7.8 and 6.8 Hz, Ar*H*-6,8); 5.65 (d, 1H, *J* = 12.8 and 2.8 Hz, C*H*-2); 3.41 (dd, 1H, *J* = 17.2 and 12.9 Hz, C*H*H-3); 2.88 (dd, 1H, *J* = 17.2 and 3.0 Hz, CH*H*-3); 2.49 (s, 3H,

C*H*$_3$S-4′). ^{13}C-NMR (DMSO-*d*$_6$) δ ppm: 198.7 (*C*-4); 161.2 (Ar*C*-5); 161.2 (Ar*C*-8a); 138.9 (Ar*C*-4′); 138.4 (Ar*C*-7); 134.9 (Ar*C*-1′); 127.3 (Ar*C*-3′,5′); 125.8 (Ar*C*-2′,6′); 108.8 (Ar*C*-6); 107.8 (Ar*C*-4a); 107.5 (Ar*C*-8); 78.1 (*C*H-2); 42.5(*C*H$_2$-3); 14.6 (*C*H$_3$S-4′).

7-hydroxy-2-(3,4,5-trimethoxyphenyl)chroman-4-one (**FV8**) was obtained as racemic mixtures because these was synthesized from chalcone (**C8**) (0.67 mmol), NaOAc (4.69 mmol) and EtOH/H_2O, then under heated to reflux as described above. This crude was then hydrolyzed with a mixture of aqueous HCl (1 N)/ethanol/isopropanol at reflux and then purified by column chromatography using ethyl acetate in hexane in a isocratic system (50 % ethyl acetate in hexane) as the mobile phase to afford **FV8** as a pale yellow powder (114.5 mg, 59% from chalcone **C8**); mp: 194.7 – 196.3 °C. IR (film): $_{max}$ cm^{-1}: 3336, 2940, 2839, 1660, 1596, 1509, 1462, 1423, 1369, 1329, 1279, 1239, 1151, 1124. ^{1}H-NMR (DMSO-*d*$_6$) δ ppm: 10.61 (s, 1H, Ar-O*H*-7); 7.65 (d, 1H, *J* = 8.7 Hz, Ar*H*-5); 6.85 (s, 2H, Ar*H*-2′,6′); 6.51 (dd, 1H, *J* = 8.5 and 2.0 Hz, Ar*H*-6); 6.37 (s, 1H, Ar*H*-8); 5.46 (dd, 1H, *J*=13.0 and 2.0 Hz, C*H*-2); 3.78 (s, 6H, C*H*$_3$O-3′,5′); 3.66 (s, 3H, C*H*$_3$O-4′); 3.19 (dd, 1H, *J*= 16.7 and 13.3 Hz, CH*H*-3); 2.65 (dd, 1H, *J*= 16.8 and 2.4 Hz, C*H*H-3). ^{13}C-NMR (DMSO-*d*$_6$) δ ppm: 189.9 (C-4); 164.7 (Ar*C*-1a); 163.0 (Ar*C*-7); 152.9 (Ar*C*-3′,5′); 137.4 (Ar*C*-4′); 134.6 (Ar*C*-1′); 128.4 (Ar*C*-5); 113.5 (Ar*C*-4a); 110.6 (Ar*C*-6); 104.2 (Ar*C*-2′,6′); 102.6 (Ar*C*-8); 79.3 (*C*H-2); 60.0 (*C*H$_3$O-4′); 55.9 (*C*H$_3$O-3′,5′); 43.4 (*C*H$_2$-3).

2-(3,4-dihydroxyphenyl)-5,7-dimethoxychroman-4-one (**FV9**) was obtained as racemic mixtures because these was synthesized from chalcone (**C9**) (0.93 mmol), NaOAc (6.49 mmol) and EtOH/H_2O, then under heated to reflux as described above. This crude was then hydrolyzed with a mixture of aqueous HCl (1 N)/ethanol/isopropanol at reflux and then purified by column chromatography using ethyl acetate in hexane in a isocratic system (70 % ethyl acetate in hexane) as the mobile phase to afford **FV9** as a pale yellow powder (79.2 mg, 27 % from chalcone **C9**); mp: 172.5 – 173.3 °C (dec.); HRMS *m/z*, observed: 317.1025; $C_{17}H_{16}O_6$ [M + H]$^+$ requires: 317.1020. IR (KBr): ν $_{max}$ cm^{-1}: 3396, 2977, 2932, 2840, 1645, 1609, 1571, 1518, 1455, 1426, 1388, 1346, 1280, 1218, 1200, 1161, 1118, 1073, 811, 788, 556. ^{1}H-NMR (DMSO-*d*$_6$) δ ppm: 9.03 (s, 2H, Ar-O*H*-3′,4′); 6.85 (s, 1H, Ar*H*-6′); 6.72 (s, 2H, Ar*H*-2′,5′); 6.17 (s, 2H, Ar*H*-6,8); 5.31 (dd, 1H, *J*=12.3 and 2.3 Hz, C*H*-2); 3.78 (s, 3H, C*H*$_3$O-5); 3.75 (s, 3H, C*H*$_3$O-7); 2.96 (dd, 1H, *J*= 16.2 and 12.6 Hz, CH*H*-3); 2.53 (dd, 1H, *J*= 16.4 and 2.6 Hz, C*H*H-3). ^{13}C-NMR (DMSO-*d*$_6$) δ ppm: 188.1 (4-*C*=O); 165.3 (Ar*C*-7); 164.4 (Ar*C*-5); 161.7 (Ar*C*-8a); 145.6 (Ar*C*-4′); 145.2 (Ar*C*-3′); 129.7 (Ar*C*-1′); 117.8 (Ar*C*-6′); 115.3 (Ar*C*-5′); 114.2 (Ar*C*-2′); 105.4 (Ar*C*-4a); 93.7 (Ar*C*-6); 92.7 (Ar*C*-8); 78.3 (*C*H-2); 55.8 (*C*H$_3$O-5); 55.7 (*C*H$_3$O-7); 44.8 (*C*H$_2$-3).

5,7-dihydroxy-2-(4-methoxyphenyl)chroman-4-one (**FV10**) was obtained as racemic mixtures because these was synthesized from chalcone (**C10**) (2.67 mmol), NaOAc (18.69 mmol) and EtOH/H_2O, then under heated to reflux as described above. This crude was then hydrolyzed with a mixture of aqueous HCl (1 N)/ethanol/isopropanol at reflux and then purified by column chromatography using ethyl acetate in hexane in a gradient system (0 — 50 % ethyl acetate in hexane) as the mobile phase to afford **FV10** as a white solid (175.8 mg, 23 % from chalcone **10**); mp: 195.4 — 196.5 °C (lit. [41] 198 – 200 °C. IR (KBr): ν $_{max}$ cm^{-1}: 3224, 2957, 2839, 1651, 1636, 1602, 1518, 1498, 1340, 1316, 1300, 1249, 1175, 1163, 1093, 1066, 1020, 830, 719, 659, 558. ^{1}H-NMR (DMSO-*d*$_6$) δ ppm: 12.12 (s, 1H, Ar-O*H*-5); 7.43 (d, 2H, *J* = 8.7 Hz, Ar*H*-2′,6′); 6.96 (d, 2H, *J* = 8.7 Hz, Ar*H*-3′,5′); 5.88 (dd, 2H, *J* = 4.6 and 2.2 Hz , Ar*H*-6,8); 5.49 (dd, 1H, *J*=12.6 and 3.0 Hz, C*H*-2); 3.76 (s, 3H, C*H*$_3$O-4′); 3.26 (dd, 1H, *J*=17.1 and 12.7 Hz; CH*H*-3); 2.70 (dd. 1H, *J*=17.1 and

3.0 Hz, C*H*H-3). ^{13}C-NMR (DMSO-*d*$_6$) δ ppm: 196.2 (4-*C*=O); 166.7 (Ar*C*-7); 163.5 (Ar*C*-5); 162.9 (Ar*C*-8a); 159.5 (Ar*C*-4′); 130.6 (Ar*C*-1′); 128.3 (Ar*C*-2′,6′); 113.9 (Ar*C*-3′,5′); 101.8 (Ar*C*-4a); 95.9 (Ar*C*-6); 95.0 (Ar*C*-8); 78.2 (CH-2); 55.2 (*C*H$_3$O-4′); 42.0 (*C*H$_2$-3).

5,7-dihydroxy-2-(3,4,5-trimethoxyphenyl)chroman-4-one (**FV11**) was obtained as racemic mixtures because these was synthesized from chalcone (C**11**) (4.6 mmol), NaOAc (32.2 mmol) and EtOH/H_2O, then under heated to reflux as described above. This crude was then hydrolyzed with a mixture of aqueous HCl (1 N)/ethanol/isopropanol at reflux and then purified by column chromatography using ethyl acetate in hexane in a gradient system (0 — 76 % ethyl acetate in hexane) as the mobile phase to afford **FV11** as a white solid (1015.7 mg, 51 % from chalcone **C11**); mp: 227.4 — 228.7 °C (lit. [43] 225 – 226 °C). IR (KBr): ν_{max} cm^{-1}: 3331, 3008, 2973, 2946, 2931, 1645, 1624, 1588, 1511, 1455, 1425, 1378, 1344, 1280, 1254, 1234, 1186, 1165, 1126, 1083, 1067, 834, 761, 724. ^{1}H-NMR (DMSO-*d*$_6$) δ ppm: 12.14 (s, 1H, Ar-O*H*-5); 10.83 (s, 1H, Ar-O*H*-7); 6.84 (s, 2H, Ar*H*-2′,6′); 5.92 (d, 1H, *J* = 2.2 Hz, Ar*H*-8); 5.89 (d, 1H, *J* = 2.1 Hz, Ar*H*-5); 5.46 (dd, 1H, *J* =13.1 and 2.8 Hz, C*H*-2); 3.78 (s, 6H, C*H*$_3$O-3′,5′); 3.66 (s, 3H, C*H*$_3$O-4′); 3.34 (m, 1H, CH*H*-3); 2.72 (dd, 1H, *J* = 17.2 and 2.4 Hz, C*H*H-3). ^{13}C-NMR (DMSO-*d*$_6$) δ ppm: 196.1 (C-4); 166.7 (Ar*C*-7); 163.5 (Ar*C*-5); 162.7 (Ar*C*-8a); 152.9 (Ar*C*-3′,5′); 137.5 (Ar*C*-4′); 134.2 (Ar*C*-1′); 104.2 (Ar*C*-2′,6′); 101.7 (Ar*C*-4a); 95.9 (Ar*C*-6); 95.0 (Ar*C*-8); 78.8 (C*H*-2); 60.0 (*C*H$_3$O-4′); 56.0 (*C*H$_3$O-3′,5′); 42.2 (*C*H$_2$-3).

Flavones (**FO1 – FO11**).

2-(4-hydroxy-3-methoxyphenyl)-5,7-dimethoxy-4*H*-chromen-4-one (**FO1**) was obtained from chalcone **C1** (2.63 mmol) using iodine (2.63 mmol), potassium iodide (2.63 mmol) and pyridine as solvent (6 mL), then under heated at 120 °C for 13 h, as described above. This crude was then hydrolyzed with a mixture of aqueous HCl (1 N)/ethanol/isopropanol at reflux and then purified by column chromatography using ethyl acetate in hexane in a gradient system (0 — 70 % ethyl acetate in hexane) as the mobile phase to afford **FO1** as a yellow solid (215.9 mg, 25 % from chalcone **C1**); mp: 218.9 — 219.3 °C (lit. [44] 220 – 221 °C); IR (KBr): ν_{max} cm^{-1}: 3443, 3116, 1645, 1596, 1528, 1491, 1467, 1427, 1355, 1295, 1263, 1219, 1200, 1163, 1134, 1059, 1025, 839, 818. ^{1}H-NMR (DMSO-*d*$_6$) δ ppm: 9.79 (s, 1H, Ar-O*H*-4′); 7.52 — 7.49 (m, 2H, Ar*H*-2′,6′); 6.90 (d, 1H, *J* = 8.9 Hz, Ar*H*-5′); 6.84 (d, 1H, *J* = 2.3 Hz, Ar*H*-8); 6.68 (s, 1H, C*H*-3); 6.48 (d, 1H, *J* = 2.3 Hz, Ar*H*-6); 3.89 — 3.88 (m, 6H, C*H*$_3$O-7, 3′); 3.81 (s, 3H, C*H*$_3$O-5). ^{13}C-NMR (DMSO-*d*$_6$) δ ppm: 175.7 (4-*C*=O); 163.5 (Ar*C*-7); 160.2 (Ar*C*-5); 160.0 (Ar*C*-2); 159.1 (Ar*C*-8a); 150.0 (Ar*C*-4′); 148.0 (ArC-3′); 121.7 (Ar*C*-1′); 119.6 (Ar*C*-6′); 115.6 (Ar*C*-5′); 109.8 (Ar*C*-2′); 108.2 (Ar*C*-4a); 106.4 (*C*-3); 96.1 (Ar*C*-6); 93.4 (Ar*C*-8); 56.0-55.9 (*C*H$_3$O-5,7,3′).

2-(3,4-dihydroxyphenyl)-5,7-dihydroxy-4*H*-chromen-4-one (**FO2**) (luteolin) was obtained from chalcone **C2** (2.16 mmol) using iodine (2.16 mmol), potassium iodide (2.16 mmol) and pyridine as solvent (6 mL), then under heated at 120 °C for 18 h, as described above. This crude was then hydrolyzed with a mixture of aqueous HCl (1 N)/ethanol/isopropanol at reflux and then purified by column chromatography using ethyl acetate in hexane in a gradient system (0 — 70 % ethyl acetate in hexane) as the mobile phase to afford **FO2** as a yellow solid (82.7 mg, 15 % from chalcone **C2**); mp: > 300 °C dec. (lit. [45] 329 — 330 °C dec.); IR (KBr): ν_{max} cm^{-1}: 3420, 2926, 1658, 1609, 1508, 1446, 1362, 1261, 1164, 1031, 838. ^{1}H-NMR (DMSO-*d*$_6$) δ ppm: 12.96 (s, 1H, Ar-O*H*-5); 10.82 (s, 1H, Ar-O*H*-7); 9.91 (s, 1H, Ar-O*H*-4′); 9.40 (s, 1H, Ar-O*H*-3′); 7.41 — 7.38 (m, 2H, Ar*H*-2′,6′); 6.87 (d, 1H, *J* =

8.2 Hz, Ar*H*-5′); 6.66 (s, 1H, C*H*-3); 6.43 (d, 1H, *J* = 2.1 Hz, Ar*H*-8); 6.18 (d, 1H, *J* =2.2 Hz, Ar*H*-6). ^{13}C-NMR (DMSO-d_6) δ ppm: 181.6 (4-*C*=O); 164.1 (Ar*C*-7); 163.9 (*C*-2); 161.5 (Ar*C*-5); 157.3 (Ar*C*-8a); 149.7 (Ar*C*-3′); 145.7 (ArC-4′); 121.5 (Ar*C*-1′); 119.0 (Ar*C*-6′); 116.0 (Ar*C*-5′); 113.4 (Ar*C*-2′); 103.7 (Ar*C*-4a); 102.8 (*C*-3); 98.8 (Ar*C*-6); 93.8 (Ar*C*-8).

5,7-dihydroxy-2-(4-hydroxy-3-methoxyphenyl)-4*H*-chromen-4-one (**FO3**) (Chrysoeriol) was obtained from chalcone **C3** (1.15 mmol) using iodine (1.15 mmol), potassium iodide (1.15 mmol) and pyridine as solvent (6 mL), then under heated at 120 °C for 19 h, as described above. This crude was then hydrolyzed with a mixture of aqueous HCl (1 N)/ethanol/isopropanol at reflux and then purified by column chromatography using ethyl acetate in hexane in a gradient system (0 — 60 % ethyl acetate in hexane) as the mobile phase to afford **FO3** as a yellow solid (58.7 mg, 17 % from chalcone **C3**); mp: > 300 °C dec. (lit. [46] 336 °C.); IR (KBr): ν_{max} cm^{-1}: 3344, 2924, 2852, 1650, 1624, 1564, 1510, 1435, 1350, 1295 1272, 1208, 1169, 1118, 1033, 837, 766. ^{1}H-NMR (DMSO-d_6) δ ppm: 12.95 (s, 1H, Ar-O*H*-5); 10.82 (s, 1H, Ar-O*H*-7); 9.95 (s, 1H, Ar-O*H*-4′); 7.58-7.54 (m, 2H, Ar*H*-2′,6′); 6.92 (d, 1H, *J* = 9.0 Hz, Ar*H*-5′); 6.88 (s, 1H, C*H*-3); 6.50 (d, 1H, *J* = 2.2 Hz, Ar*H*-8); 6.18 (d, 1H, J_1=2,0 Hz, Ar*H*-6); 3.88 (s, 3H, CH_3O-3′). ^{13}C-NMR (DMSO-d_6) δ ppm: 181.8 (4-*C*=O); 164.1 (Ar*C*-7); 163.6 (*C*-2); 161.4 (Ar*C*-5); 157.3 (Ar*C*-8a); 150.7 (Ar*C*-4′); 148.0 (ArC-3′); 121.5 (Ar*C*-1′); 120.3 (Ar*C*-6′); 115.8 (Ar*C*-5′); 110.2 (Ar*C*-2′); 103.7 (Ar*C*-4a); 103.2 (*C*-3); 98.8 (Ar*C*-6); 94.0 (Ar*C*-8); 56.0 (CH_3O-3′).

5,7-dihydroxy-2-(2,4,6-trimethoxyphenyl)-4*H*-chromen-4-one (**FO4**) was obtained from chalcone **C4** (7.54 mmol) using iodine (7.54 mmol), potassium iodide (7.54 mmol) and pyridine as solvent (8 mL), then under heated at 120 °C for 17 h, as described above. This crude was then hydrolyzed with a mixture of aqueous HCl (1 N)/ethanol/isopropanol at reflux and then purified by column chromatography using ethyl acetate in hexane in a gradient system (0 — 50 % ethyl acetate in hexane) as the mobile phase to afford **FO4** as a yellow solid (726.9 mg, 28 % from chalcone **C4**); mp: 241.6 — 242.5 °C (lit. without data); IR (KBr): ν_{max} cm^{-1}: 3453, 2924, 2849, 1648, 1620, 1557, 1508, 1471, 1354, 1224, 1207, 1168, 1142, 1033, 847, 796, 762. ^{1}H-NMR (DMSO-d_6) δ ppm: 12.83 (s, 1H, Ar-O*H*-5); 10.82 (s, 1H, Ar-O*H*-7); 6.34 (s, 2H, Ar*H*-3′,5′); 6.30 (d, 3H, *J* = 2.0 Hz, Ar*H*-8); 6.19 (d, 3H, *J* = 2.0 Hz, Ar*H*-6); 6.16 (s, 1H, C*H*-3); 3.84 (s, 3H, C*H*3O-4′); 3.76 (s, 6H, C*H*3O-2′,6′). ^{13}C-NMR (DMSO-d_6) δ ppm: 181.6 (4-*C*=O); 164.2 (Ar*C*-7); 163.3 (Ar*C*-4′); 161.5 (Ar*C*-5); 161.4 (Ar*C*-2); 159.0 (Ar*C*-2′,6′); 158.3 (Ar*C*-8a); 112.2 (*C*H-3); 103.7 (Ar*C*-4a); 103.2 (Ar*C*-1′); 98.8 (Ar*C*-6); 93.8 (Ar*C*-8); 91.1 (Ar*C*-3′,5′); 56.1 (*C*H3O-2′,6′); 55.6 (*C*H3O-4′).

5,7-dihydroxy-2-(4-hydroxyphenyl)-4*H*-chromen-4-one (**FO5**; apigenin) was obtained from chalcone **C5** (1.24 mmol) using iodine (1.24 mmol), potassium iodide (1.24 mmol) and pyridine as solvent (8 mL), then under heated at 120 °C for 11 h, as described above. This crude was then hydrolyzed with a mixture of aqueous HCl (1 N)/ethanol/isopropanol at reflux and then purified by column chromatography using ethyl acetate in hexane in a gradient system (0 — 40 % ethyl acetate in hexane) as the mobile phase to afford **FO5** as a yellow solid (80.4 mg, 24 % from chalcone **C5**); mp: > 330 °C dec. (lit. [47] 356 — 357 °C); IR (KBr): ν_{max} cm^{-1}: 3284, 3096, 2926, 2634, 1651, 1606, 1554, 1500, 1439, 1354, 1268, 1244, 1181, 1031, 829, 742, 579. ^{1}H-NMR (DMSO-d_6) δ ppm: 12.95 (s, 1H, Ar-O*H*-5); 10.81 (s, 1H, Ar-O*H*-7); 10.34 (s, 1H, Ar-O*H*-4′); 7.91 (d, 2H, *J* =8.8 Hz, Ar*H*-2′,6′); 6.91 (d, 2H, *J* = 8.8 Hz, Ar*H*-3′,5′); 6.77 (s, 1H, C*H*-3); 6.47 (d, 1H, *J* = 2.0 Hz, Ar*H*-8); 6.18 (d, 1H, *J* = 2.0 Hz, Ar*H*-6). ^{13}C-NMR (DMSO-d_6) δ ppm: 181.7 (4-

C=O); 164.1 (Ar*C*-7); 163.7 (Ar*C*-2); 161.4 (Ar*C*-5); 161.1 (Ar*C*-4′); 157.3 (Ar*C*-8a); 128.4 (Ar*C*-2′,6′); 121.2 (Ar*C*-1′); 115.9 (Ar*C*-3′,5′); 103.6 (Ar*C*-4a); 102.8 (*C*H-3); 98.8 (Ar*C*-6); 93.9 (Ar*C*-8).

5,7-dihydroxy-2-(4-(methylthio)phenyl)-4*H*-chromen-4-one (**FO6**) was obtained from chalcone **C6** (2.89 mmol) using iodine (2.89 mmol), potassium iodide (2.89 mmol) and pyridine as solvent (8 mL), then under heated at 120 °C for 14 h, as described above. This crude was then hydrolyzed with a mixture of aqueous HCl (1 N)/ethanol/isopropanol at reflux and then purified by column chromatography using ethyl acetate in hexane in a gradient system (0 — 40 % ethyl acetate in hexane) as the mobile phase to afford **FO6** as a yellow solid (121.5 mg, 14 % from chalcone **C6**); mp: 230 — 232 °C; HRMS *m*/*z*, observed: 301.0527; $C_{16}H_{12}O_4S$ [M + H]$^+$ requires: 301.0529; IR (KBr): ν_{max} cm^{-1}: 2921, 2849, 2705, 2622, 1655, 1610, 1563, 1501, 1487, 1424, 1354, 1278, 1249, 1168, 1101, 1029, 1012, 908, 851, 826, 806, 748, 729, 678, 525. ^{1}H-NMR (DMSO-*d*$_6$) δ ppm: 12.86 (s, 1H, ArO*H*-5); 10.8 (s, 1H, ArO*H*-7); 7.98 (d, 2H, *J* = 8.6 Hz, Ar*H*-2′,6′); 7.40 (d, 2H, *J* = 8.6 Hz, Ar*H*-3′,5′); 6.93 (s, 1H, C*H*-3); 6.50 (d, 1H, *J* =2.0 Hz, Ar*H*-8); 6.20 (d, 1H, *J* =2.0 Hz, Ar*H*-6); 2.54 (s, 3H, C*H*$_3$S-4′). ^{13}C-NMR (DMSO-*d*$_6$) δ ppm: 181.7 (4-*C*=O); 164.3 (Ar*C*-7); 162.9 (Ar*C*-2); 161.4 (Ar*C*-5); 157.3 (Ar*C*-8a); 144.0 (Ar*C*-4′); 126.7 (Ar*C*-2′,6′); 126.6 (Ar*C*-1′); 125.5 (Ar*C*-3′,5′); 104.3 (*C*H-3); 103.9 (Ar*C*-4a); 98.9 (Ar*C*-6); 94.0 (Ar*C*-8); 14.0 (C*H*$_3$S-4′).

5-hydroxy-2-(4-(methylthio)phenyl)-4H-chromen-4-one was (**FO7**) obtained from chalcone **C7** (1.71 mmol) using iodine (1.71 mmol), potassium iodide (1.71 mmol) and pyridine as solvent (6 mL), then under heated at 120 °C for 10 h, as described above. This crude was then hydrolyzed with a mixture of aqueous HCl (1 N)/ethanol/isopropanol at reflux and then purified by column chromatography using methylene chloride in a isocratic system (100 % methylene chloride) as the mobile phase to afford **FO7** as a yellow solid (286.9 mg, 59 % from chalcone **C7**); mp: 171.2 — 172.2 °C (lit. [31] 166 — 167 °C); HRMS *m*/*z*, observed: 285.0588; $C_{16}H_{12}O_3S$ [M + H]$^+$ requires: 285.0580; IR (KBr): ν_{max} cm^{-1}: 3447, 3066, 2920, 1651, 1615, 1597, 1582, 1471, 1418, 1361, 1301, 1261, 1229, 1095, 1057, 996, 820, 800, 752. ^{1}H-NMR (DMSO-*d*$_6$) δ ppm: 12.70 (s, 1H, ArO*H*-5); 8.03 (d, 2H, *J* = 8.6 Hz, Ar*H*-2′,6′); 7.67 (dd, 1H, *J* = 8.4 and 8.3 Hz, Ar*H*-7); 7.42 (d, 2H, *J* = 8.6 Hz, Ar*H*-3′,5′); 7.19 (dd, 1H, *J* = 8.9 and 0.6 Hz, Ar*H*-8); 7.08 (s, 1H, C*H*-3); 6.80 (d, 1H, *J* = 7.7 Hz, Ar*H*-6); 2.55 (s, 3H, C*H*$_3$S-4′). ^{13}C-NMR (DMSO-*d*$_6$) δ ppm: 183.1 (4-*C*=O); 163.9 (Ar*C*-2); 159.8 (Ar*C*-5); 155.8 (Ar*C*-8a); 144.6 (Ar*C*-4′); 135.9 (Ar*C*-7); 126.9 (Ar*C*-2′,6′); 126.4 (Ar*C*-1′); 125.5 (Ar*C*-3′,5′); 111.0 (Ar*C*-6); 110.1 (Ar*C*-4a); 107.5 (Ar*C*-8); 104.8 (*C*H-3); 14.0 (*C*H$_3$S-4′).

7-hydroxy-2-(3,4,5-trimethoxyphenyl)-4*H*-chromen-4-one (**FO8**) was obtained from chalcone **C8** (2.67 mmol) using iodine (2.67 mmol), potassium iodide (2.67 mmol) and pyridine as solvent (8 mL), then under heated at 120 °C for 6 h, as described above. This crude was then hydrolyzed with a mixture of aqueous HCl (1 N)/ethanol/isopropanol at reflux and then purified by column chromatography using ethyl acetate in hexane in a gradient system (0 — 70 % ethyl acetate in hexane) as the mobile phase to afford **FO8** as a yellow solid (87.7 mg, 10 % from chalcone **C8**); mp: 263 — 265 °C dec. (lit. [48] 279 — 280 °C; IR (KBr): ν_{max} cm^{-1}: 3446, 2925, 2852, 1641, 1588, 1557, 1503, 1459, 1416, 1382, 1339, 1315, 1255, 1175, 1130, 1000, 839, 816, 781. ^{1}H-NMR (DMSO-*d*$_6$) δ ppm: 10.76 (s, 1H, Ar-O*H*-7); 7.87 (d, 1H, *J* = 8.7 Hz, Ar*H*-5); 7.32 (s, 2H, Ar*H*-2′,6′); 7.05 (d, 1H, *J* = 2.2 Hz, Ar*H*-8); 6.99 (s, 1H, C*H*-3); 6.92 (dd, 1H, *J* = 8.8 and 2.2 Hz, Ar*H*-6); 3.90 (s, 6H, C*H*$_3$O-3′,5′); 3.73 (s, 3H, C*H*$_3$O-4′). ^{13}C-NMR (DMSO-*d*$_6$) δ ppm: 176.4 (4-*C*=O); 162.7

(Ar*C*-8a); 161.8 (Ar*C*-2); 157.4 (Ar*C*-7); 153.2 (Ar*C*-3′,5′); 140.4 (Ar*C*-4′); 126.6 (Ar*C*-1′); 126.4 (Ar*C*-5); 116.1 (Ar*C*-4a); 115.0 (Ar*C*-6); 106.5 (Ar*C*-3); 103.9 (Ar*C*-2′,6′); 102.7 (Ar*C*-8); 60.2 (CH_3O-4′); 56.3 (CH_3O-3′,5′).

2-(3,4-dihydroxyphenyl)-5,7-dimethoxy-4*H*-chromen-4-one (**FO9**; luteolin 5,7-dimethyl ether) was obtained from chalcone **C9** (1.2 mmol) using iodine (1.2 mmol), potassium iodide (1.2 mmol) and pyridine as solvent (6 mL), then under heated at 120 °C for 16 h, as described above. This crude was then hydrolyzed with a mixture of aqueous HCl (1 N)/ethanol/isopropanol at reflux and then purified by column chromatography using methanol in methylene chloride in a isocratic system (90 % methylene chloride acetate / 10 % methanol) as the mobile phase to afford **FO9** as a yellow solid (86.7 mg, 23 % from chalcone **C9**); mp: 247.2 — 250.0 °C dec. (lit. [49] without mp data); IR (KBr): ν_{max} cm^{-1}: 3460, 2924, 2849, 1640, 1603, 1556, 1515, 1496, 1457, 1399, 1360, 1315, 1277, 1204, 1164, 1137, 1057, 1032, 863, 827, 783, 631. ^{1}H-NMR (DMSO-d_6) δ ppm: 9.53 (br. s, 2H, Ar-O*H*-3′,4′); 7.35 — 7.34 (m, 2H, Ar*H*-2′,6′); 6.86 (d, 1H, *J* = 8.9 Hz, Ar*H*-5′); 6.76 (d, 1H, *J* = 2.0 Hz, Ar*H*-8); 6.48 (d, 1H, *J* =1.9 Hz, Ar*H*-6); 6.45 (s, 1H, C*H*-3); 3.88 (CH_3O-5′); 3.81 (CH_3O-7′). ^{13}C-NMR (DMSO-d_6) δ ppm: 175.5 (4-*C*=O); 163.5 (Ar*C*-7); 160.2 (Ar*C*-2,5); 159.1 (Ar*C*-8a); 148.9 (Ar*C*-3′); 145.6 (Ar*C*-4′); 121.7 (Ar*C*-1′); 118.2 (Ar*C*-6′); 115.9 (Ar*C*-5′); 113.0 (Ar*C*-2′); 108.2 (Ar*C*-4a); 106.2 (*C*H-3); 96.1 (Ar*C*-6); 93.2 (Ar*C*-8); 56.0 (CH_3O-5′); 55.9 (CH_3O-7′).

5,7-dihydroxy-2-(4-methoxyphenyl)-4*H*-chromen-4-one (**FO10**; acacetin) was obtained from chalcone **C10** (1.34 mmol) using iodine (1.34 mmol), potassium iodide (1.34 mmol) and pyridine as solvent (7 mL), then under heated at 120 °C for 18 h, as described above. This crude was then hydrolyzed with a mixture of aqueous HCl (1 N)/ethanol/isopropanol at reflux and then purified by column chromatography using ethyl acetate in hexane in a isocratic system (50 % ethyl acetate) as the mobile phase to afford **FO10** as a yellow solid (110.5 mg, 29 % from chalcone **C10**); mp: 256.5 — 257.8 °C dec. (lit. [50] 256.0 — 257.8 °C); IR (KBr): ν_{max} cm^{-1}: 3156, 1651, 1606, 1563, 1509, 1429, 1370, 1318, 1299, 1267, 1239, 1186, 1166, 1120, 1032, 826. ^{1}H-NMR (DMSO-d_6) δ ppm: 12.91 (s, 1H, Ar-O*H*-5); 10.85 (s, 1H, Ar-O*H*-7); 8.02 (d, 2H, *J* =8.0 Hz, Ar*H*-2′,6′); 7.09 (d, 2H, *J* = 8.4 Hz, Ar*H*-3′,5′); 6.86 (s, 1H, C*H*-3); 6.49 (s, 1H, Ar*H*-8); 6.19 (s, 1H, Ar*H*-6); 3.84 (s, 3H, CH_3O-4′). ^{13}C-NMR (DMSO-d_6) δ ppm: 181.8 (4-*C*=O); 164.2 (Ar*C*-7); 163.5 (Ar*C*-2); 162.3 (Ar*C*-4′); 161.4 (Ar*C*-5); 157.3 (Ar*C*-8a); 128.3 (Ar*C*-2′,6′); 122.8 (Ar*C*-1′); 114.5 (Ar*C*-3′,5′); 103.5 (*C*H-3); 103.3 (Ar*C*-4a); 98.9 (Ar*C*-6); 94.0 (Ar*C*-8); 55.5 (CH_3O-4′).

5,7-dihydroxy-2-(3,4,5-trimethoxyphenyl)-4*H*-chromen-4-one (**FO11)** was obtained from chalcone **C11** (1.39 mmol) using iodine (1.39 mmol), potassium iodide (1.39 mmol) and pyridine as solvent (6 mL), then under heated at 100 °C for 22 h, as described above. This crude was then hydrolyzed with a mixture of aqueous HCl (1 N)/ethanol/isopropanol at reflux and then purified by column chromatography using ethyl acetate in hexane in a isocratic system (50 % ethyl acetate) as the mobile phase to afford **FO11** as a yellow solid (81.4 mg, 17 % from chalcone **C11**); mp: 264.8 — 266.0 °C dec. (lit. [51] 264.0 — 265 °C); IR (KBr): ν_{max} cm^{-1}: 3408, 3251, 2920, 2849, 1651, 1621, 1592, 1505, 1442, 1433, 1363, 1339, 1290, 1257, 1179, 1162, 1135, 1052, 1035, 1003, 834. ^{1}H-NMR (DMSO-d_6) δ ppm: 12.85 (s, 1H, Ar-O*H*-5); 10.89 (s, 1H, Ar-O*H*-7); 7.33 (s, 2H, Ar*H*-2′,6′); 7.07 (s, 1H, C*H*-3); 6.56 (d, 1H, *J* = 2.0 Hz, Ar*H*-8); 6.42 (d, 1H, *J* = 2.1 Hz, Ar*H*-5); 3.91 (s, 6H, CH_3O-3′,5′); 3.75 (s, 3H, CH_3O-4′). ^{13}C-NMR (DMSO-d_6) δ ppm: 181.9 (C-

4); 164.4 (Ar*C*-7); 163.0 (Ar*C*-2); 161.4 (ArC-5) 157.4 (Ar*C*-8a); 153.2 (Ar*C*-3′,5′); 140.7 (Ar*C*-4′); 126.0 (Ar*C*-1′); 105.0 (*C*H-3); 104.1 (Ar*C*-2′,6′); 103.8 (Ar*C*-4a); 99.0 (Ar*C*-6); 94.3 (Ar*C*-8); 60.2 (CH_3O-4′); 56.3 (CH_3O-3′,5′).

Spectra of all synthezised compounds

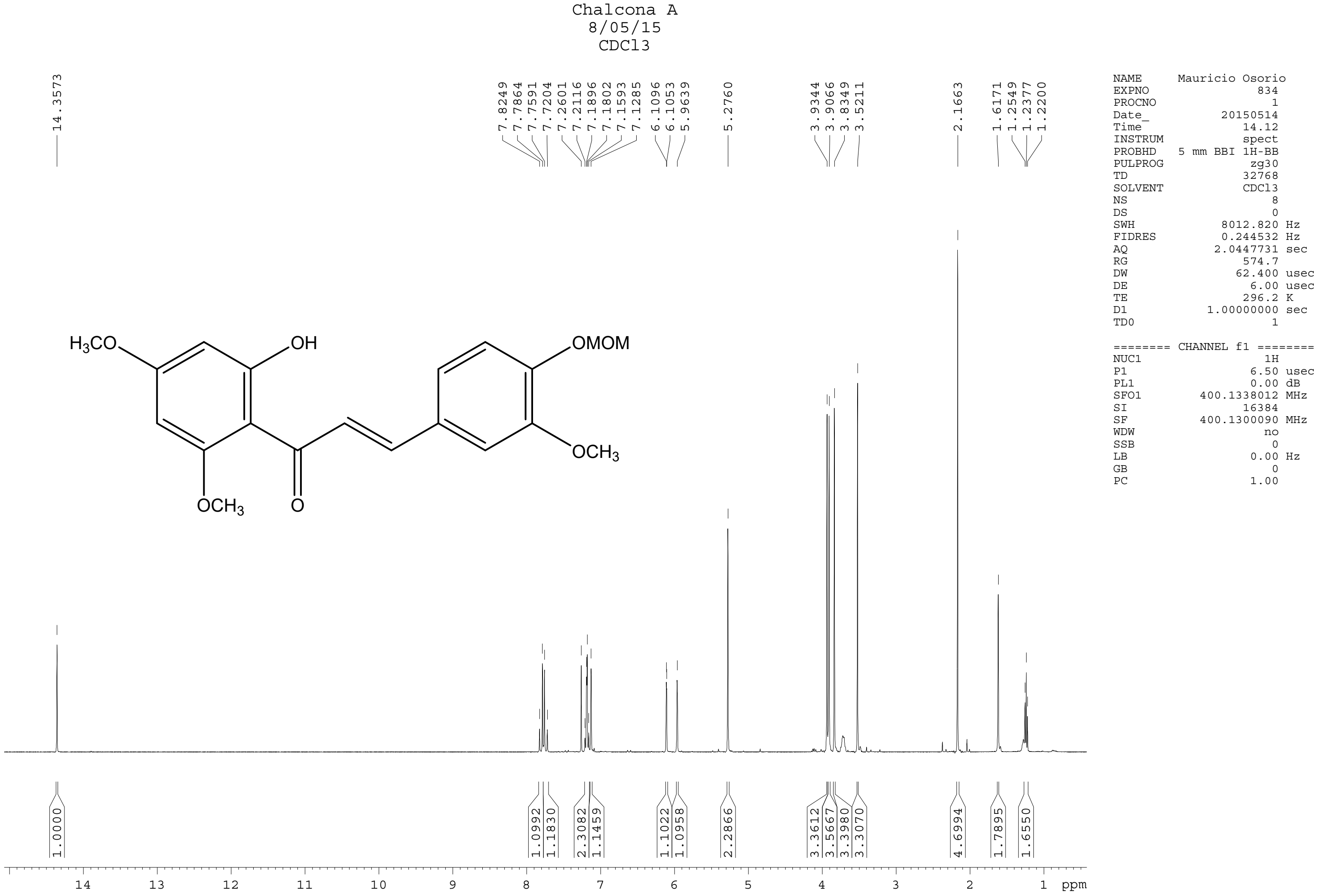

Figure S2. ^{1}H-NMR of **C1** ($CDCl_3$).

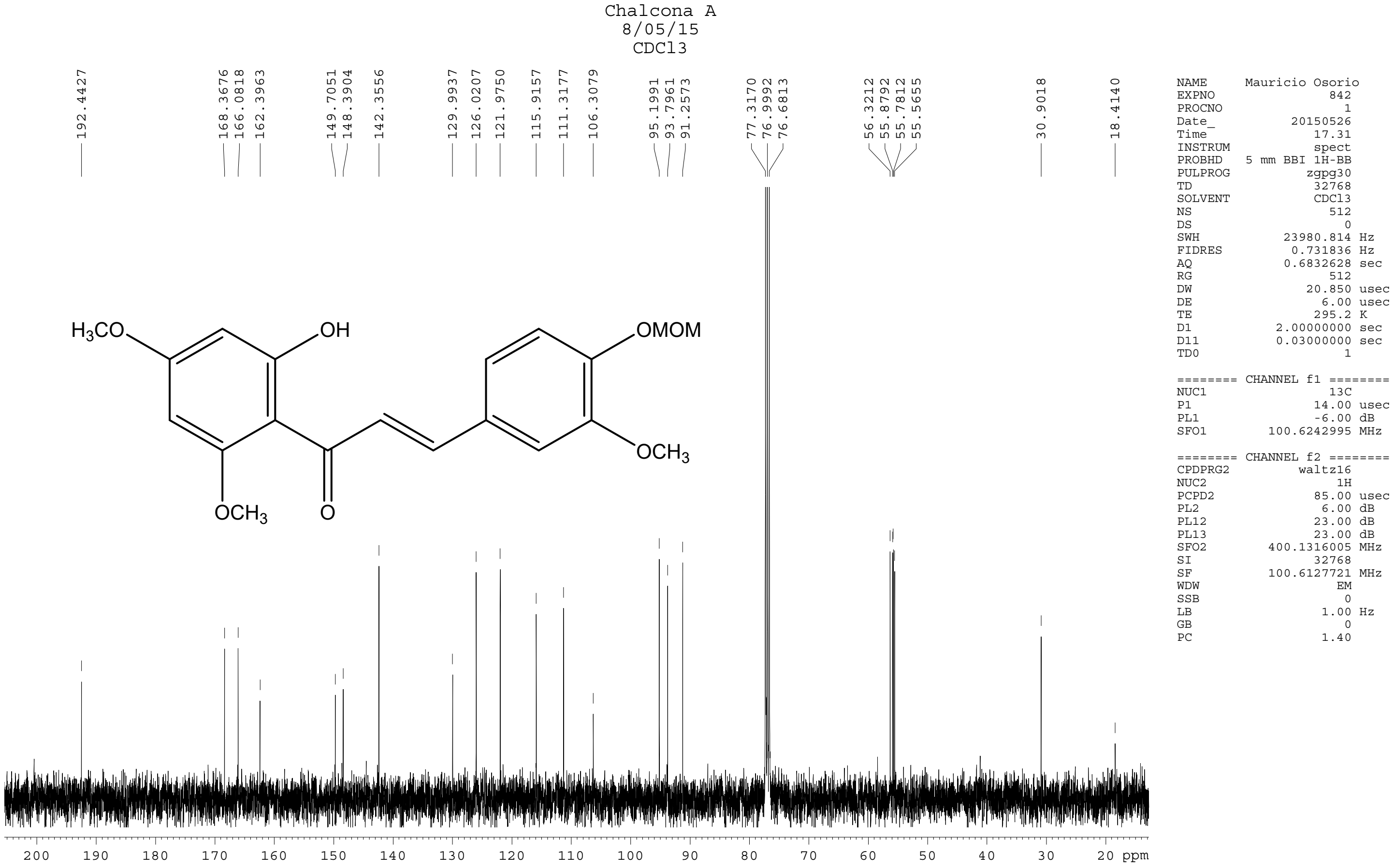

Figure S3. ^{13}C-NMR of **C1**

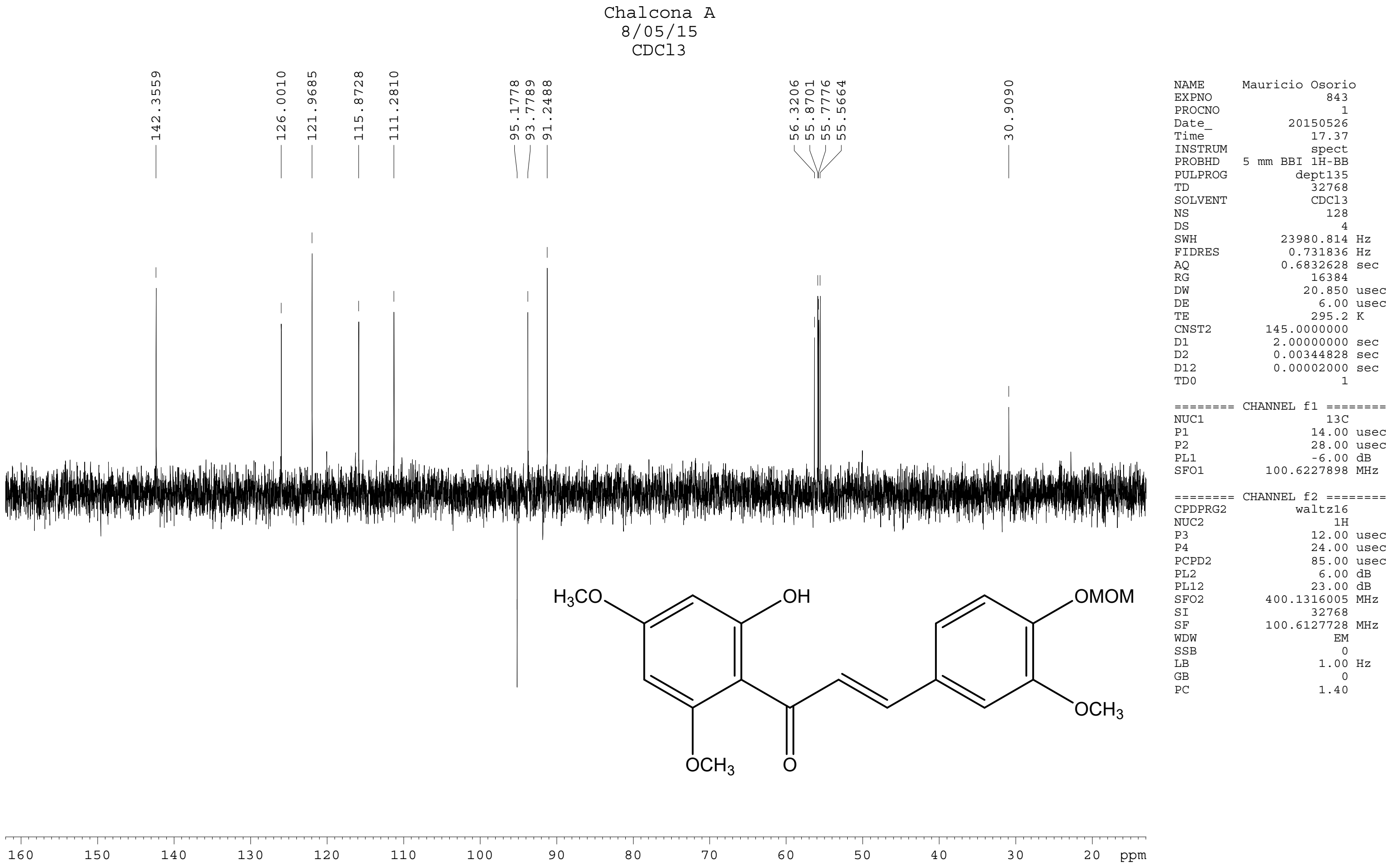

Figure S4. DEPT-135 of **C1**

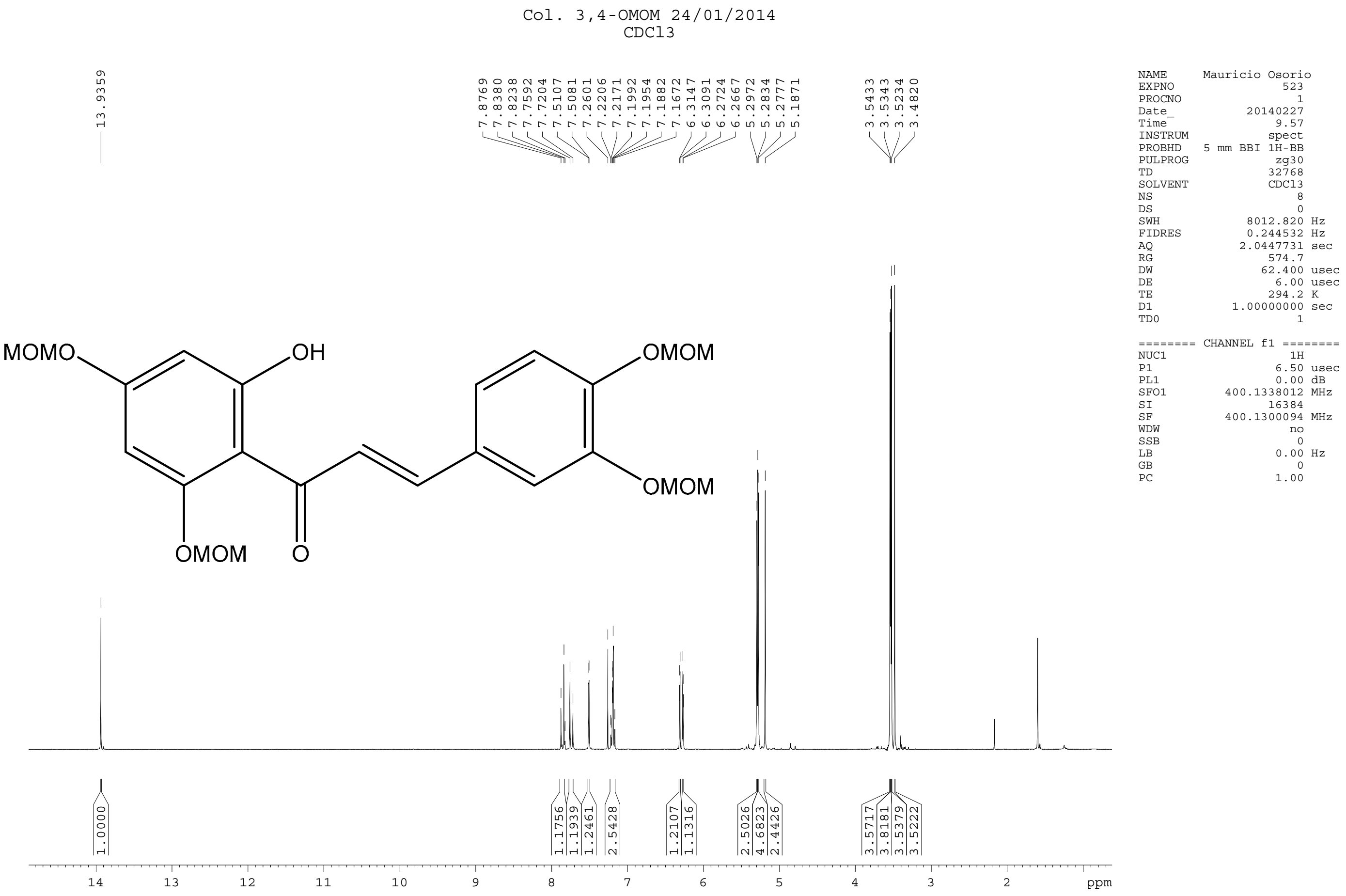

Figure S5. ^{1}H-NMR of **C2** ($CDCl_3$).

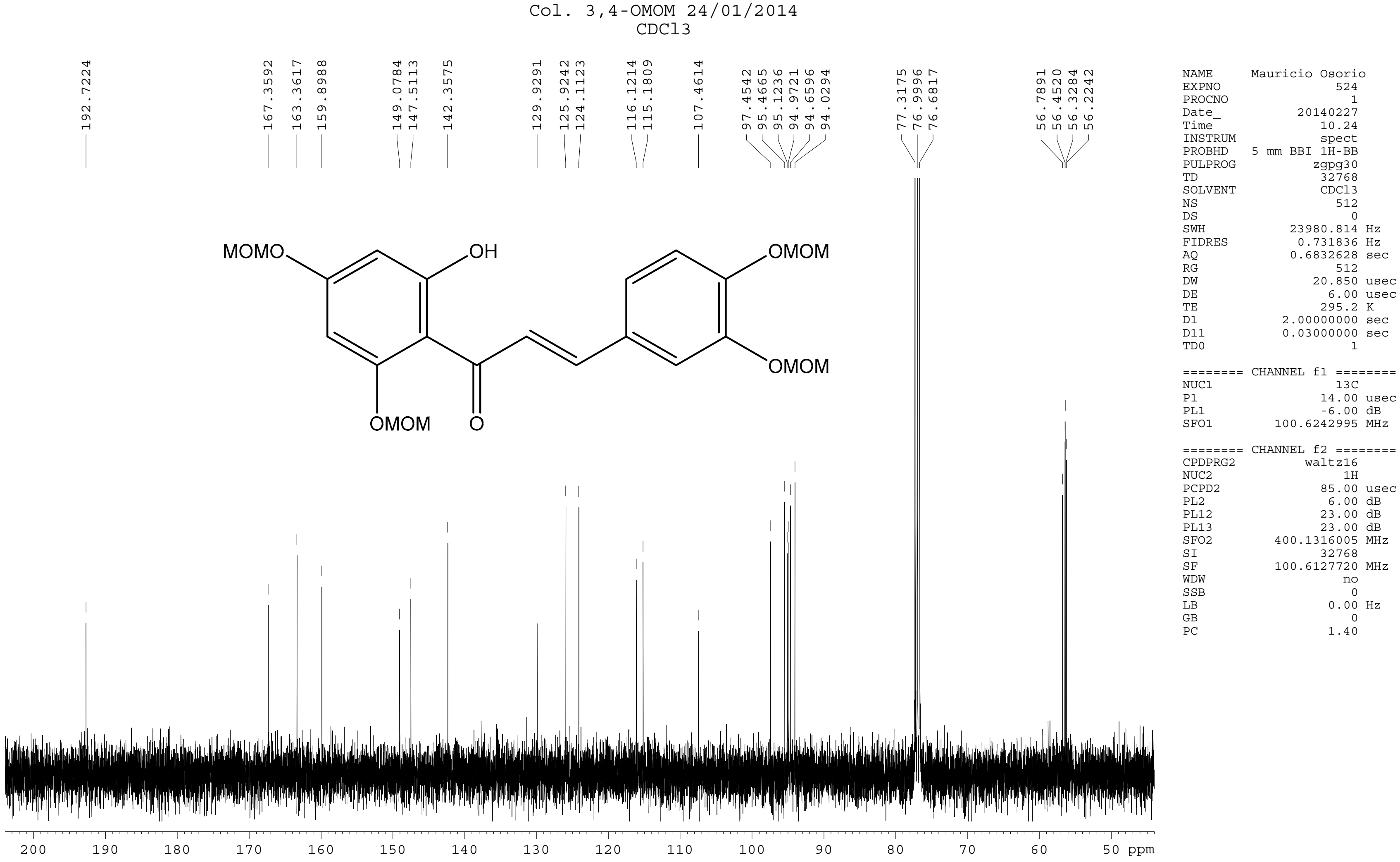

Figure S6. ^{13}C-NMR of **C2**

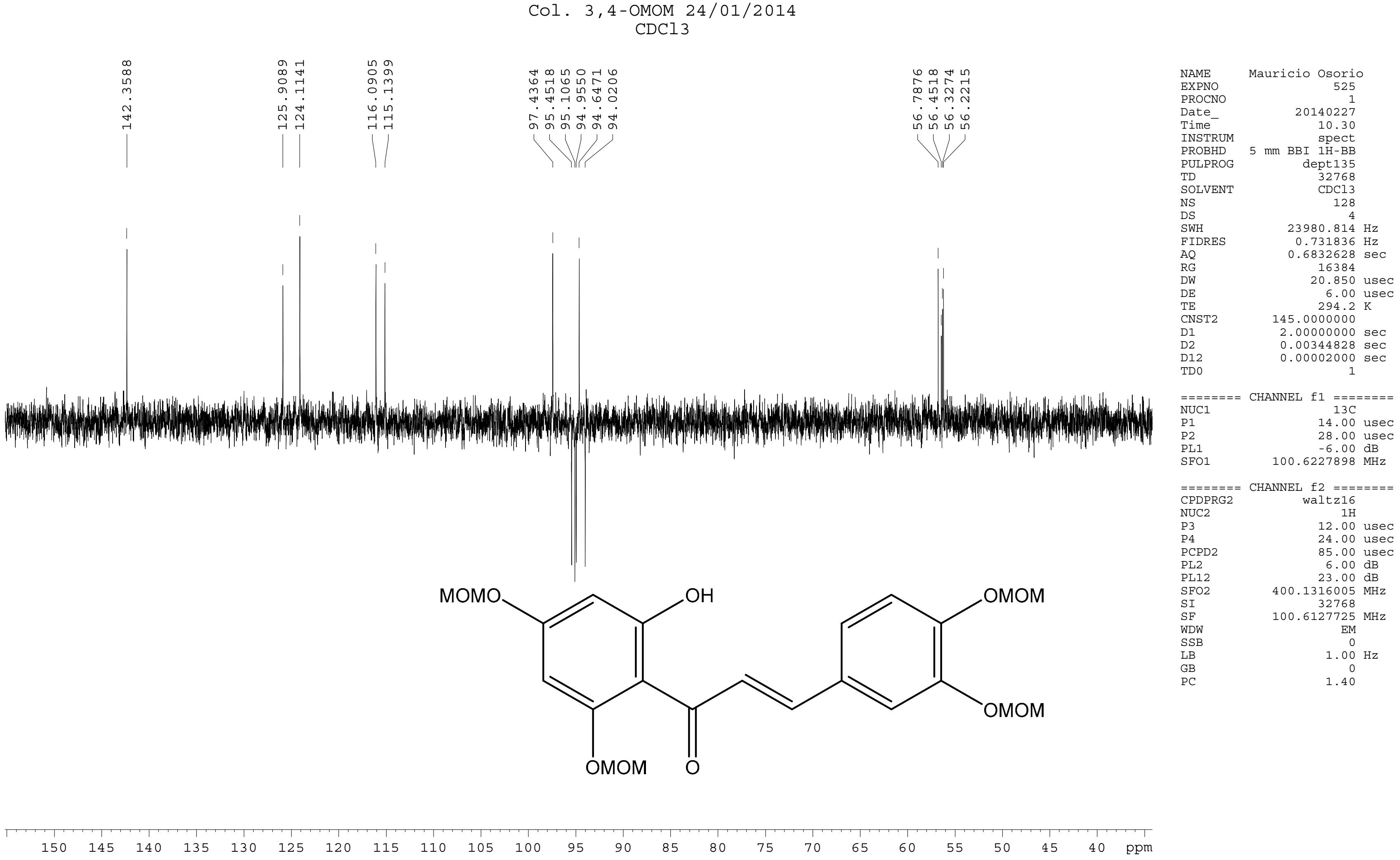

Figure S7. DEPT-135 of **C2.**

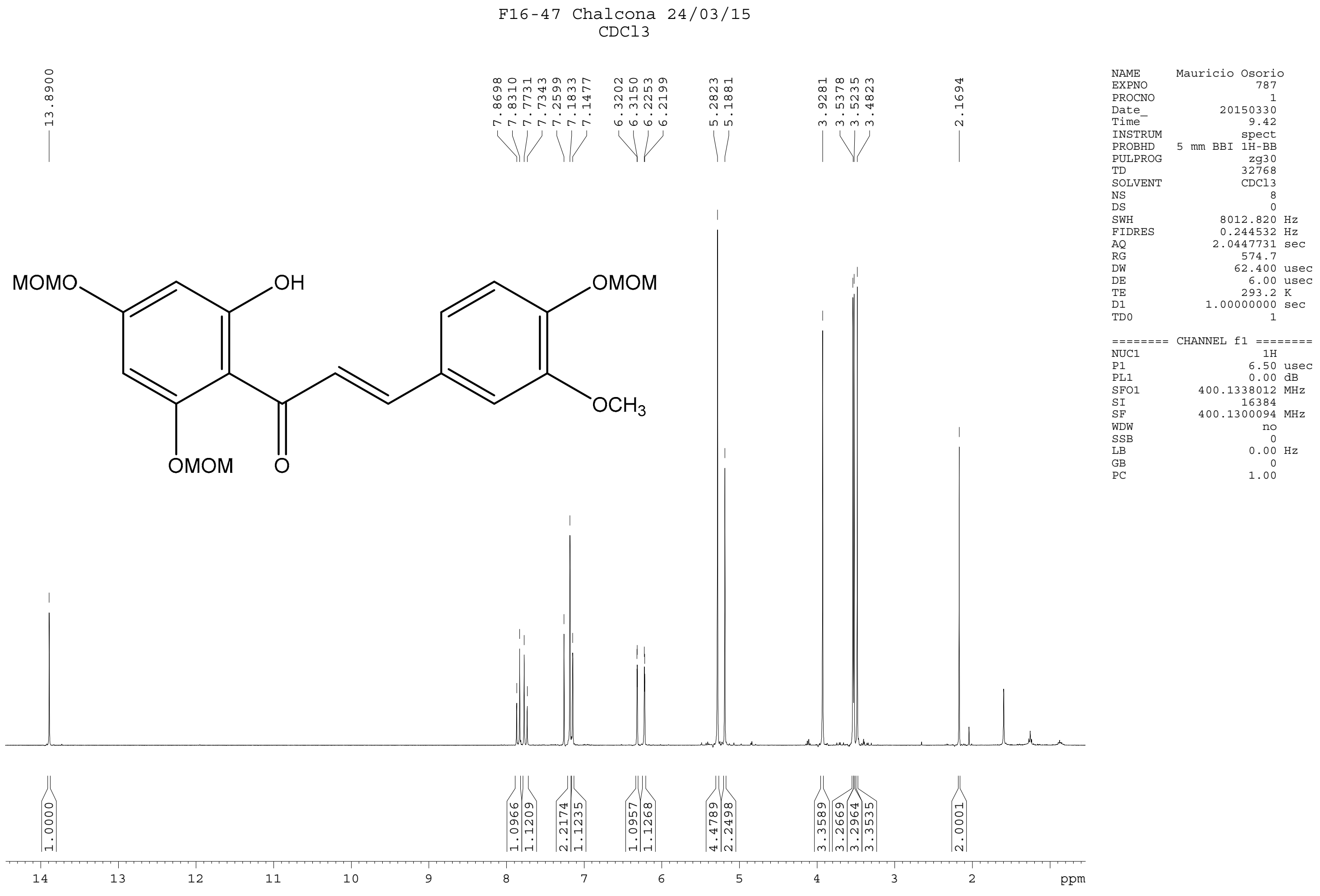

Figure S8. ^{1}H-NMR of **C3** ($CDCl_3$).

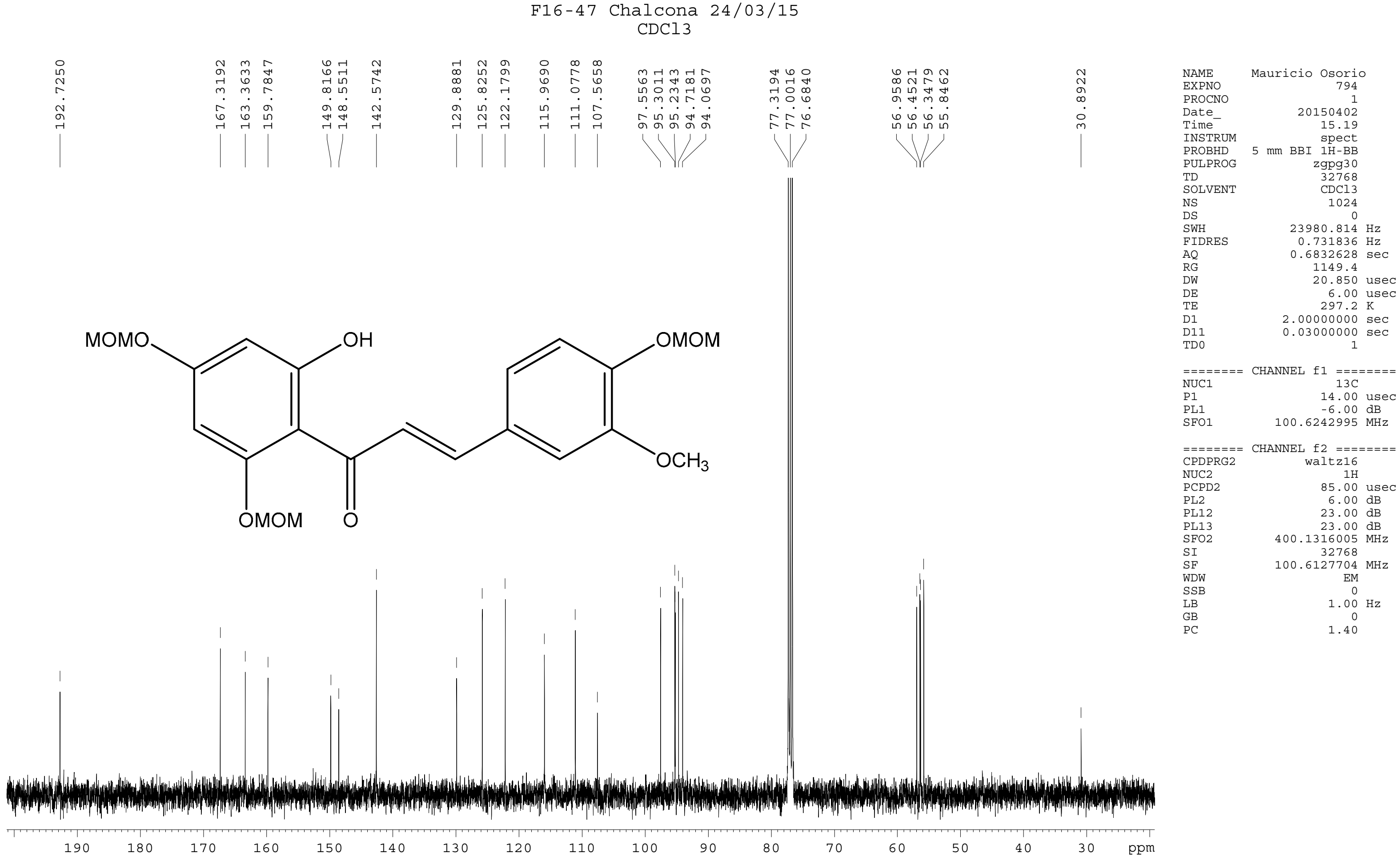

Figure S9. ^{13}C-NMR of **C3.**

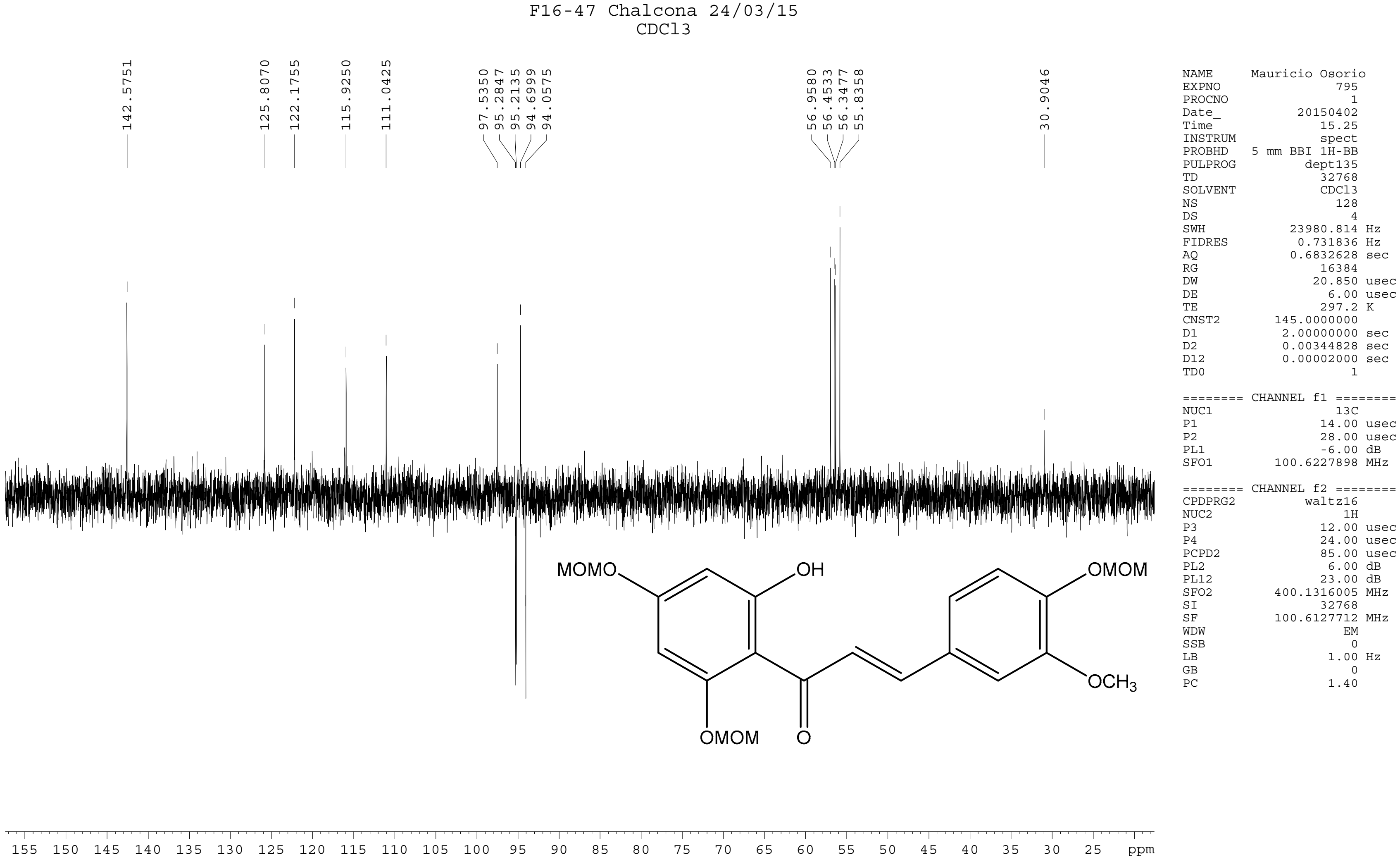

Figure S10. DEPT-135 of **C3.**

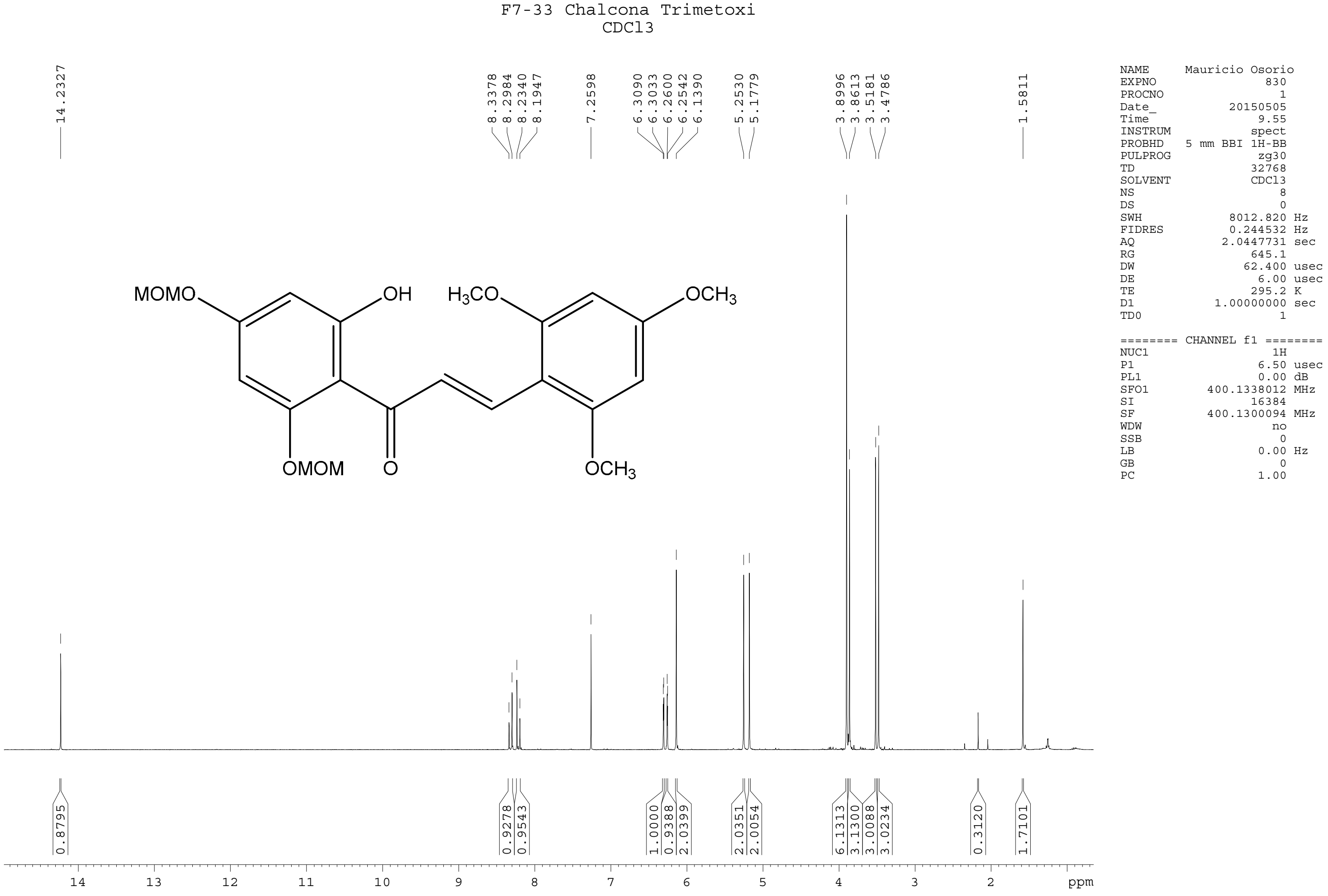

Figure S11. ^{1}H-NMR of **C4** ($CDCl_3$).

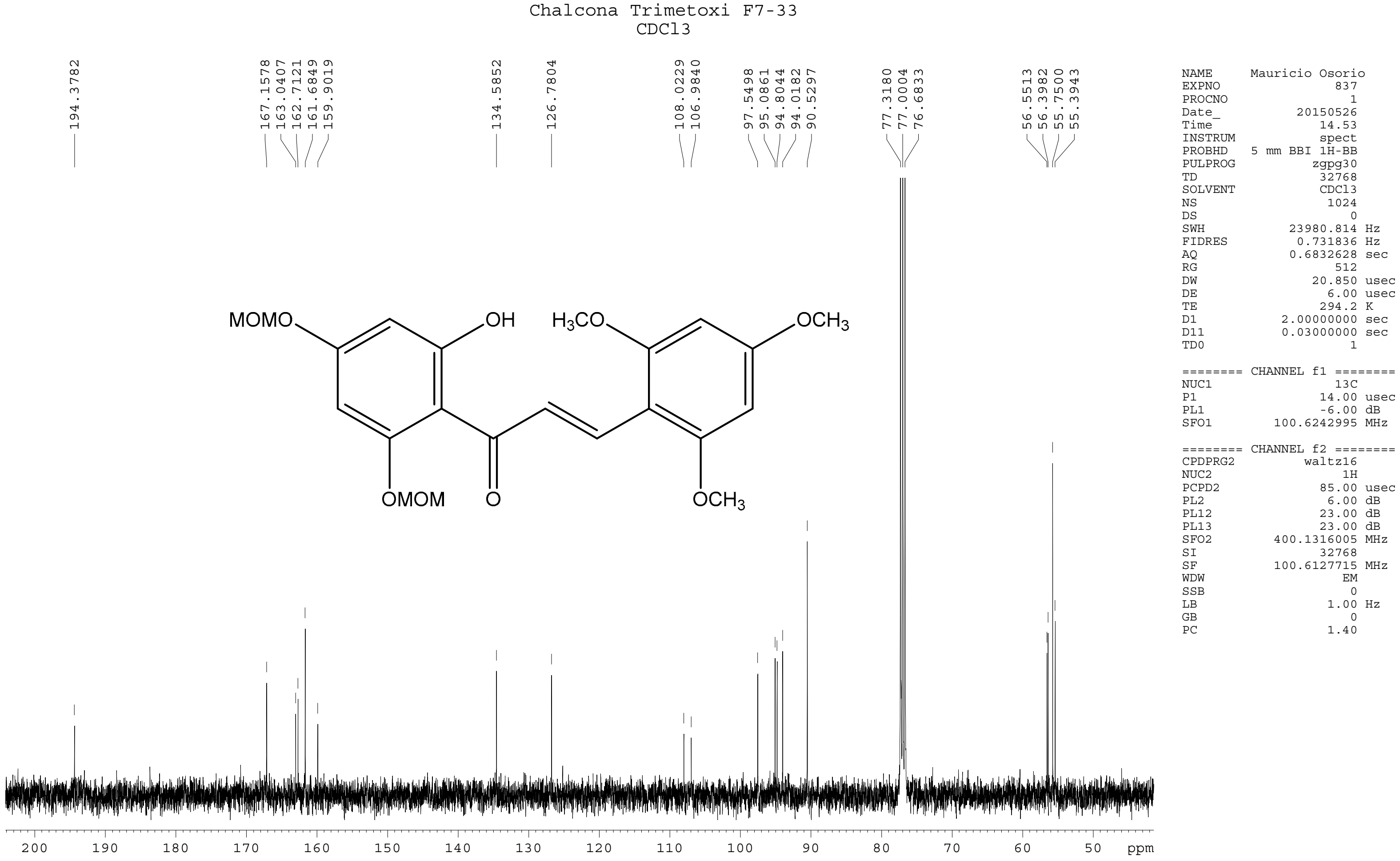

Figure S12. ^{13}C-NMR of **C4.**

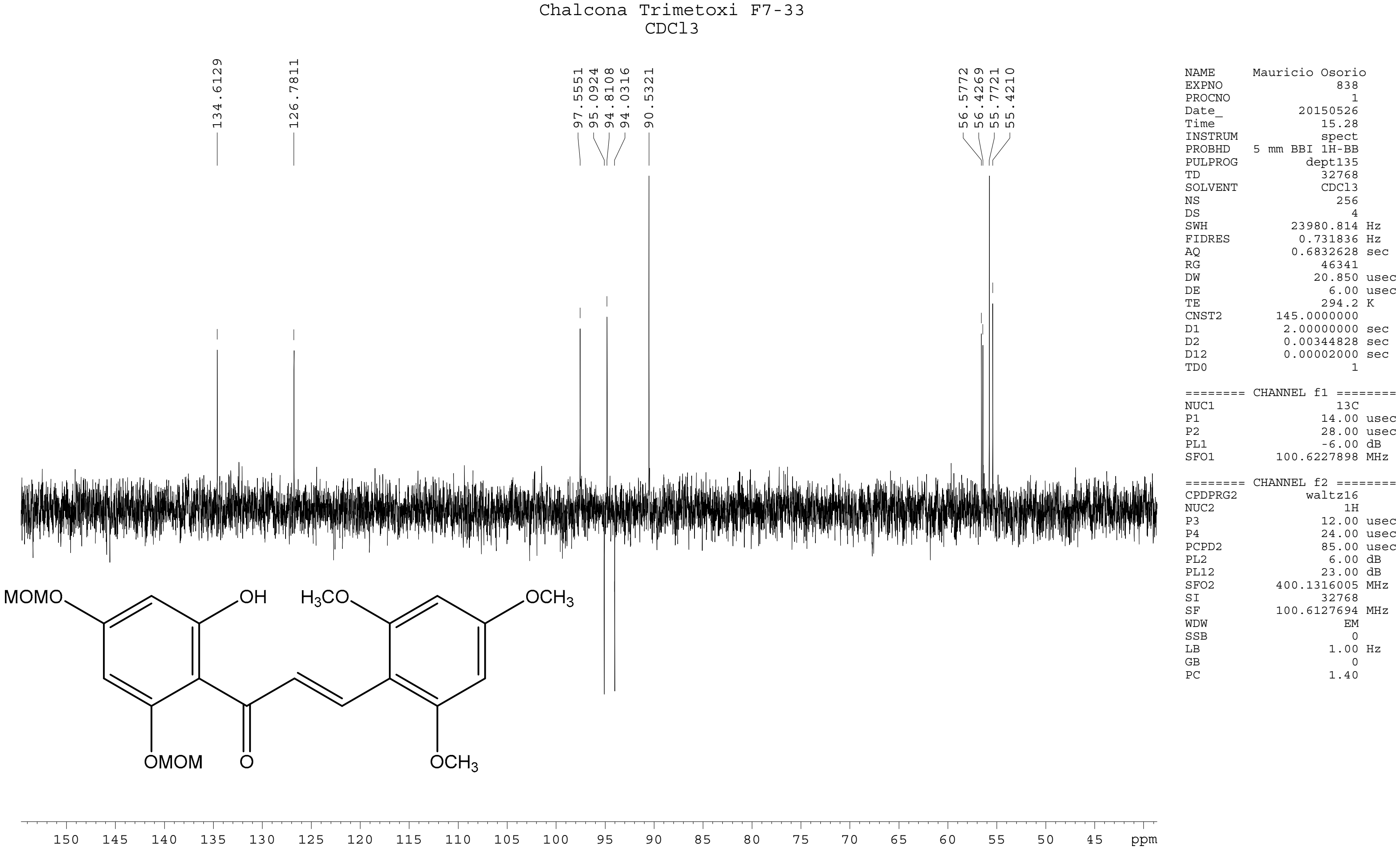

Figure S13. DEPT-135 of **C4.**

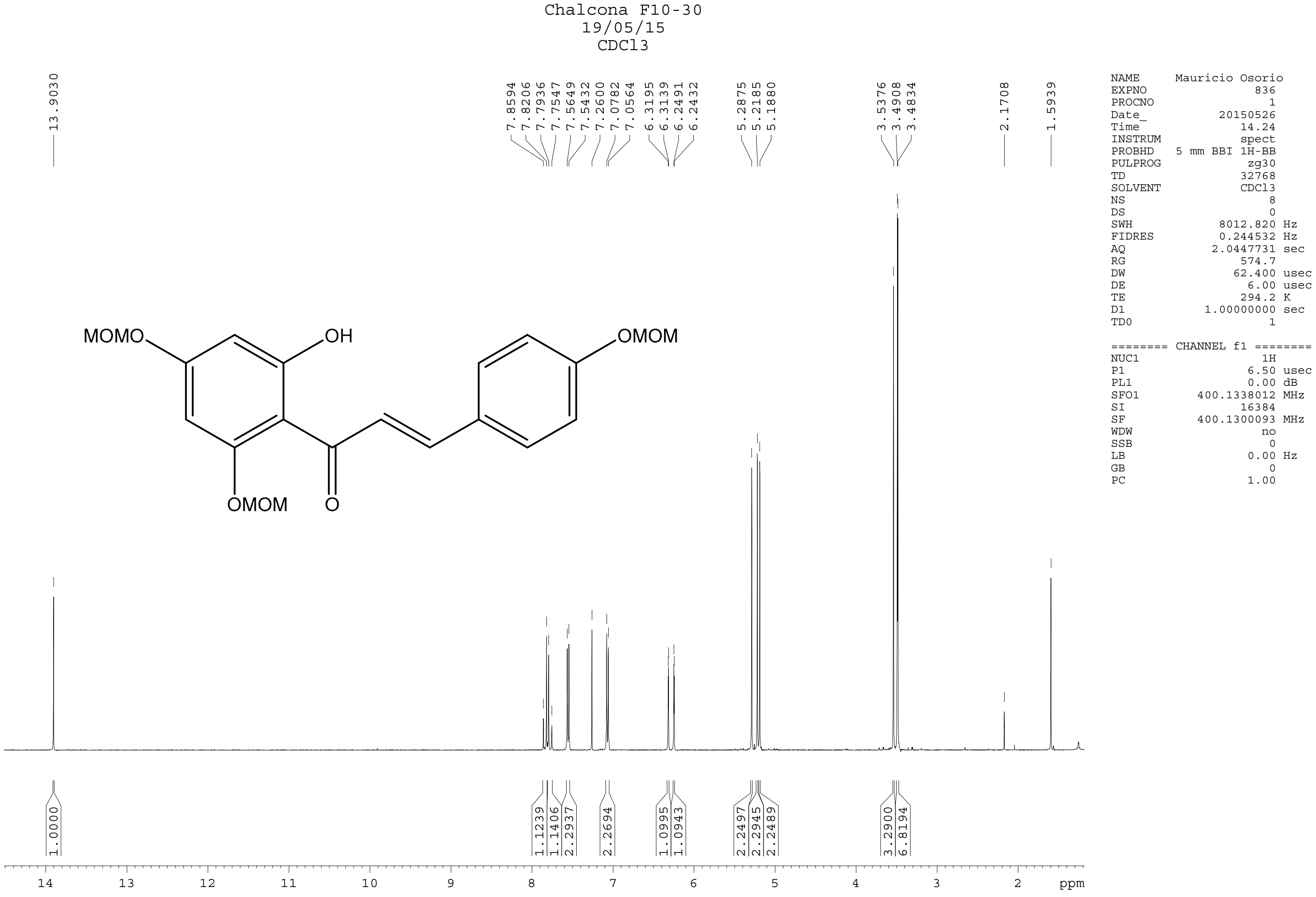

Figure S14. ^{1}H-NMR of **C5** ($CDCl_3$).

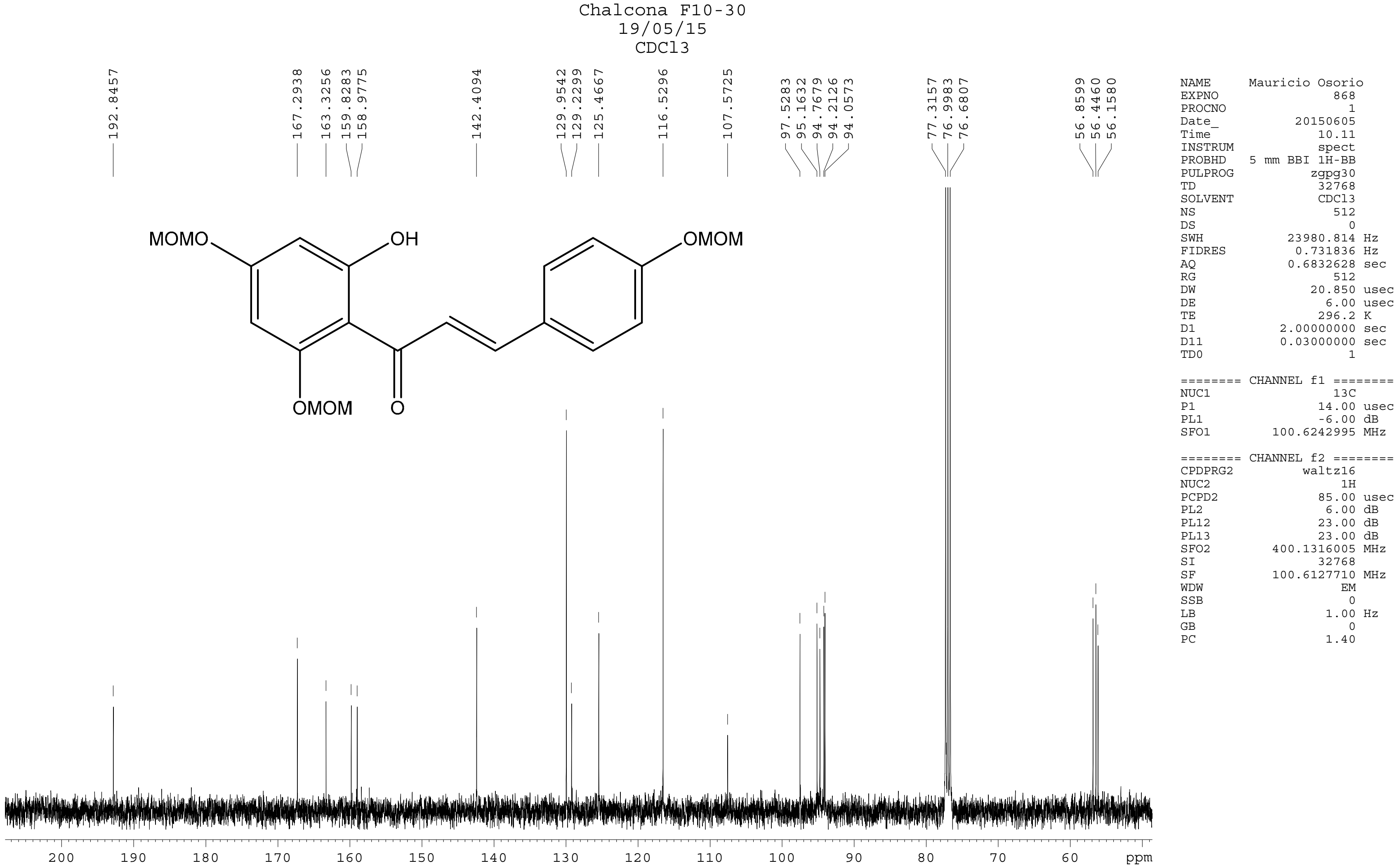

Figure S15. ^{13}C-NMR of **C5.**

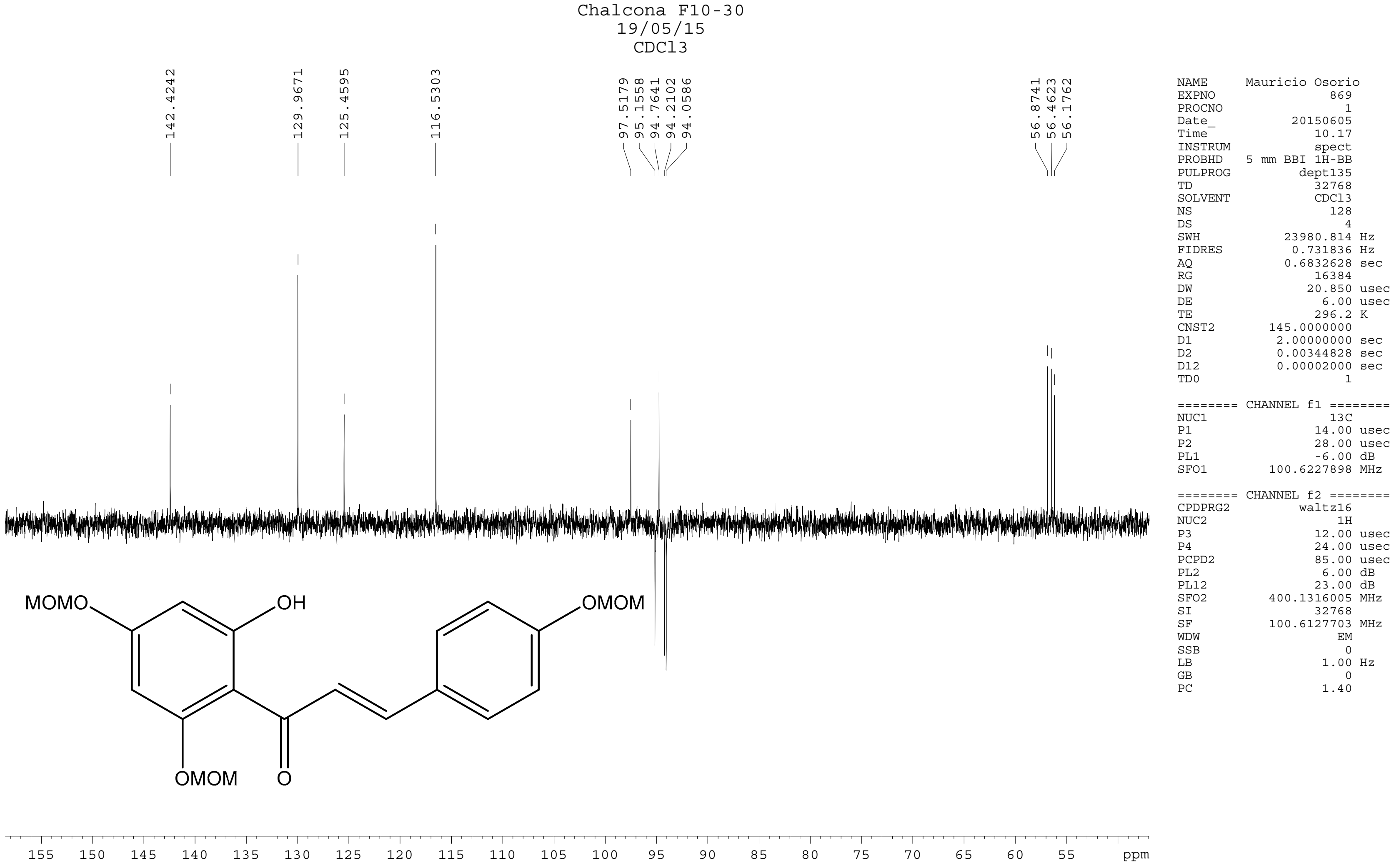

Figure S16. DEPT-135 of **C5.**

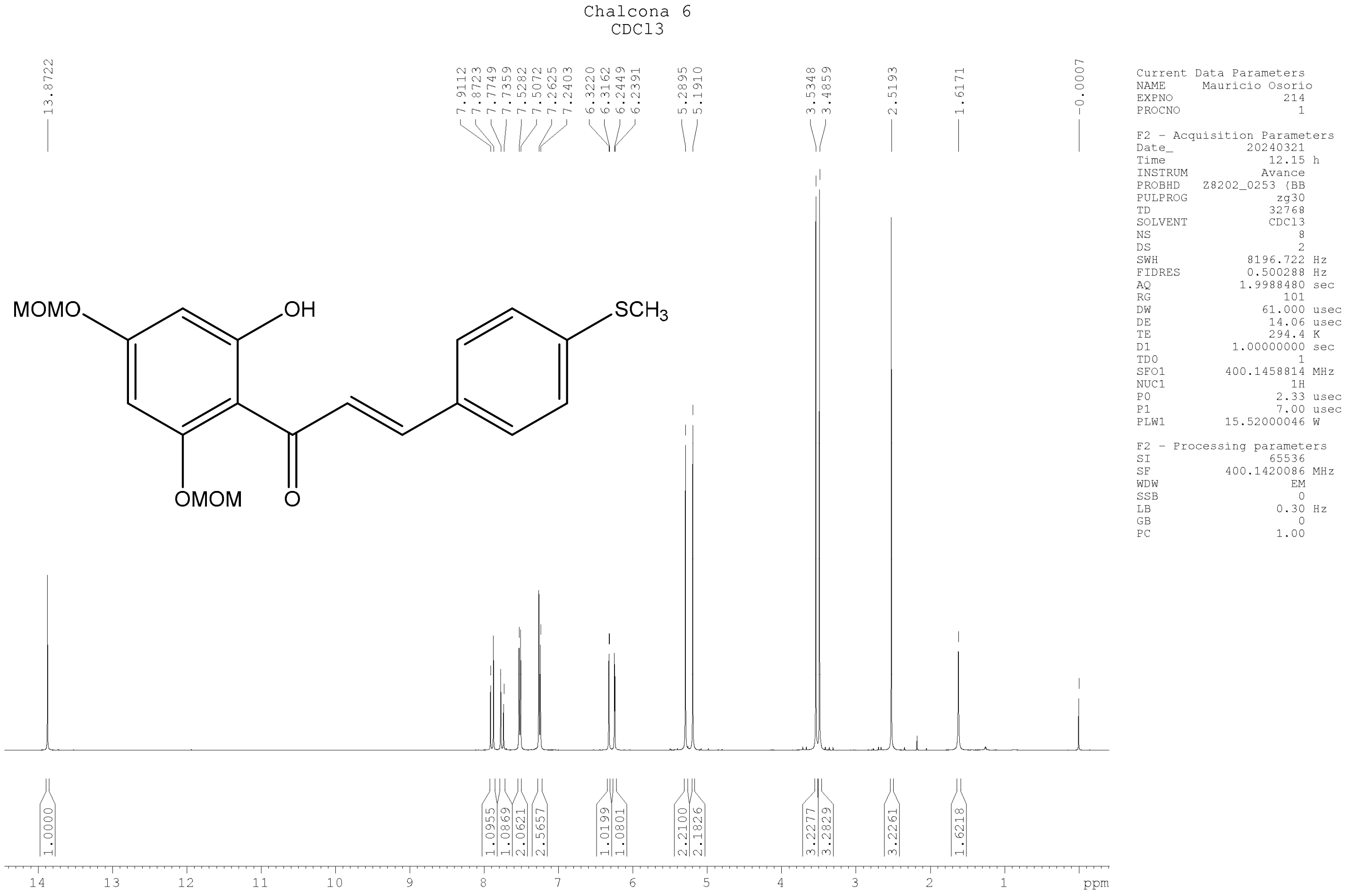

Figure S17. ^{1}H-NMR of **C6** ($CDCl_3$).

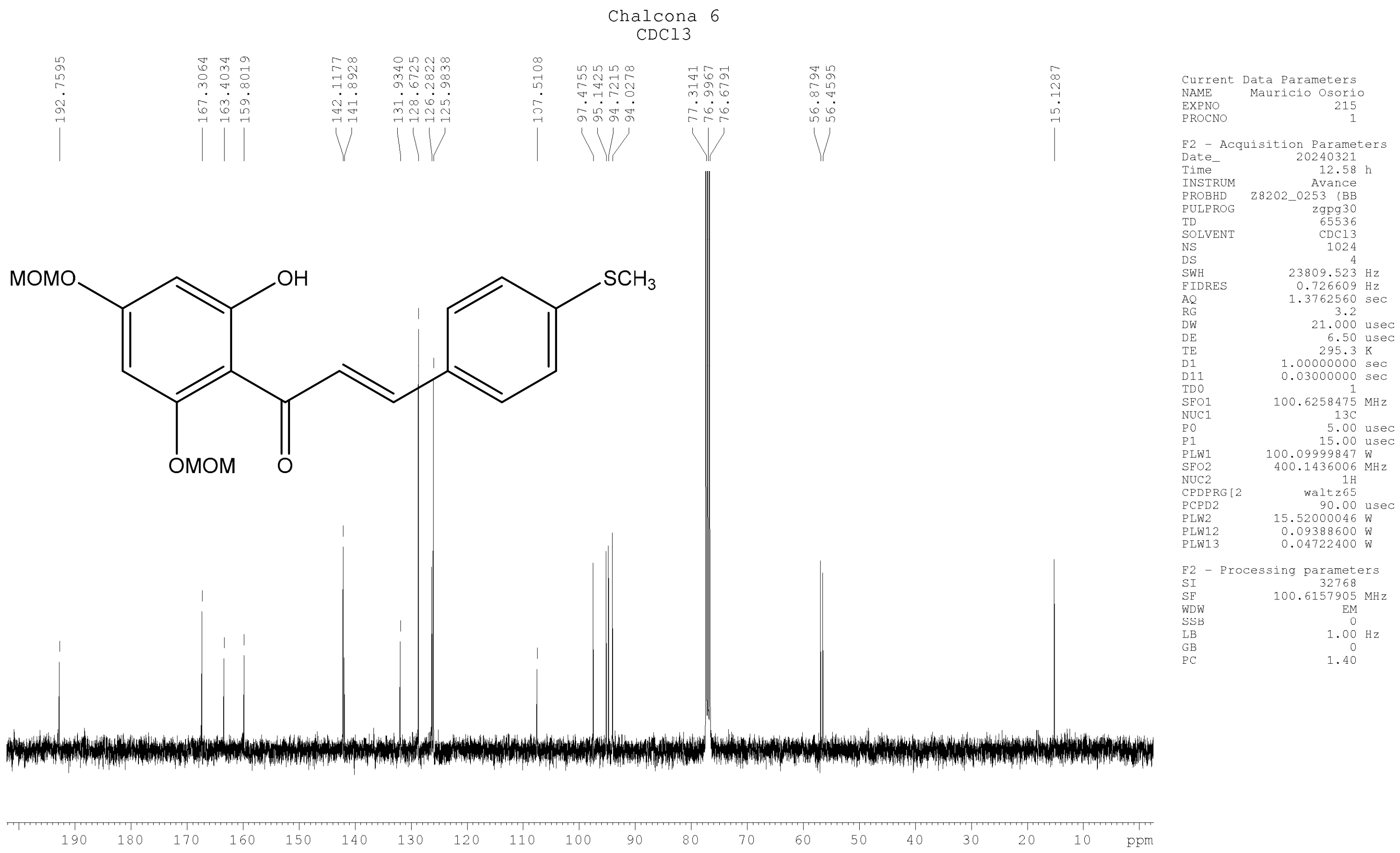

Figure S18. ^{13}C-NMR of **C6.**

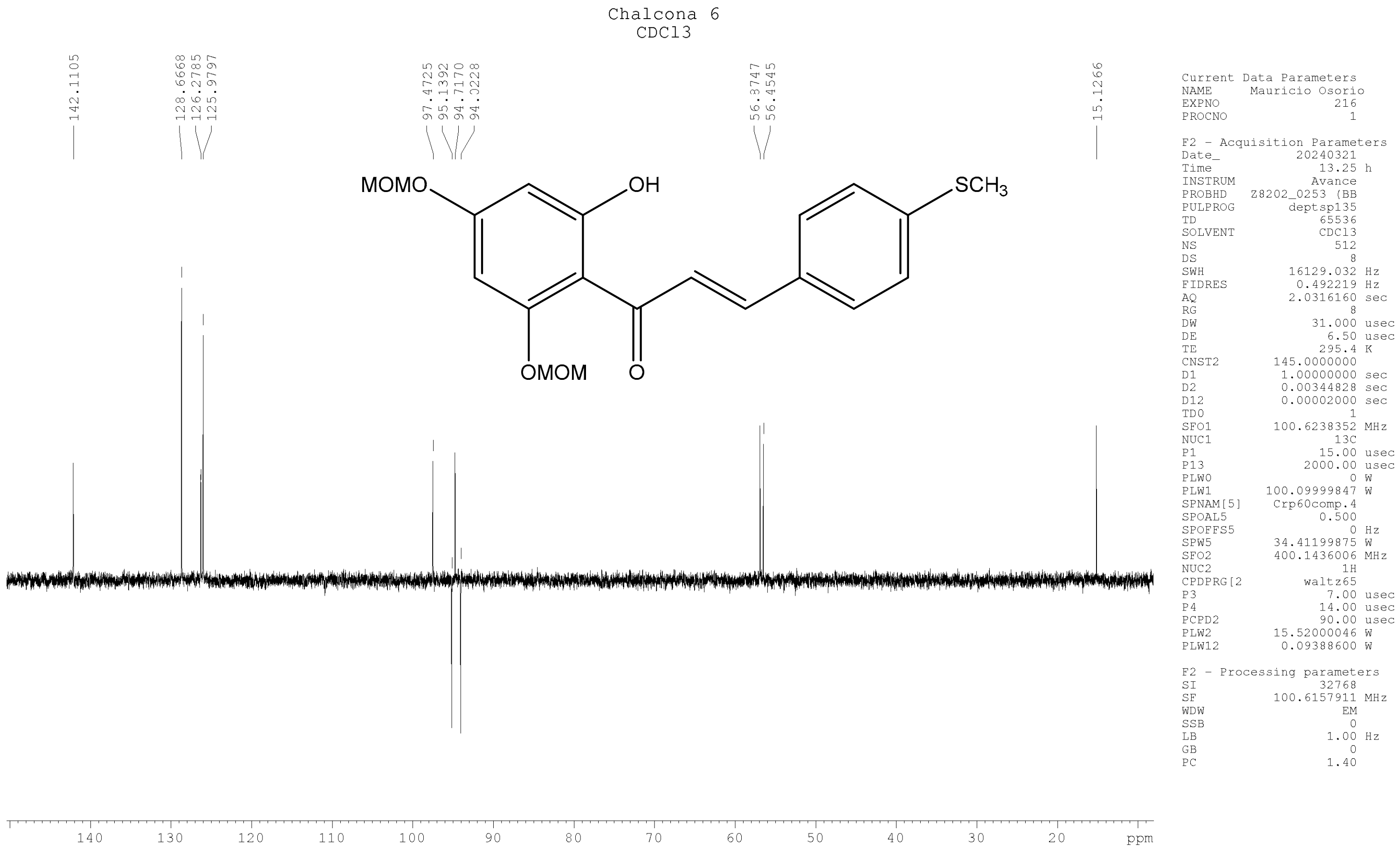

Figure S19. DEPT-135 of **C6.**

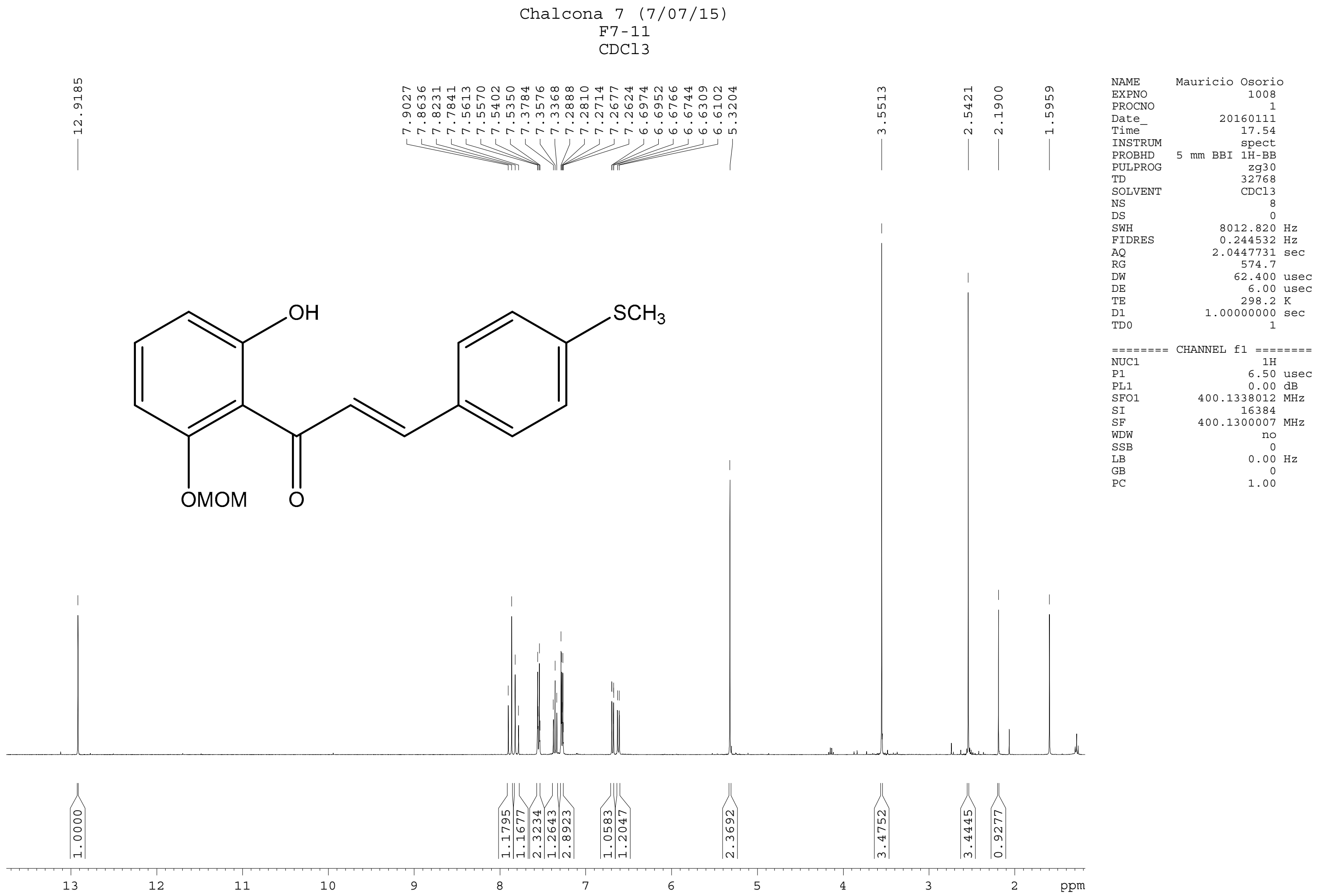

Figure S20. ^{1}H-NMR of **C7** ($CDCl_3$).

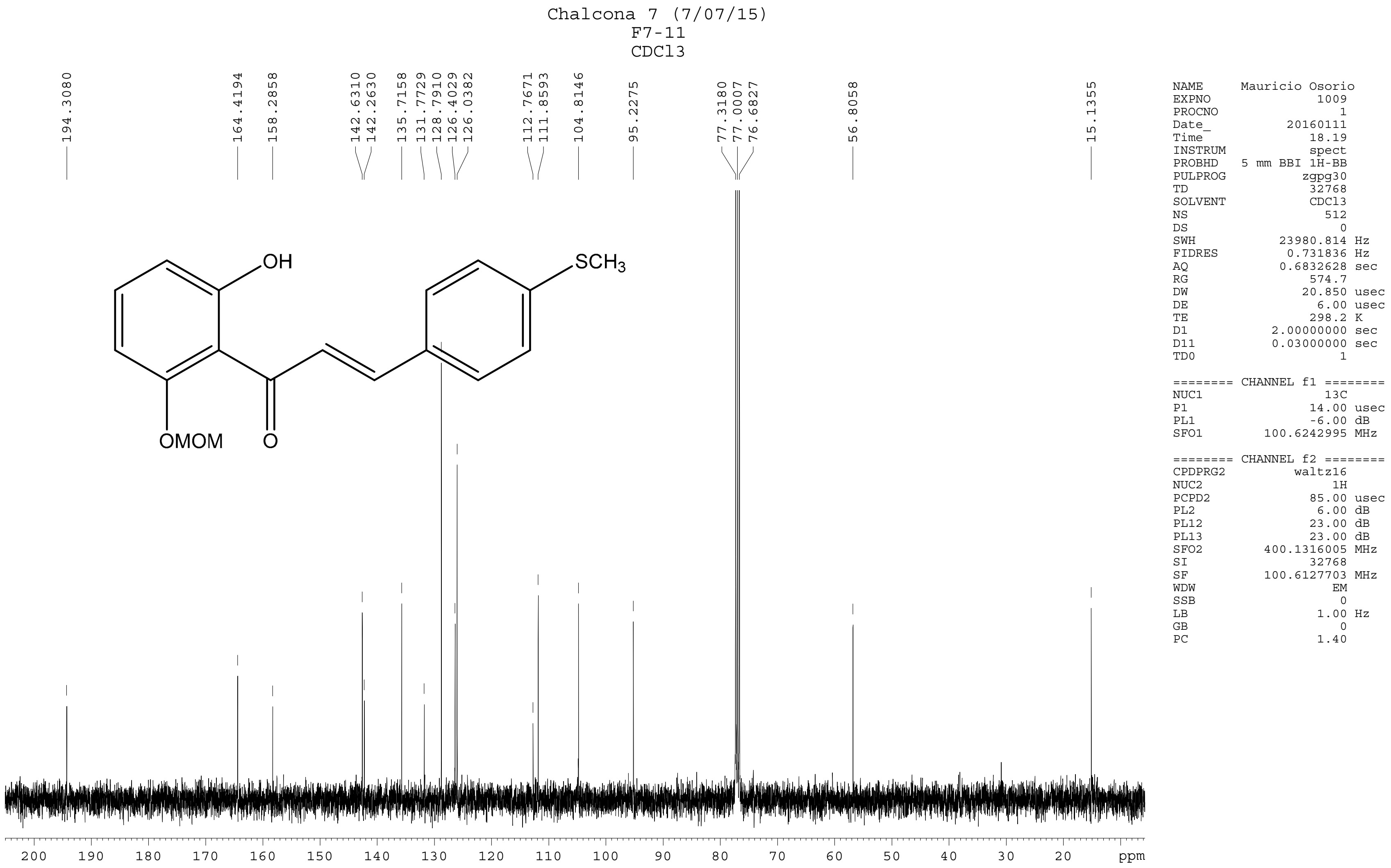

Figure S21. ^{13}C-NMR of **C7.**

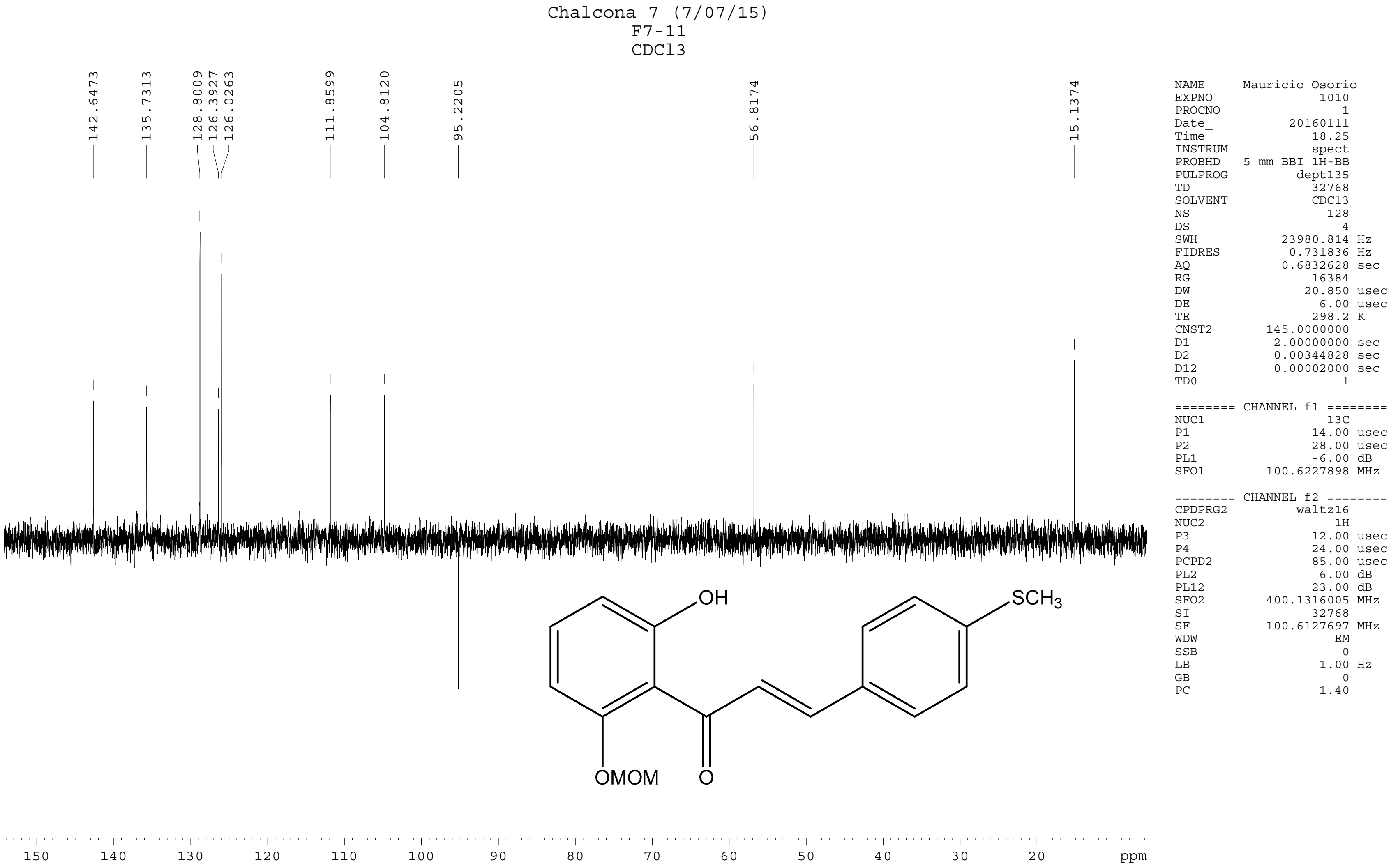

Figure S22. DEPT-135 of **C7.**

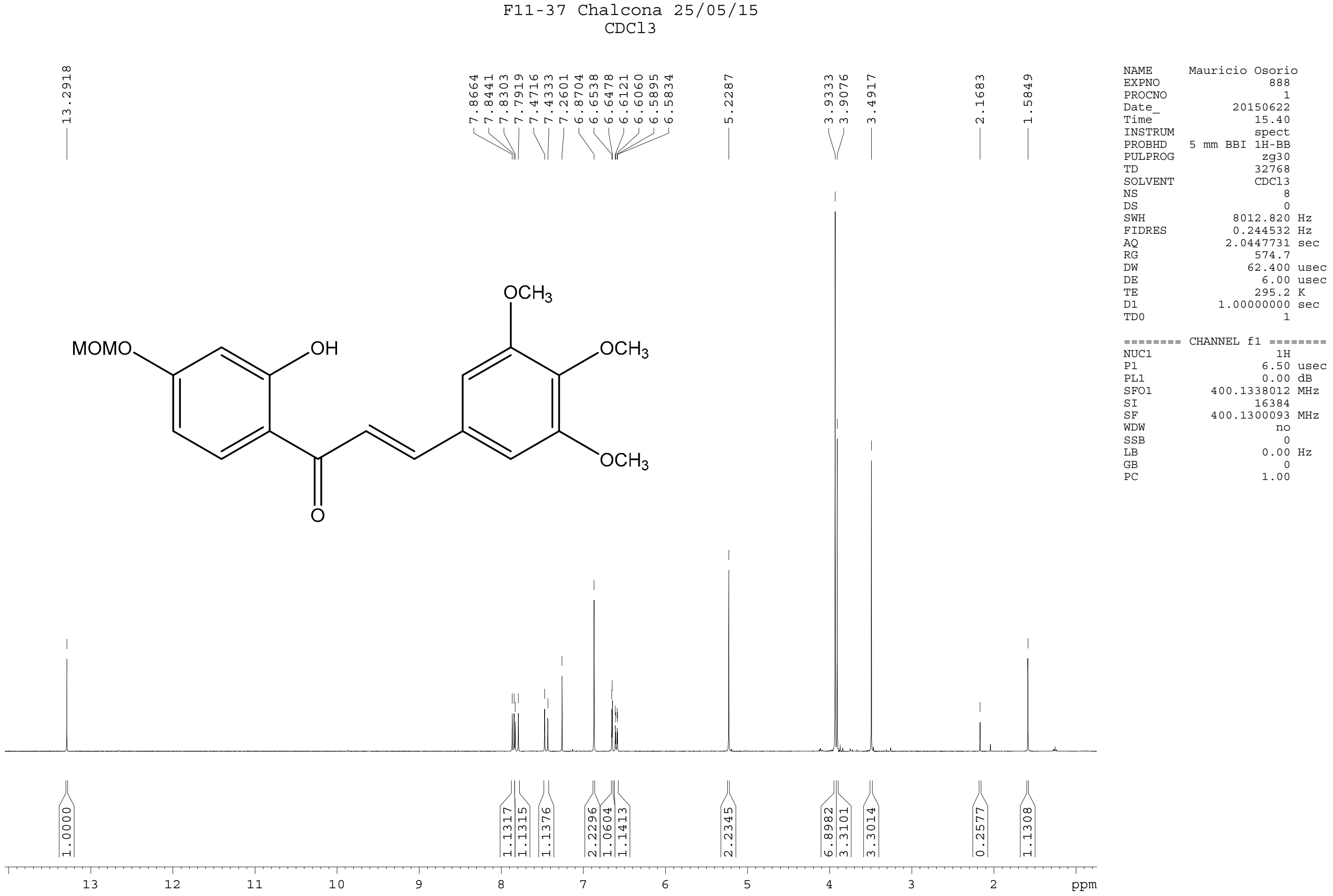

Figure S23. ^{1}H-NMR of **C8** ($CDCl_3$).

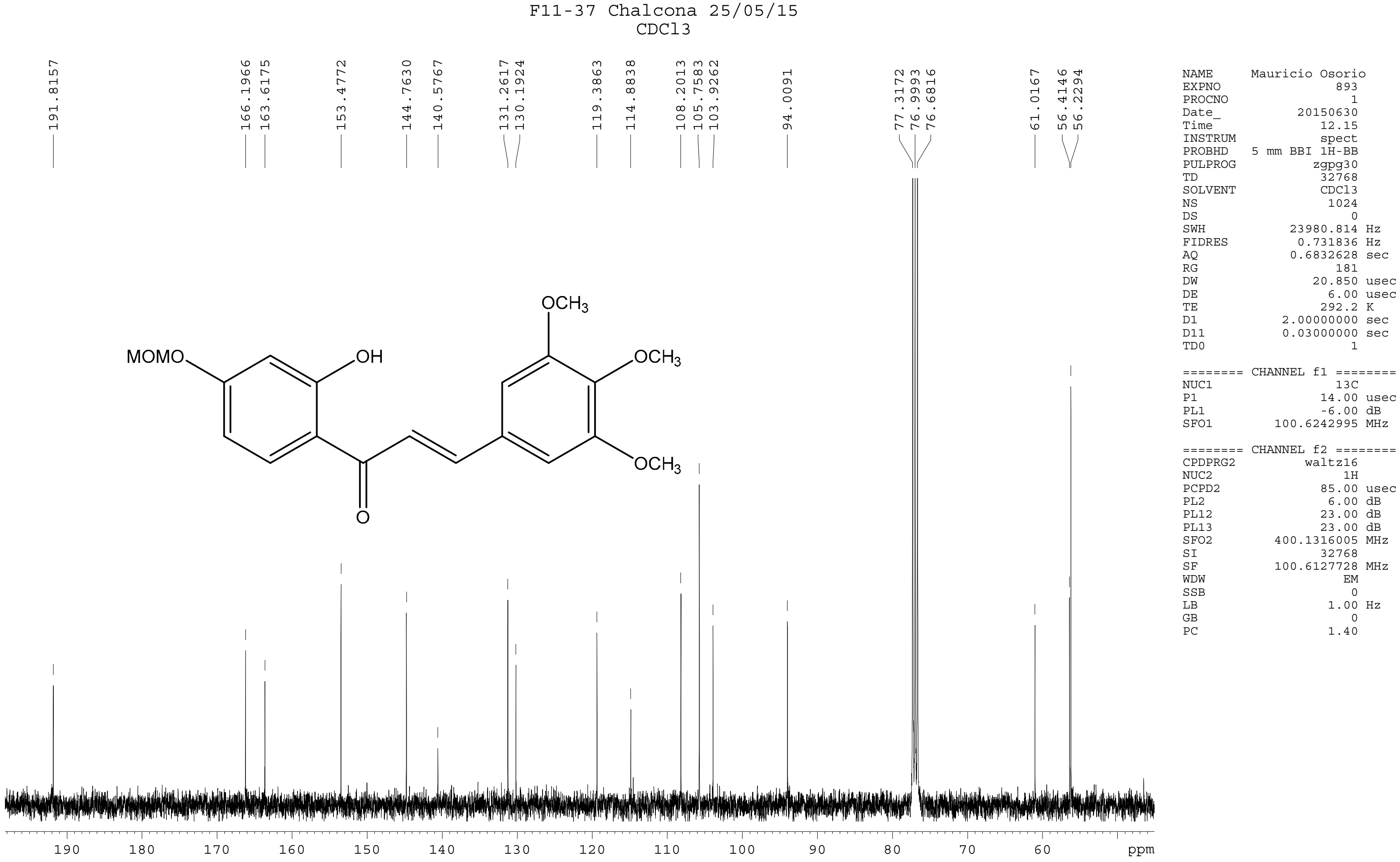

Figure S24. ^{13}C-NMR of **C8.**

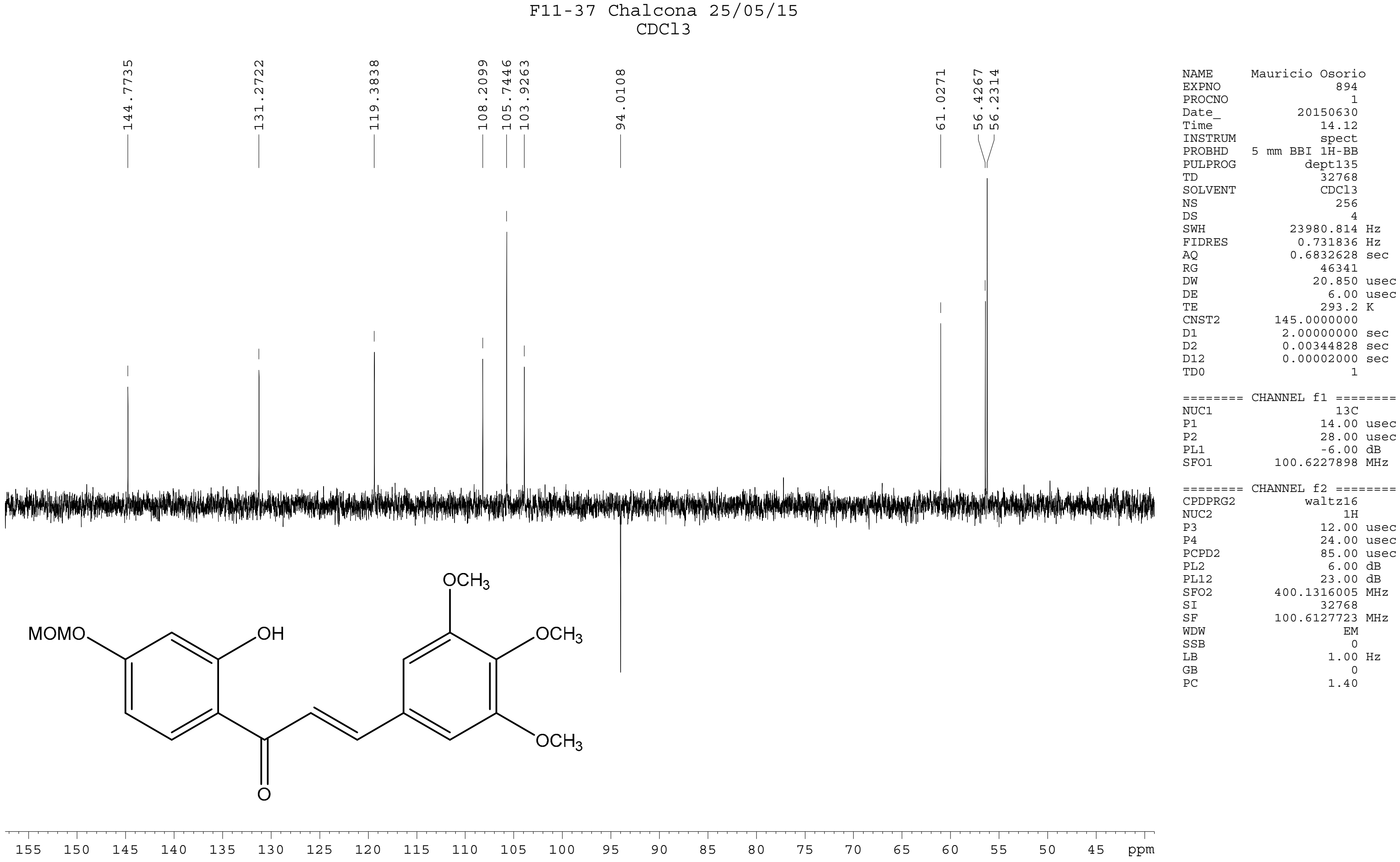

Figure S25. DEPT-135 of **C8.**

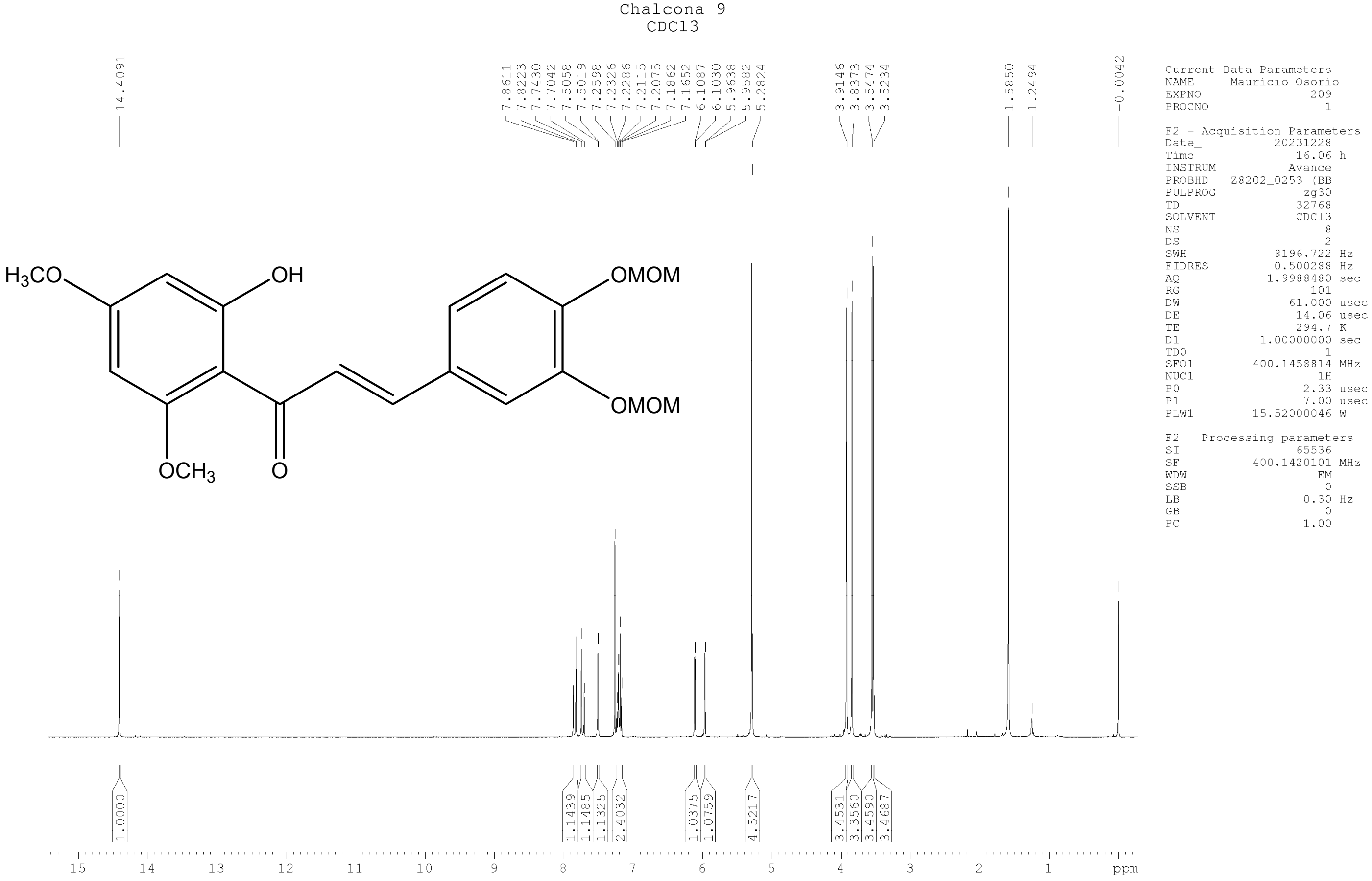

Figure S26. ^{1}H-NMR of **C9** ($CDCl_3$).

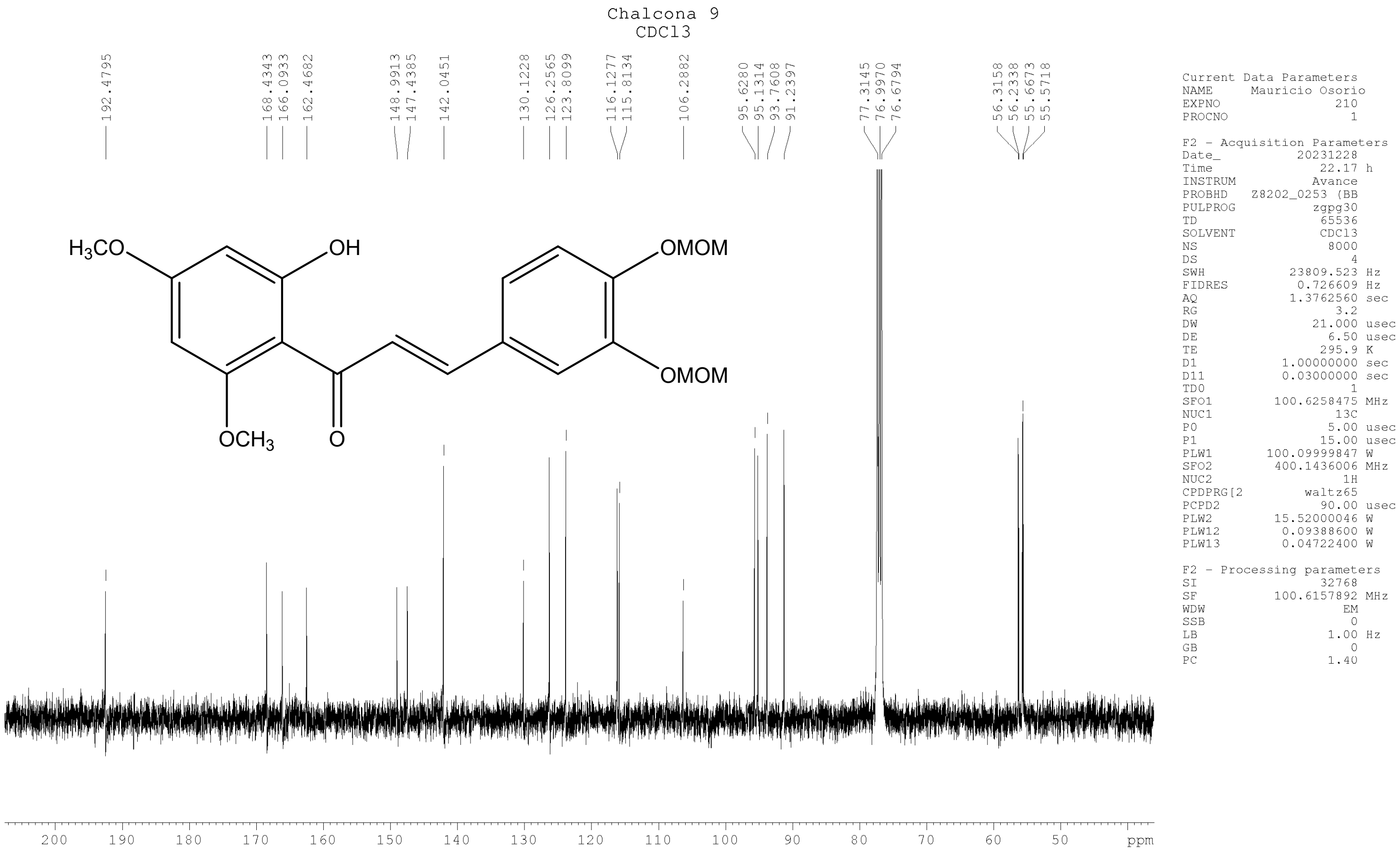

Figure S27. ^{13}C-NMR of **C9.**

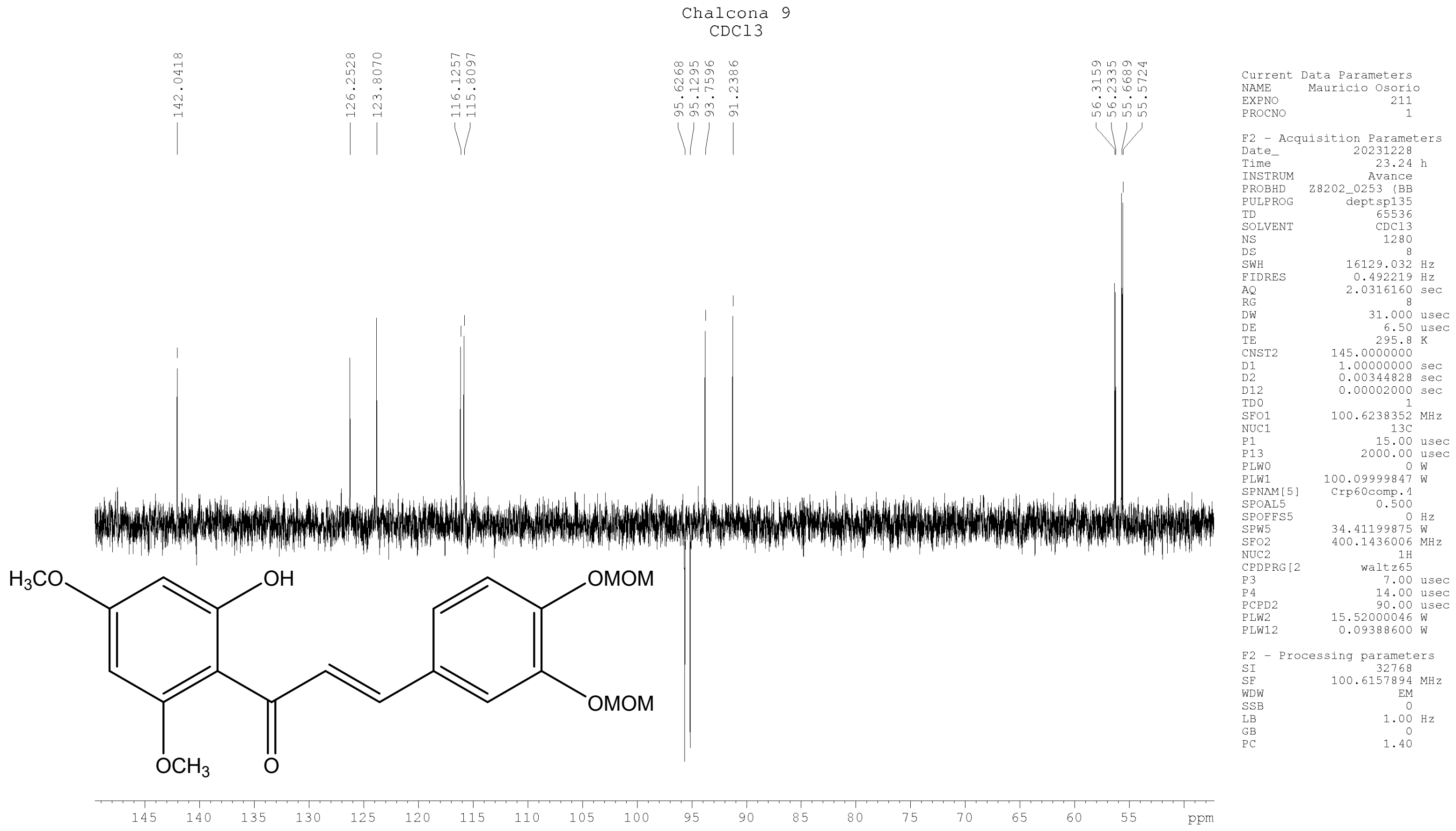

Figure S28. DEPT-135 of **C9.**

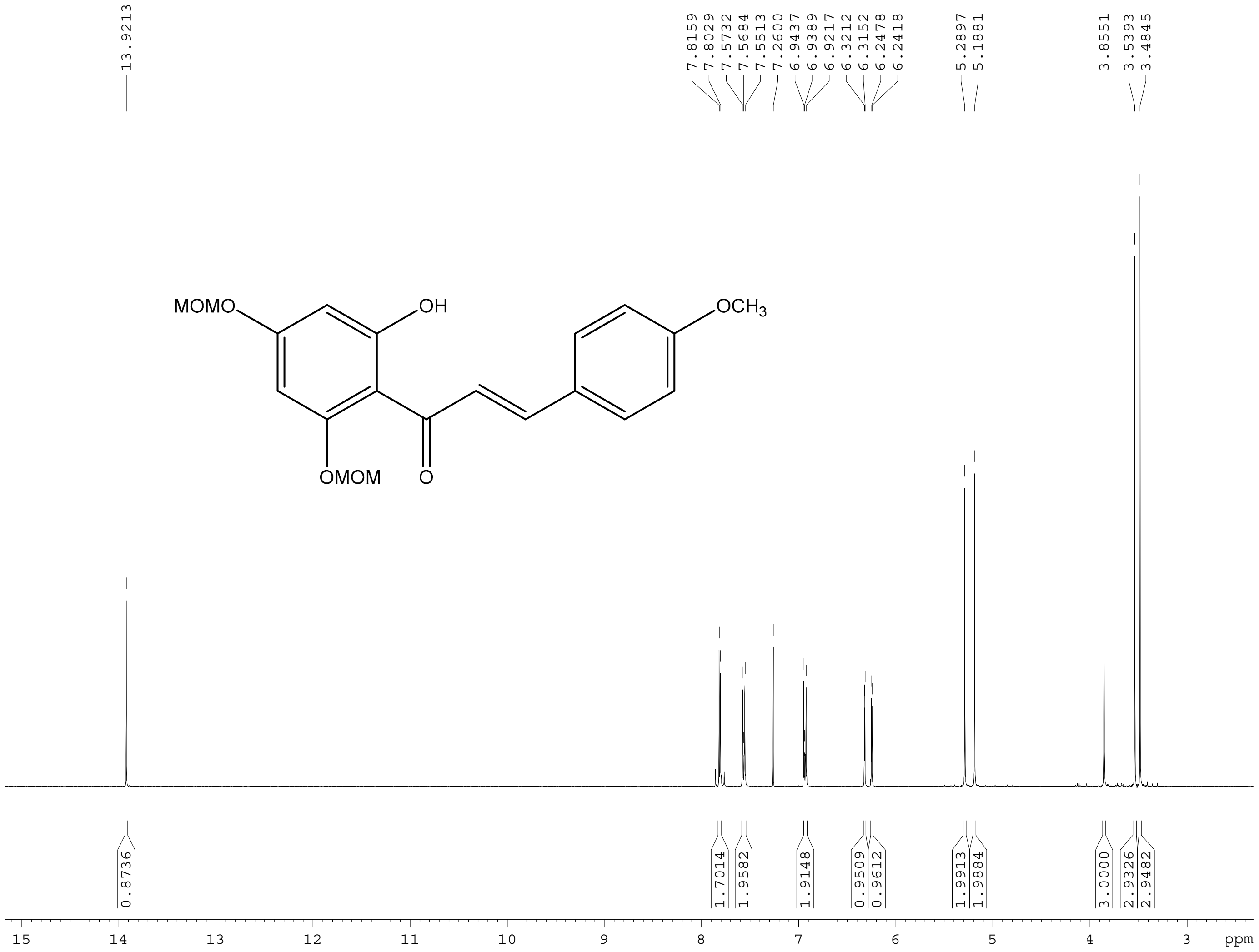

Figure S29. ^{1}H-NMR of **C10** ($CDCl_3$).

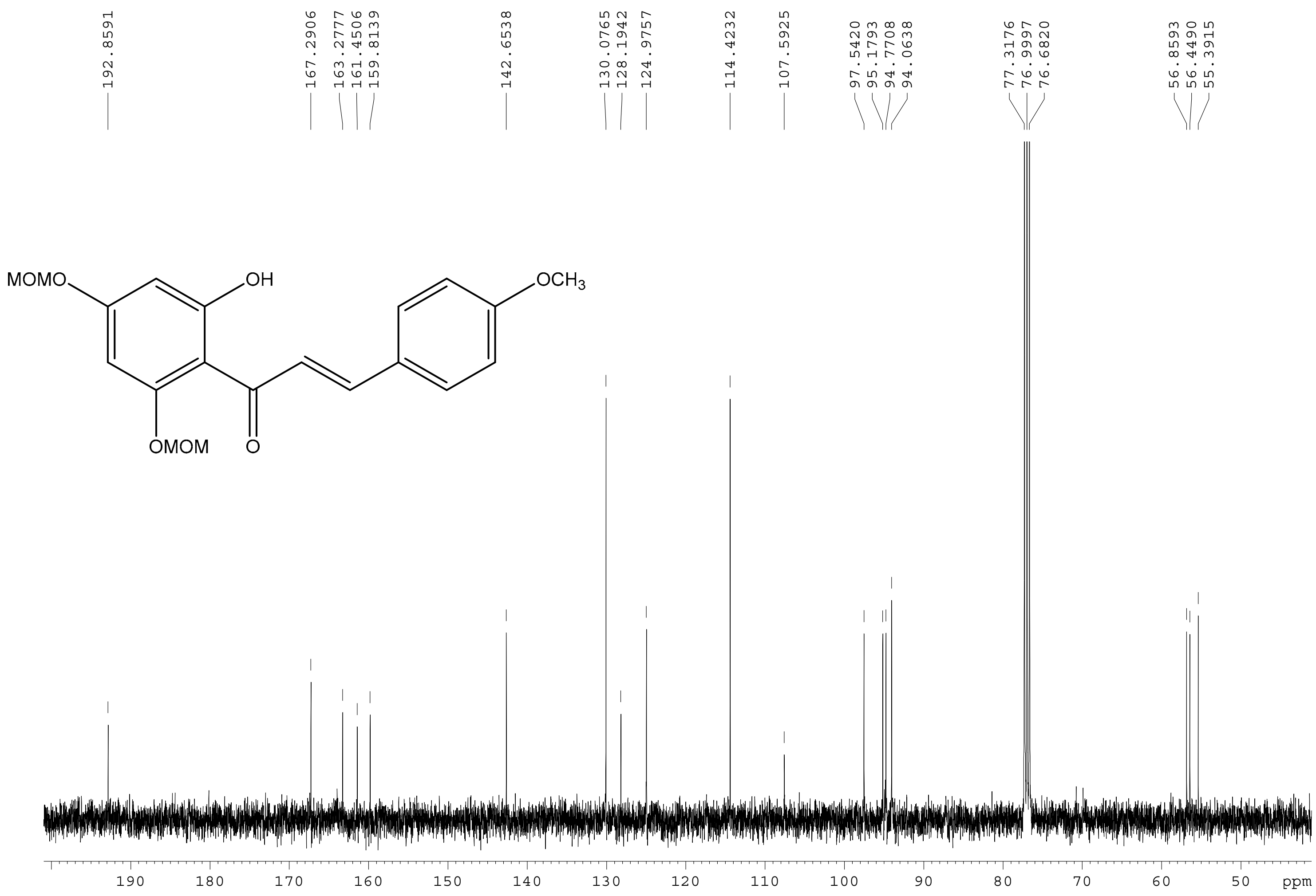

Figure S30. ^{13}C-NMR of **C10.**

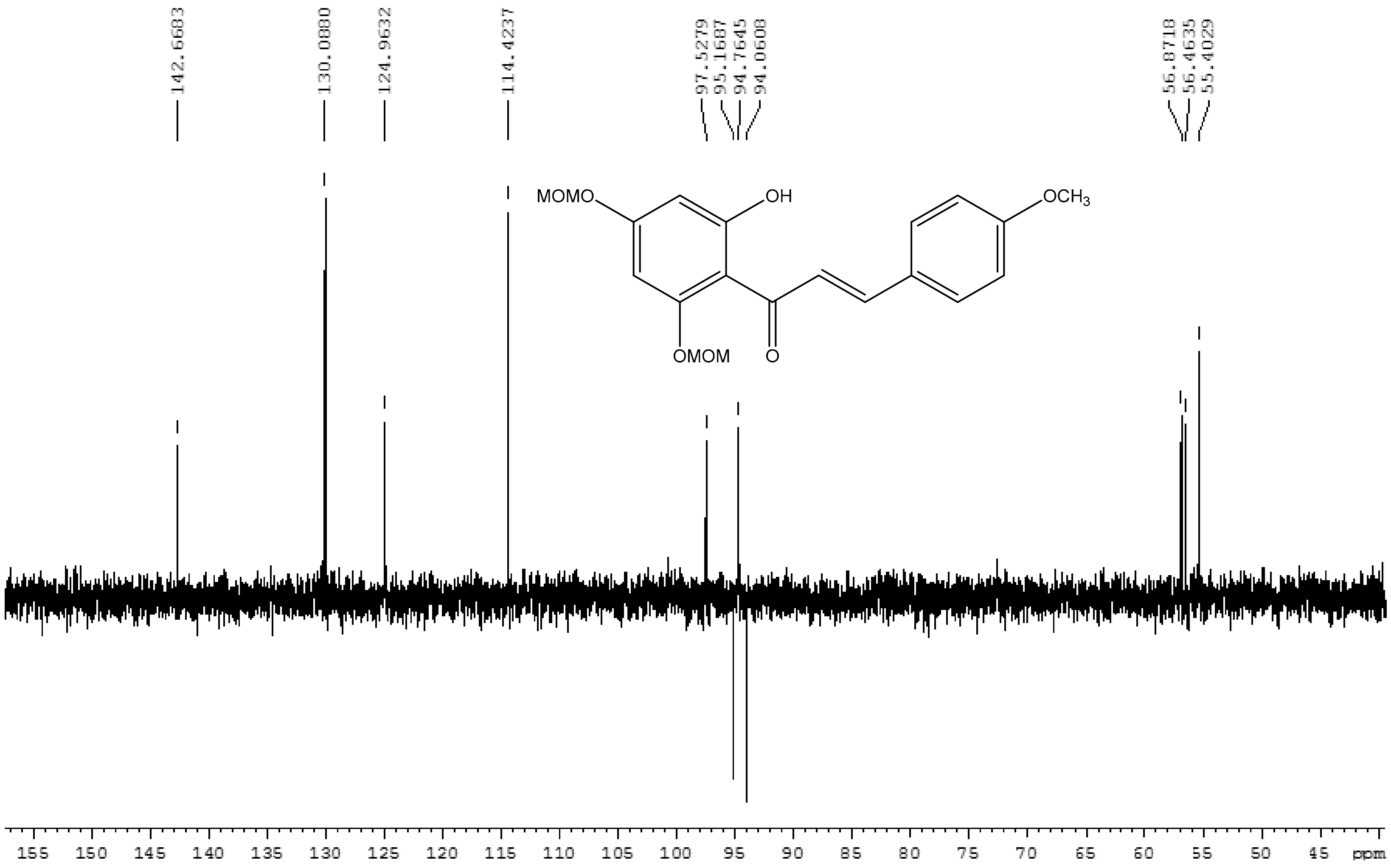

Figure S31. DEPT-135 of **C10.**

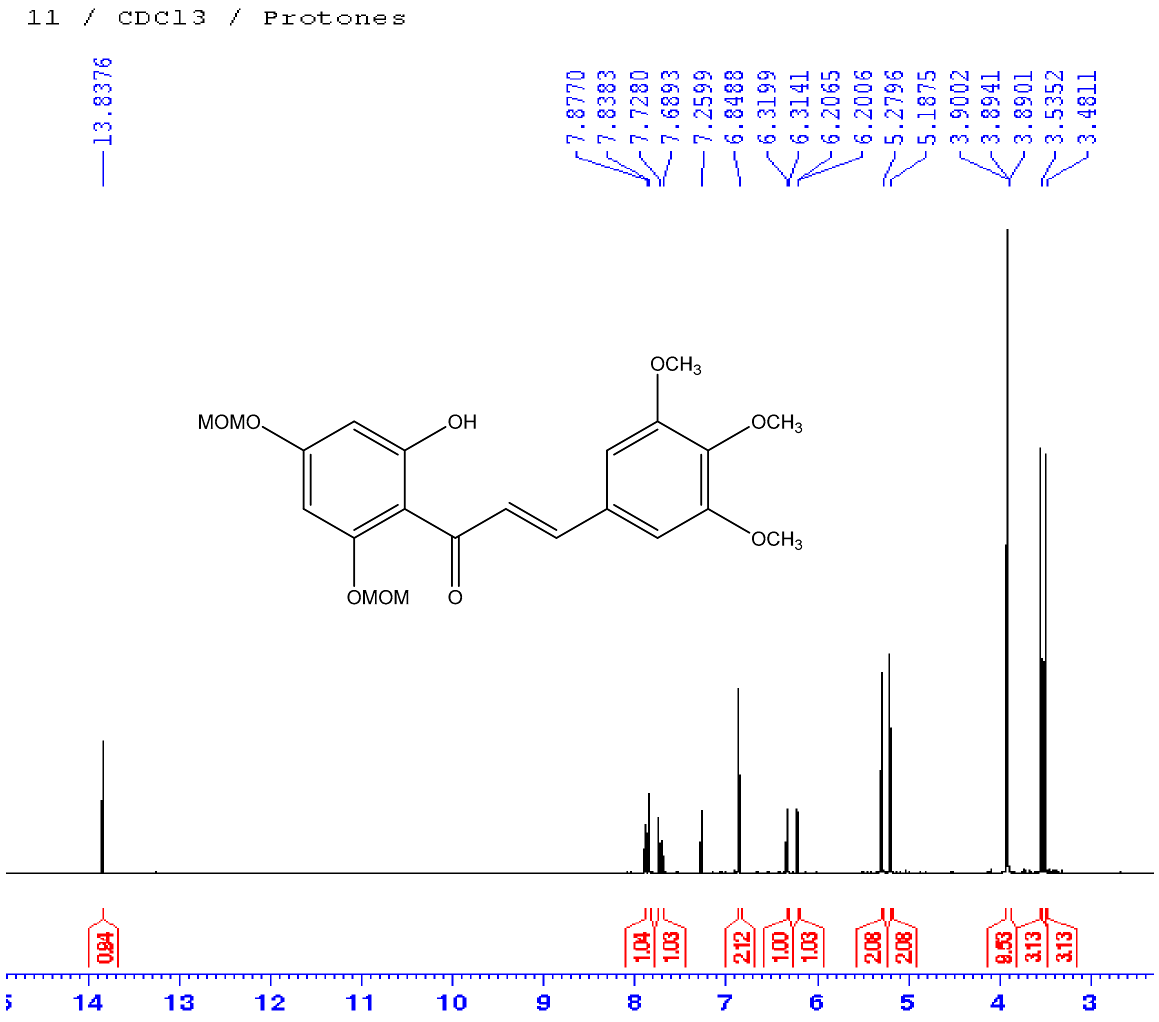

Figure S32. ^{1}H-NMR of **C11** ($CDCl_3$).

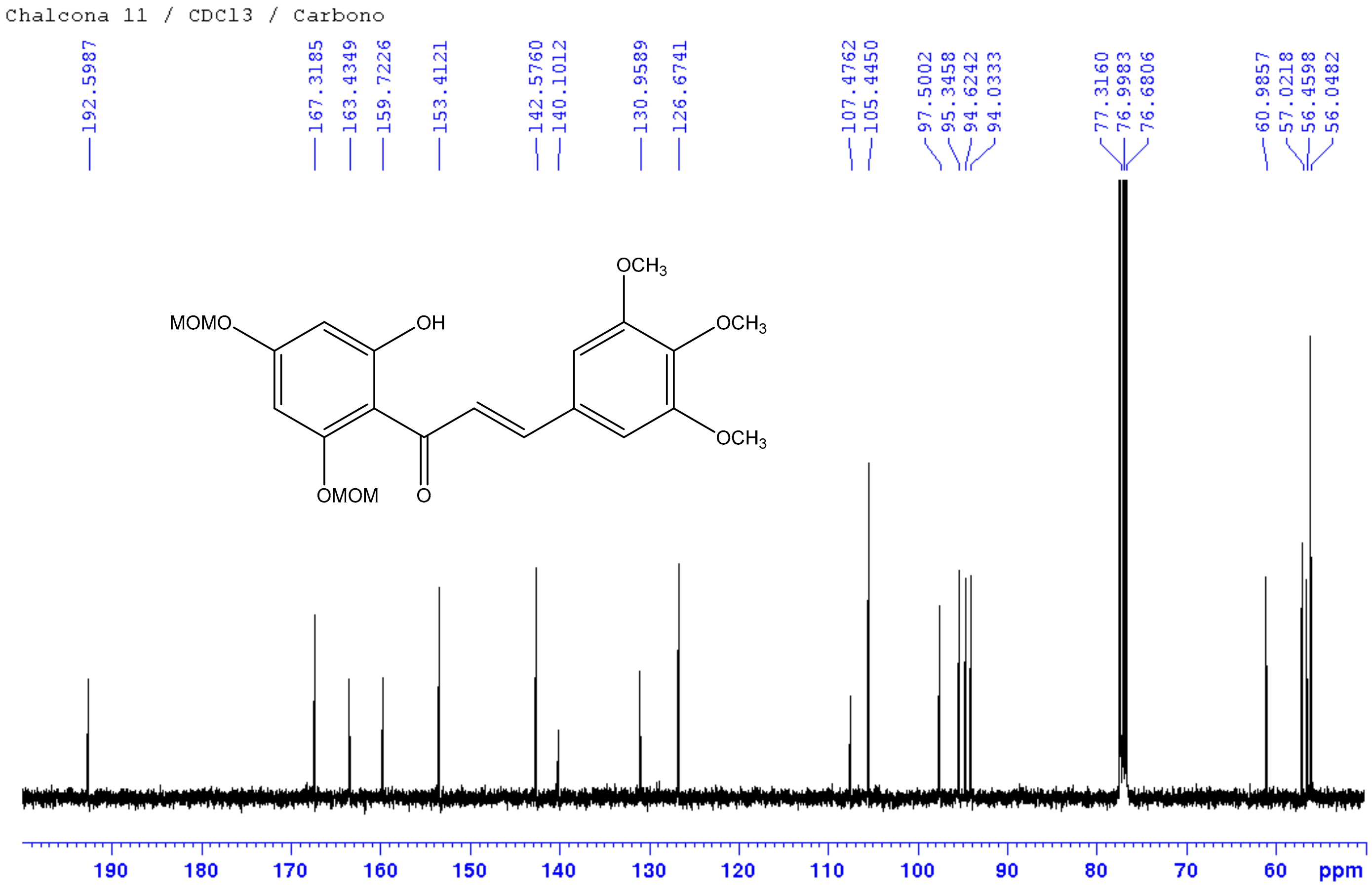

Figure S33. ^{13}C-NMR of **C11.**

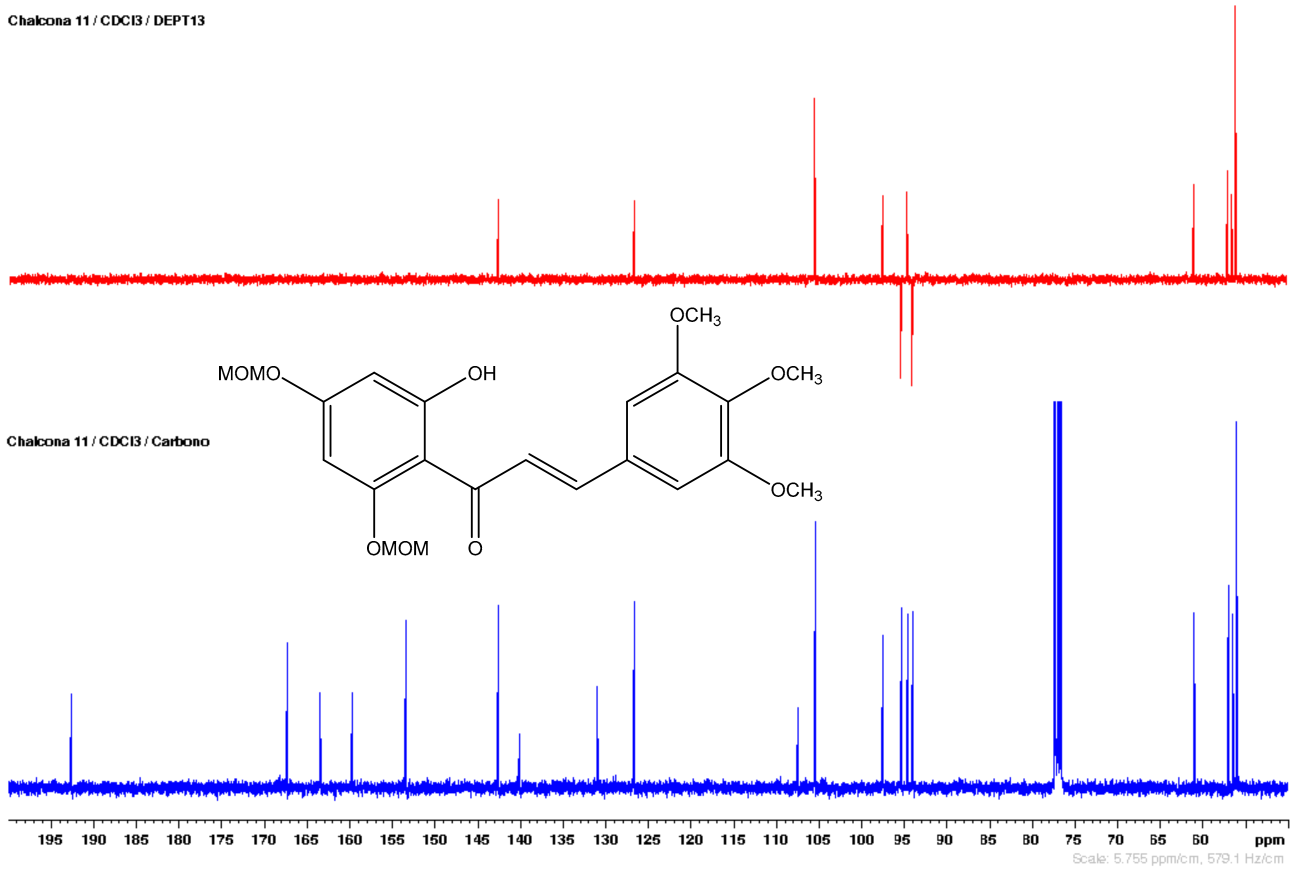

Figure S34. DEPT-135 of **C11.**

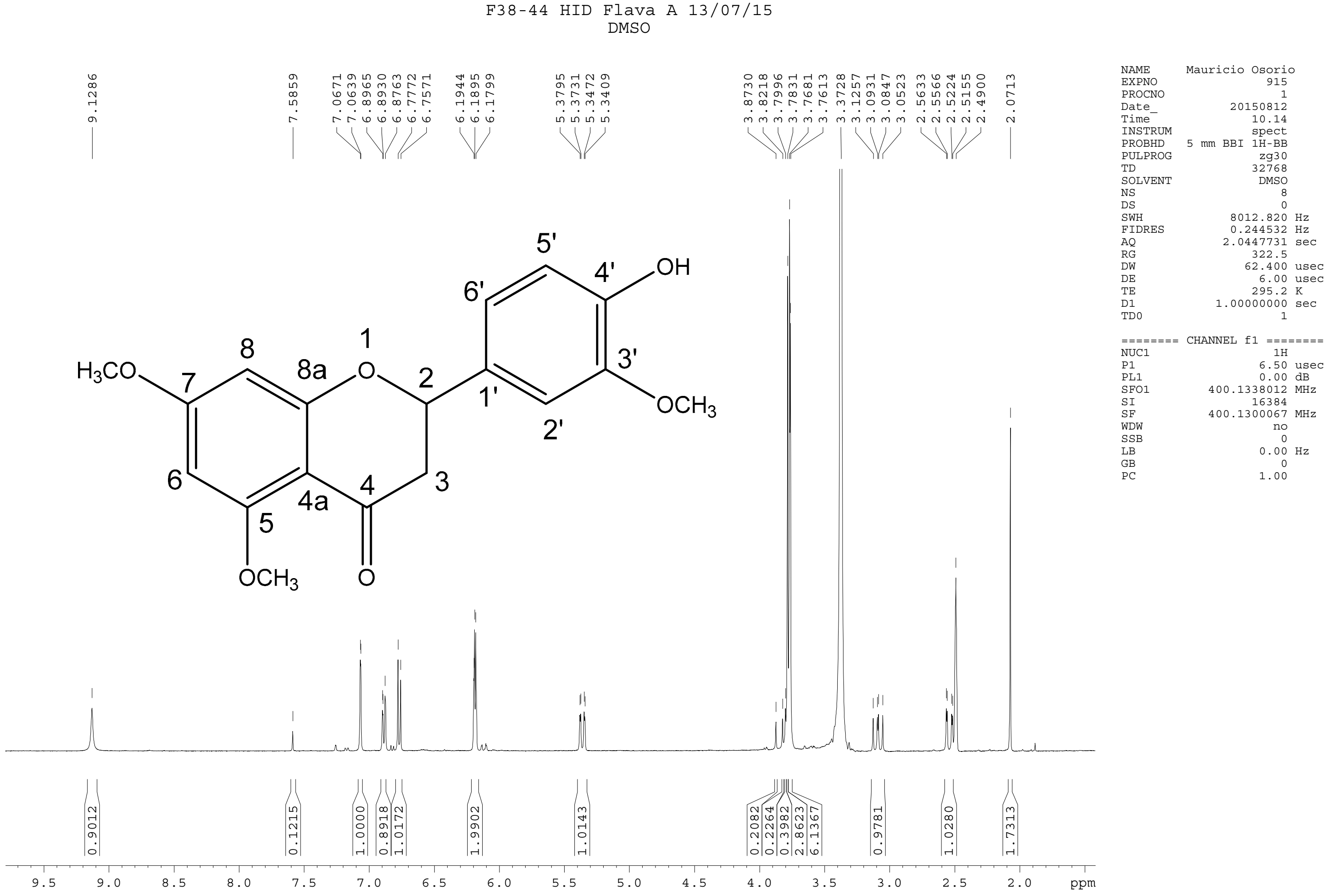

Figure S35. ^{1}H-NMR of **FV1** (DMSO-*d*6).

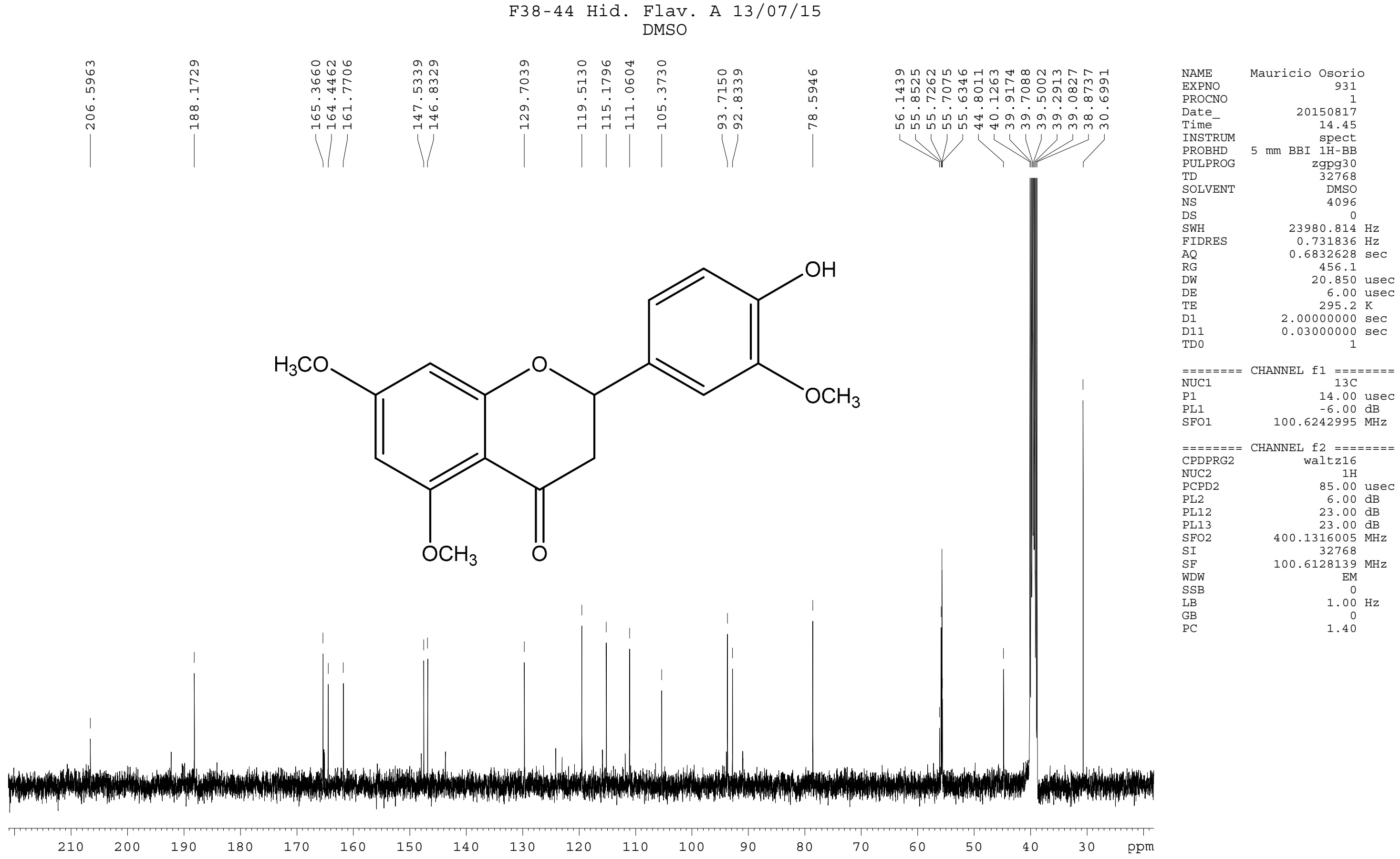

Figure S36. ^{13}C-NMR of **FV1** (DMSO-d_6).

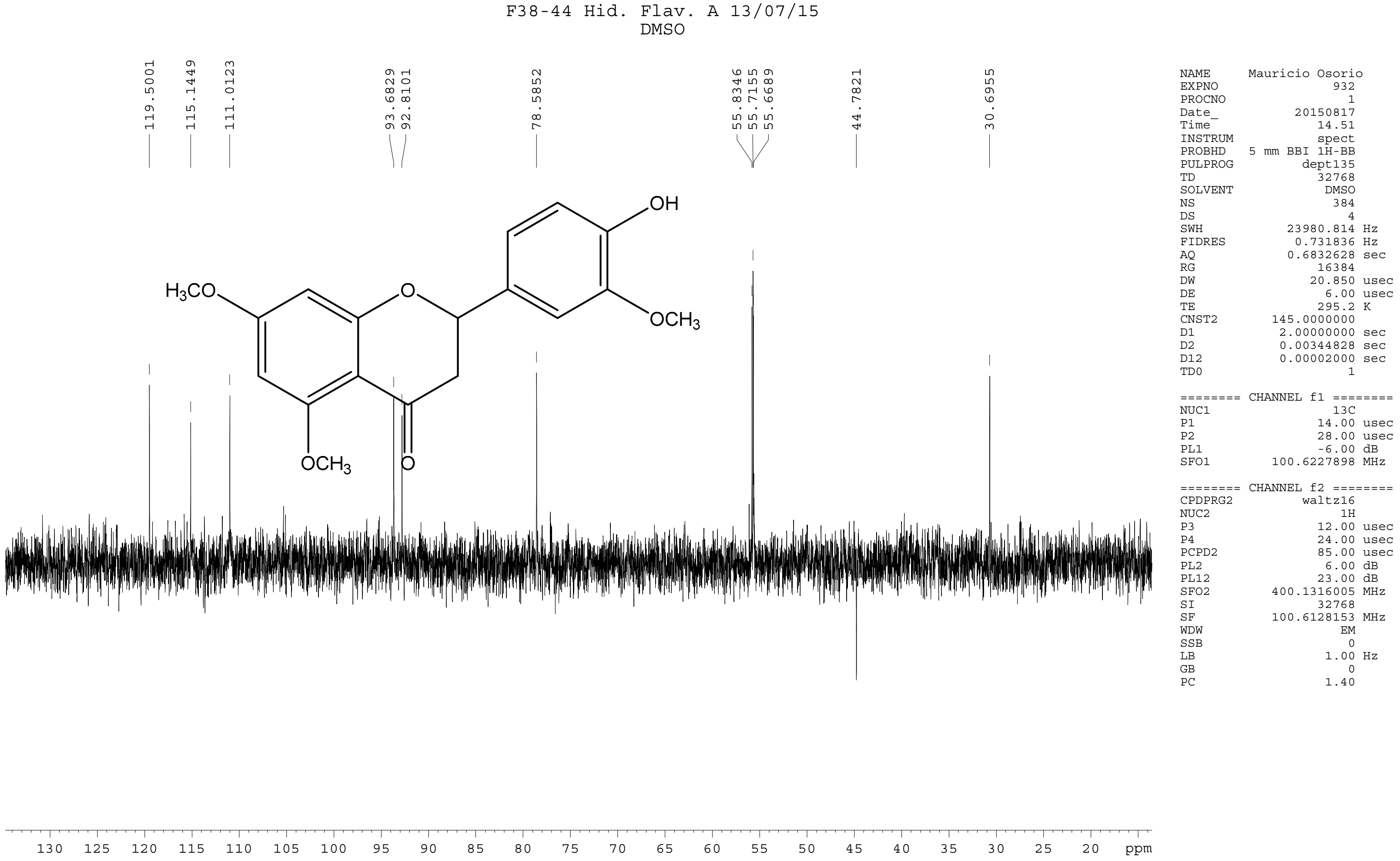

Figure S37. DEPT-135 of **FV1**.

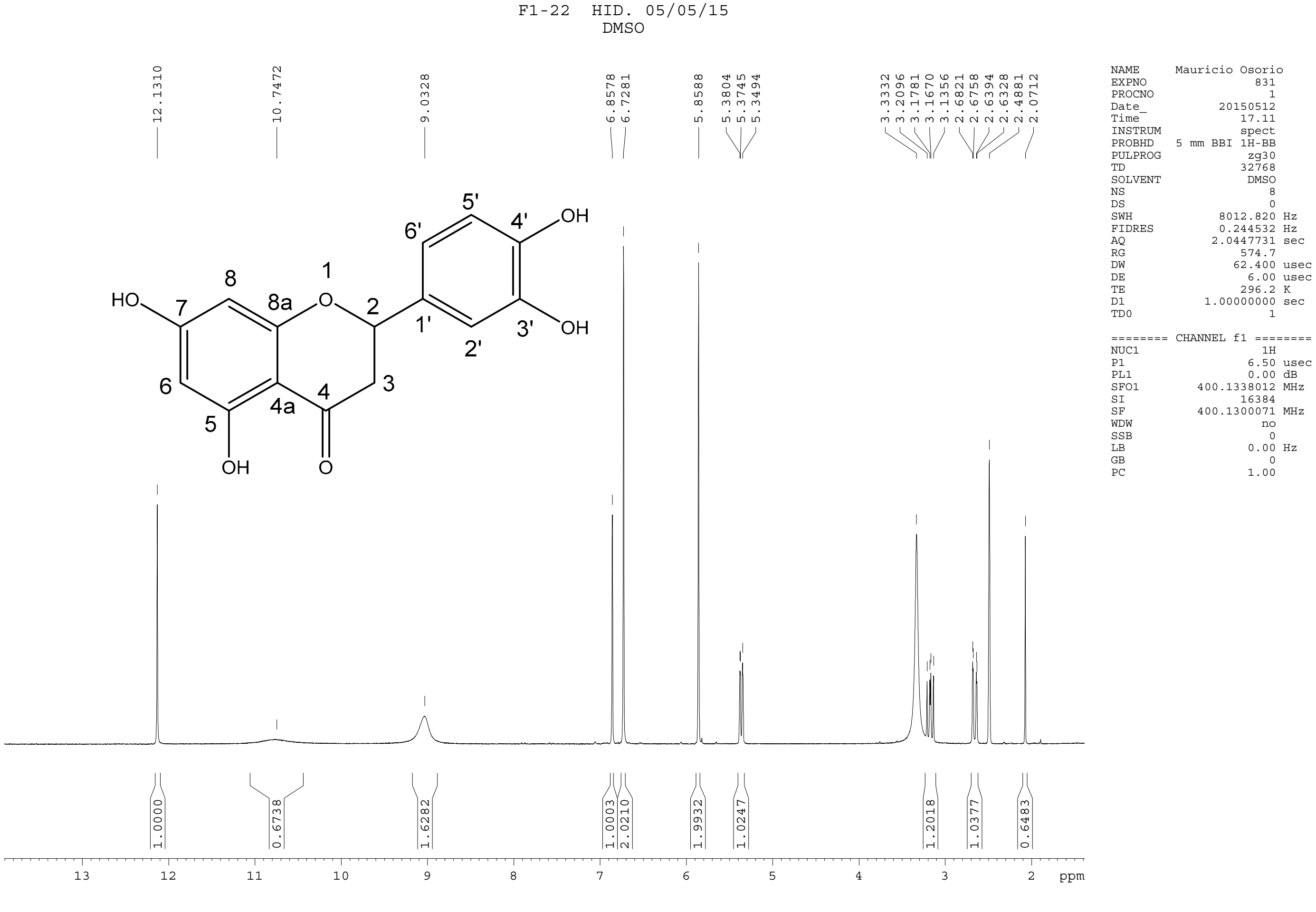

Figure S38. ^{1}H-NMR of **FV2** (DMSO-d_6).

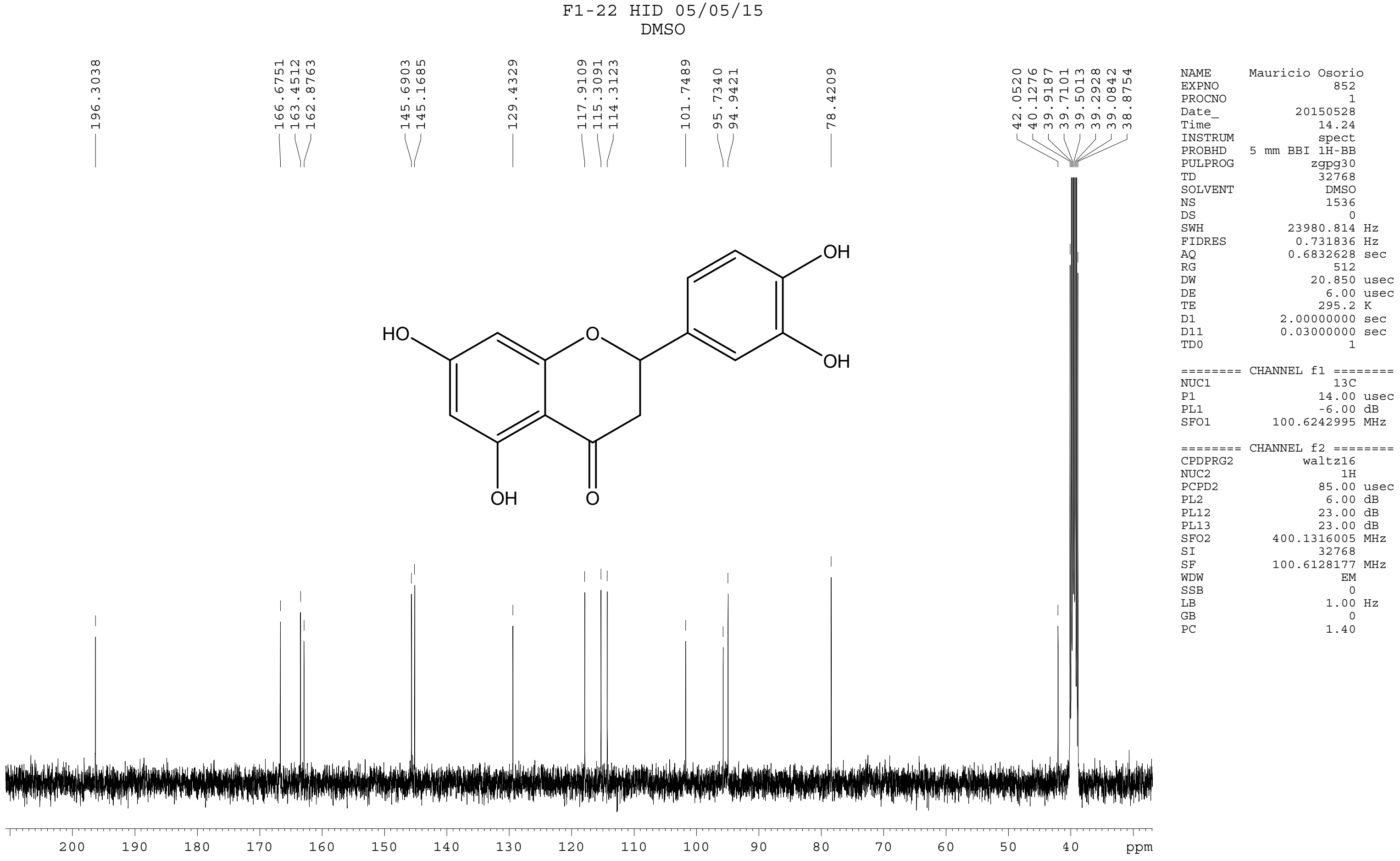

Figure S39. ^{13}C-NMR of **FV2** (DMSO-d_6).

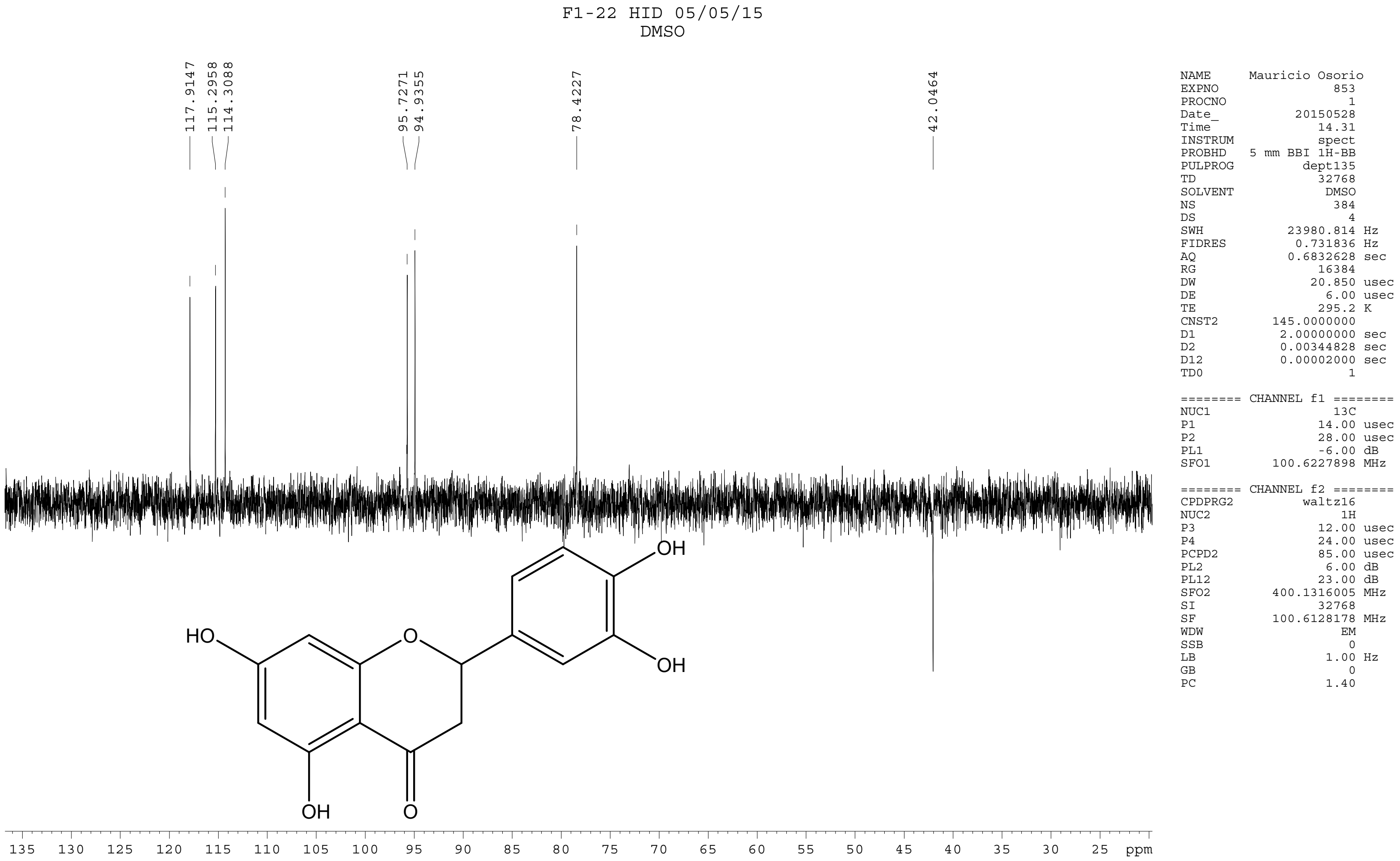

Figure S40. DEPT-135 of **FV2**.

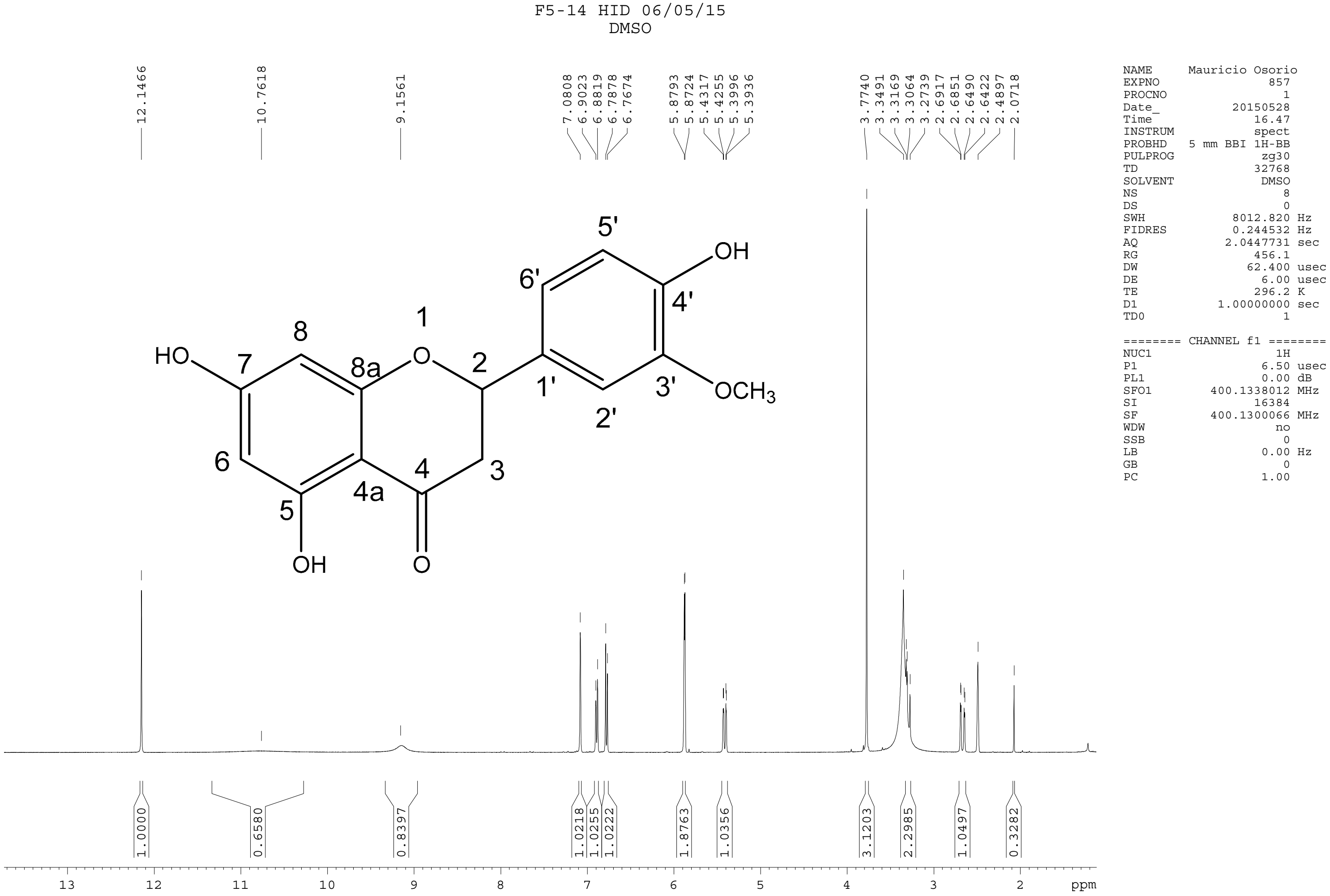

Figure S41. ^{1}H-NMR of **FV3** (DMSO-d_6).

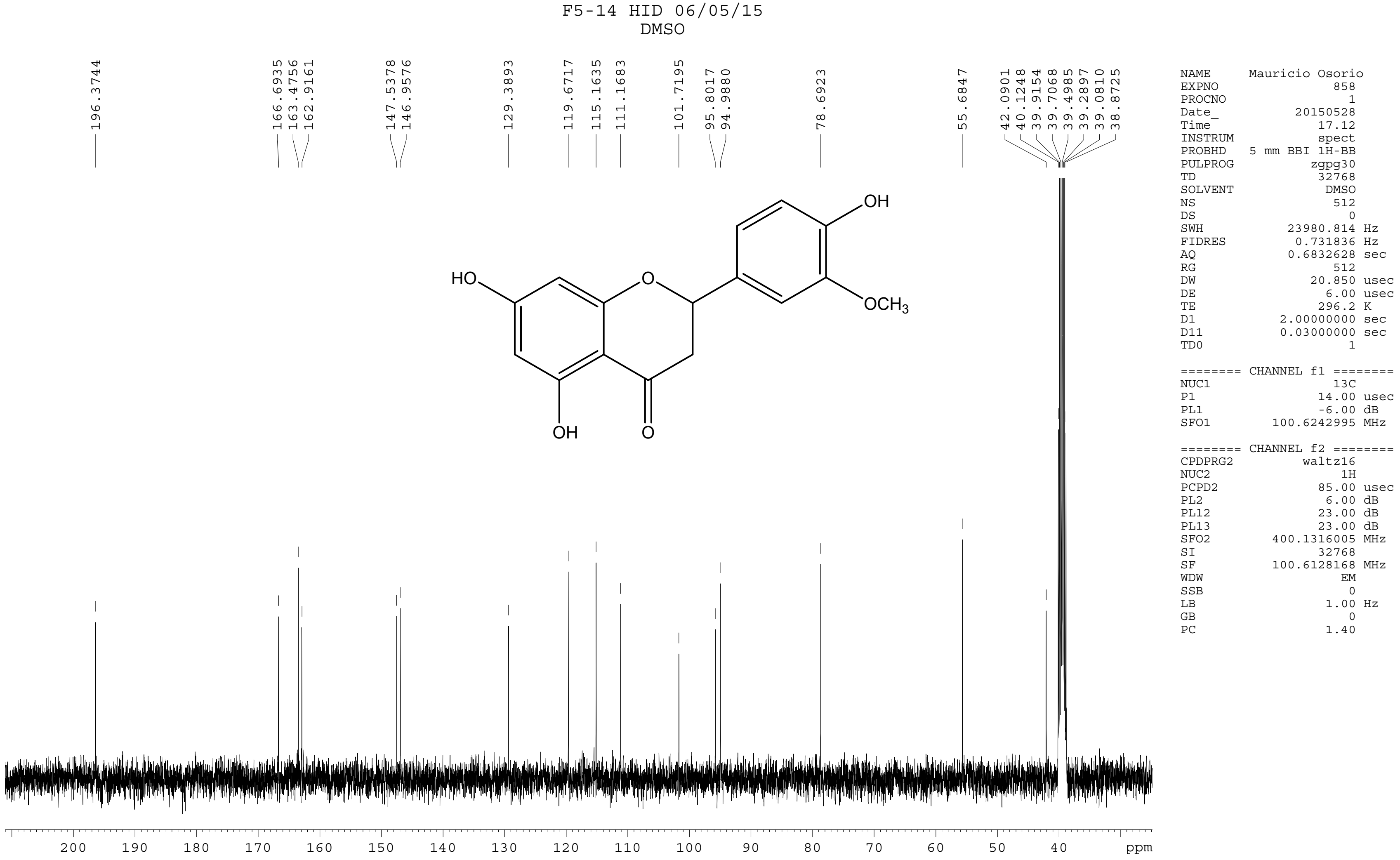

Figure S42. ^{13}C-NMR of **FV3** (DMSO-*d*$_6$).

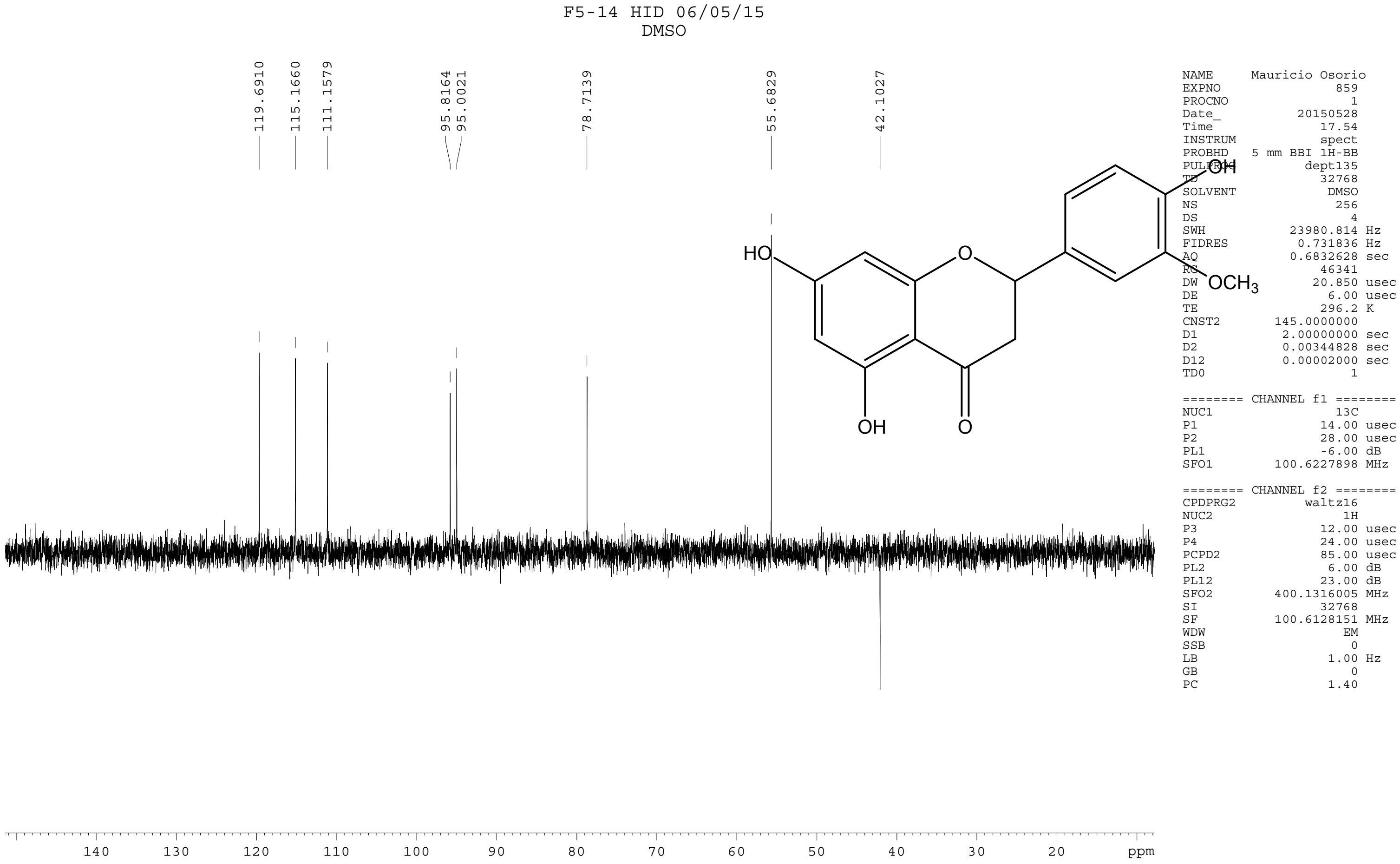

Figure S43. DEPT-135 of **FV3**.

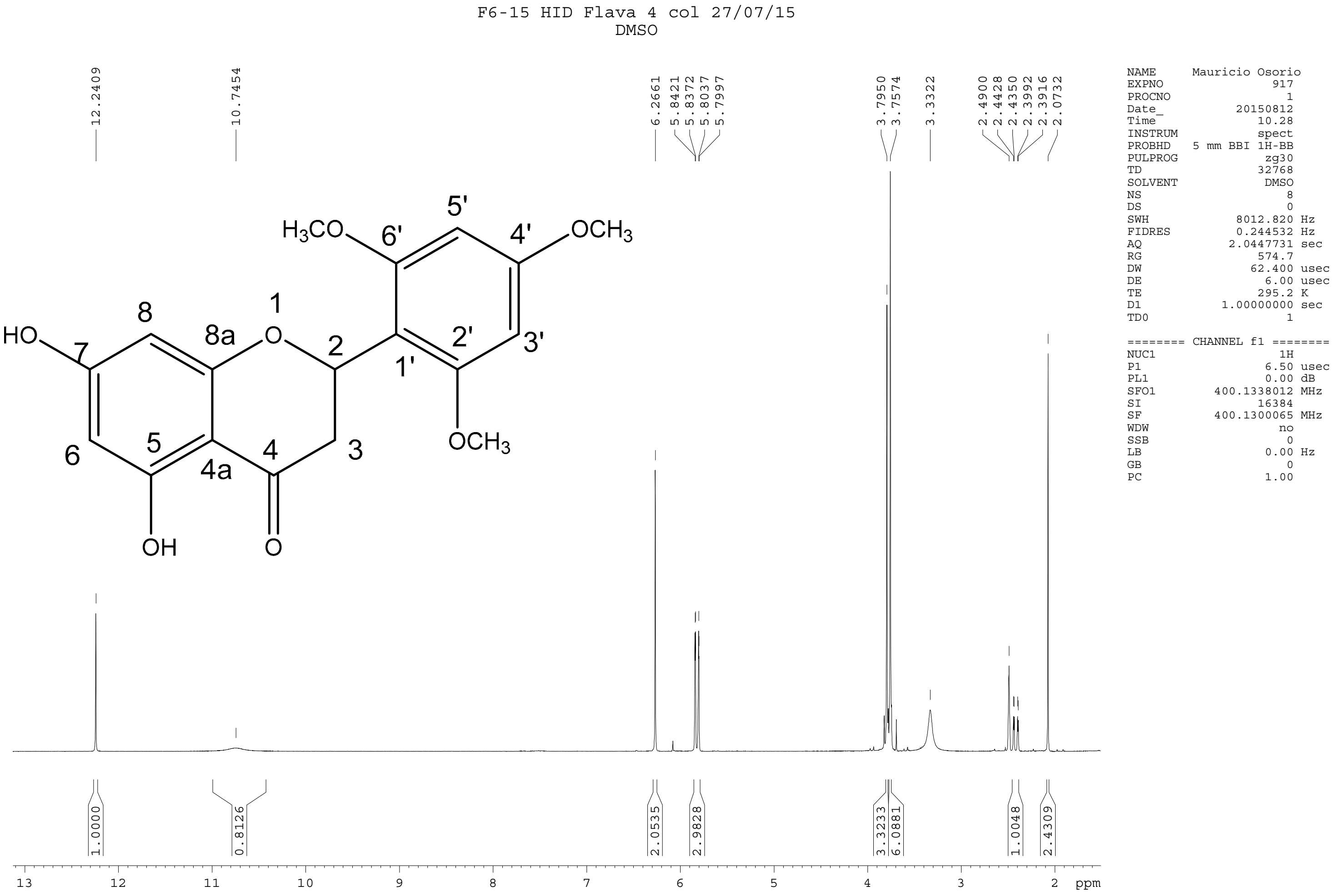

Figure S44. ^{1}H-NMR of **FV4** (DMSO-d_6).

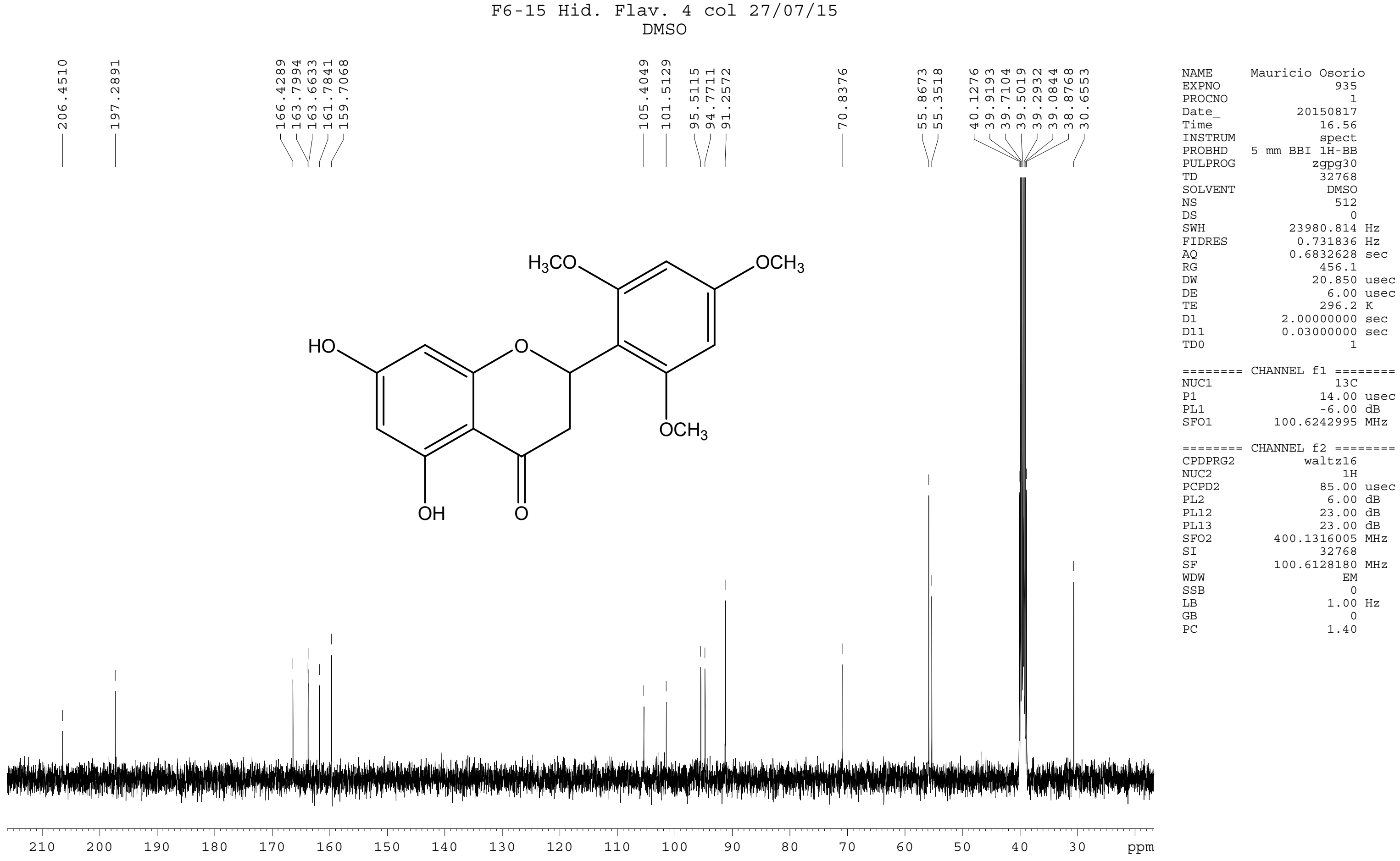

Figure S45. ^{13}C-NMR of **FV4** (DMSO-d_6).

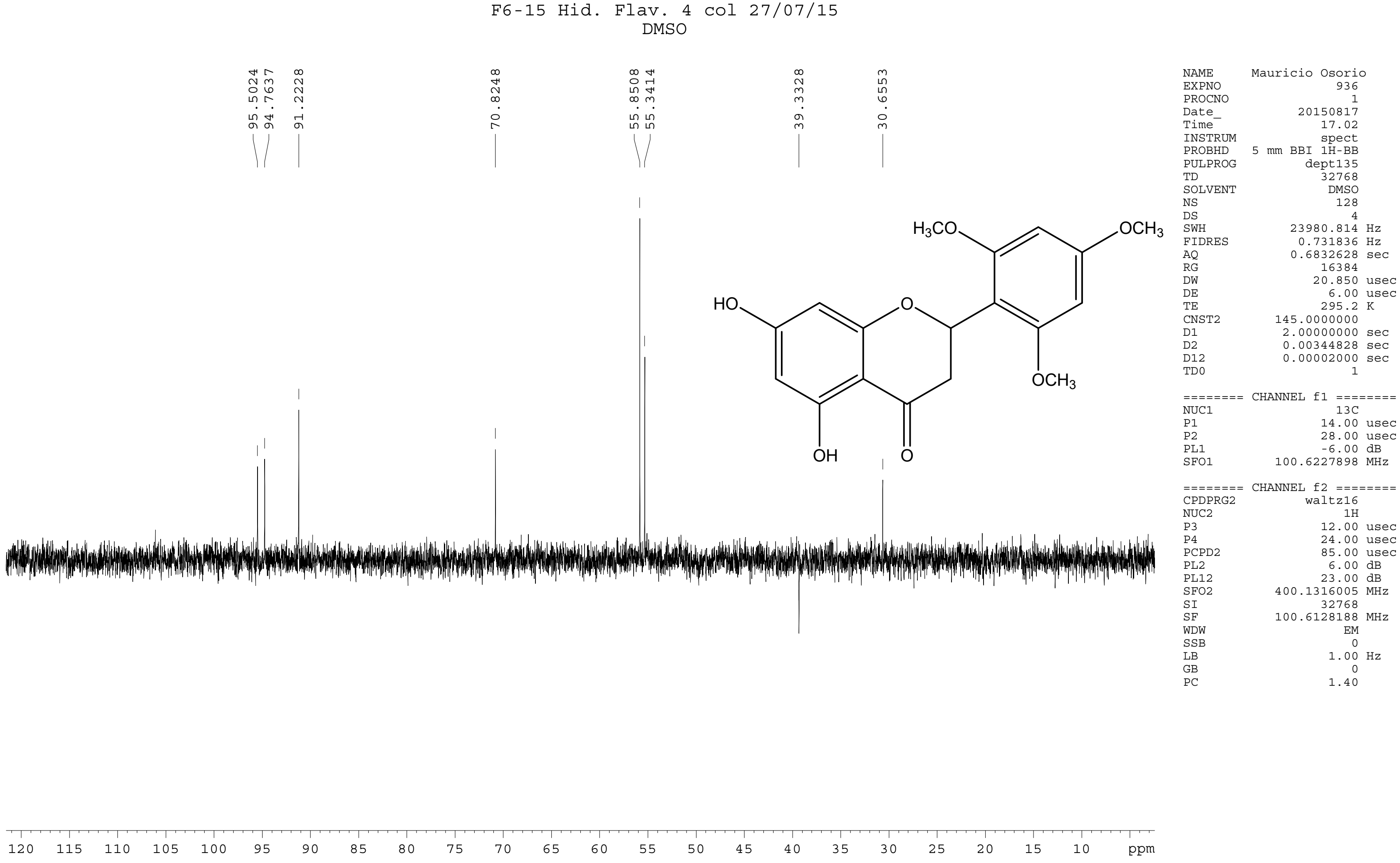

Figure S46. DEPT-135 of **FV4**.

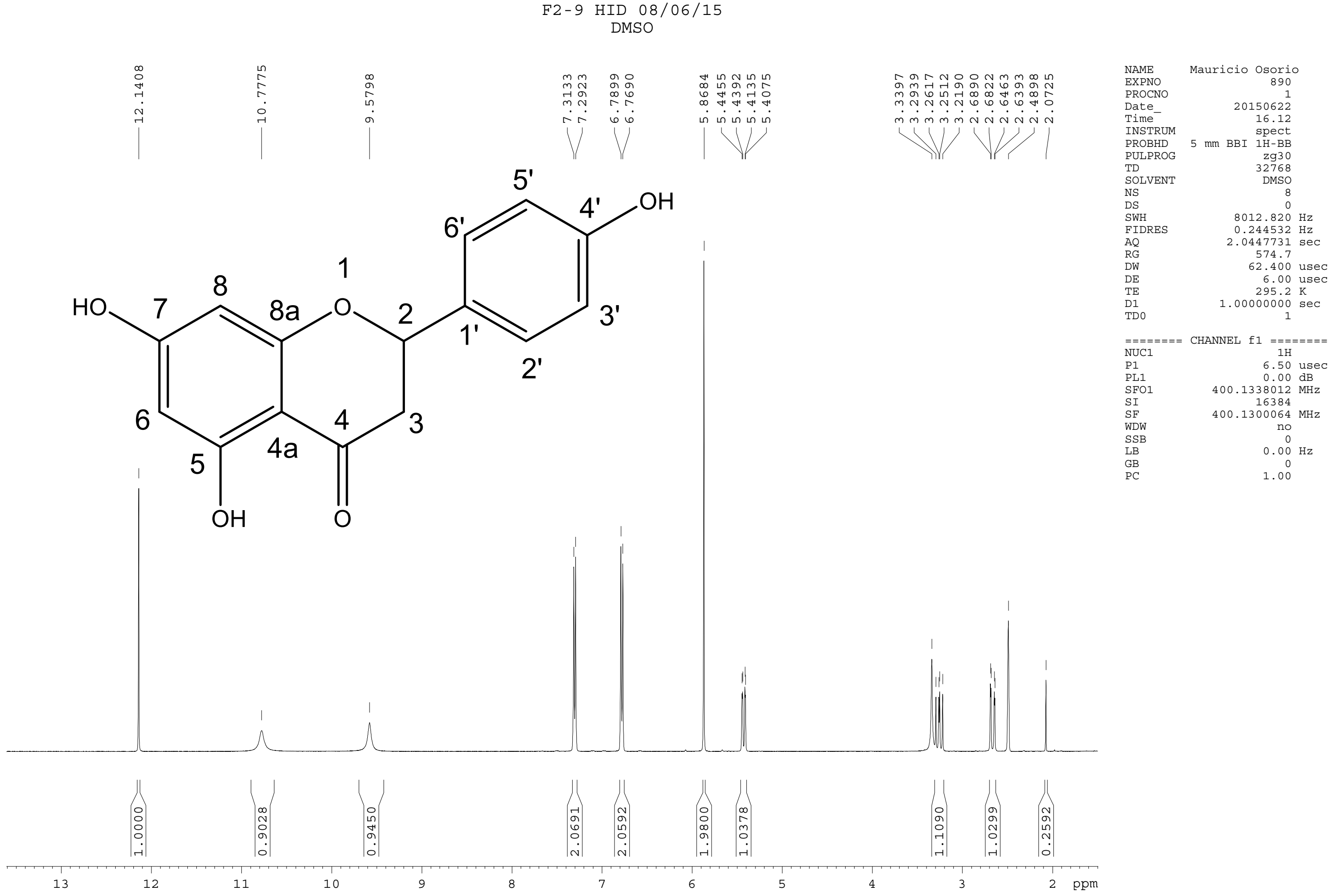

Figure S47. ^{1}H-NMR of **FV5** (DMSO-d_6).

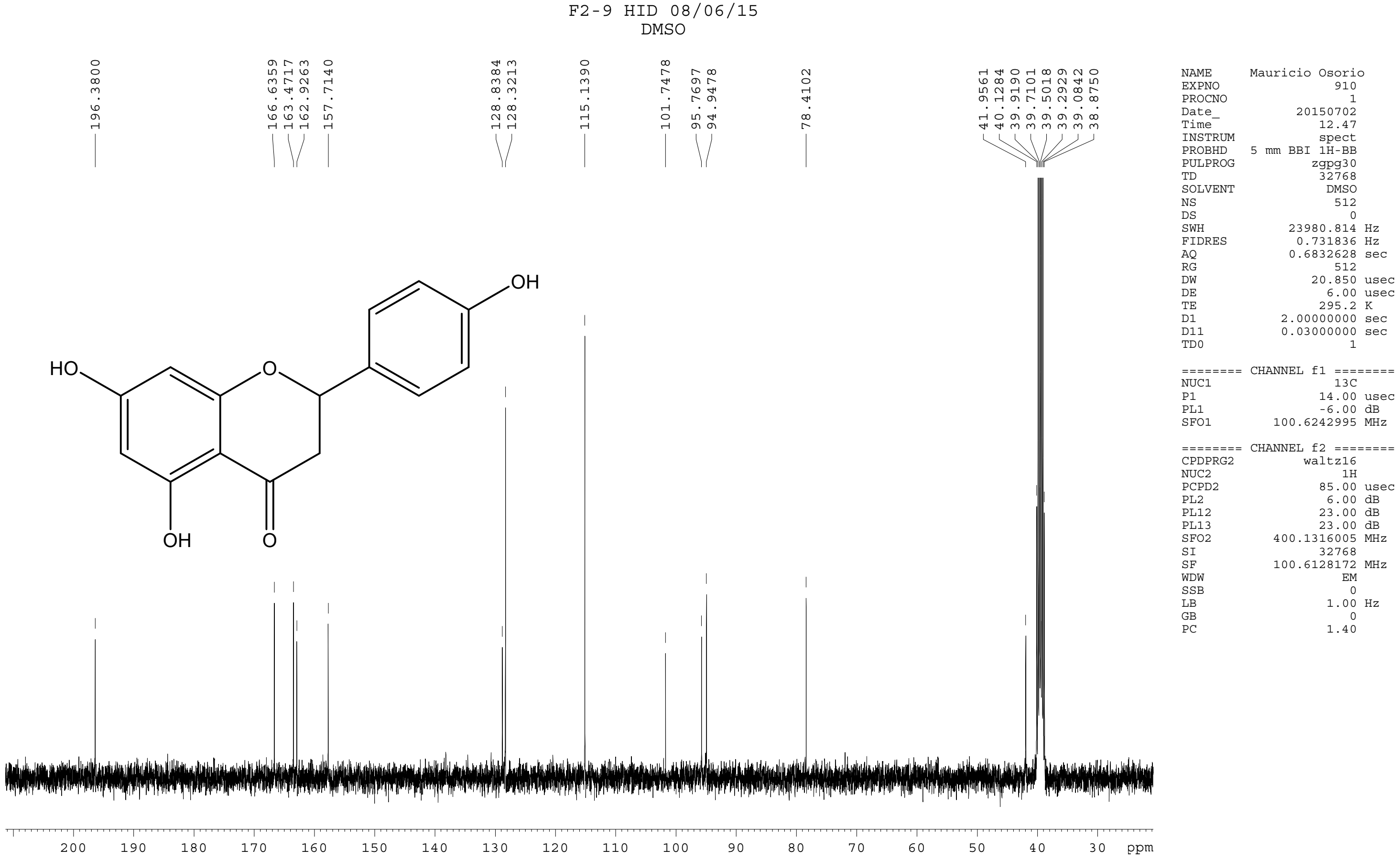

Figure S48. ^{13}C-NMR of **FV5** (DMSO-d_6).

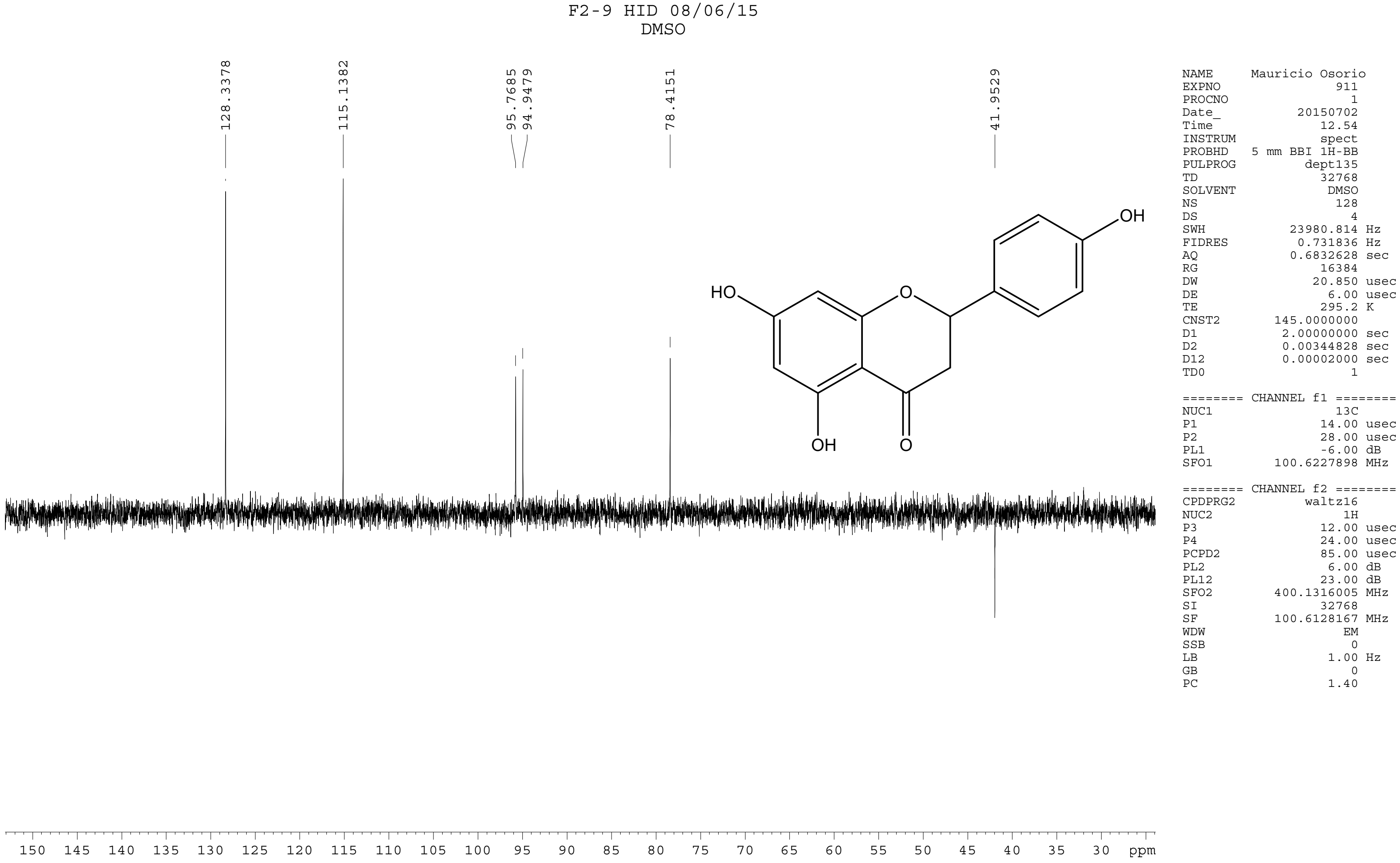

Figure S49. DEPT-135 of **FV5**.

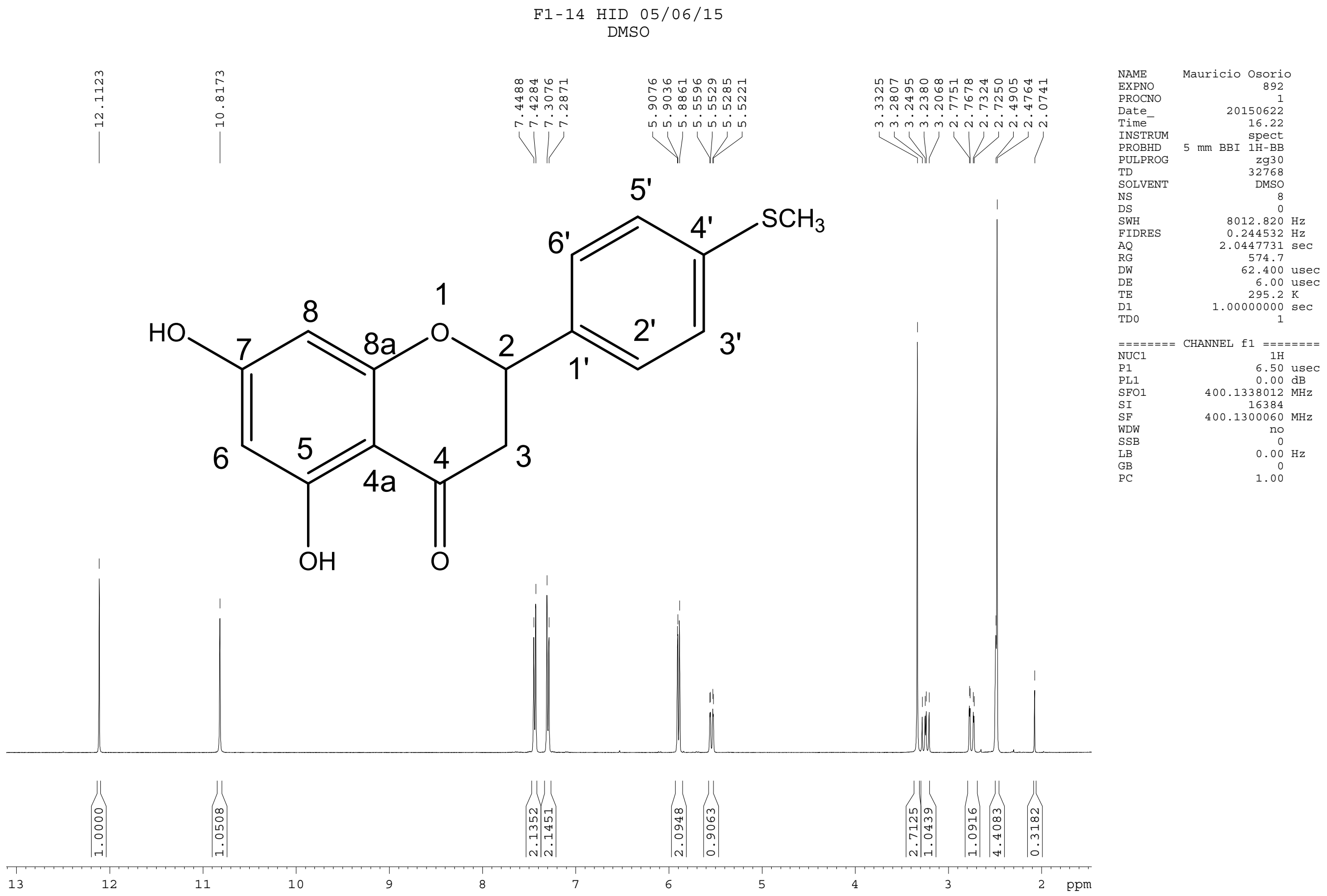

Figure S50. ^{1}H-NMR of **FV6** (DMSO-d_6).

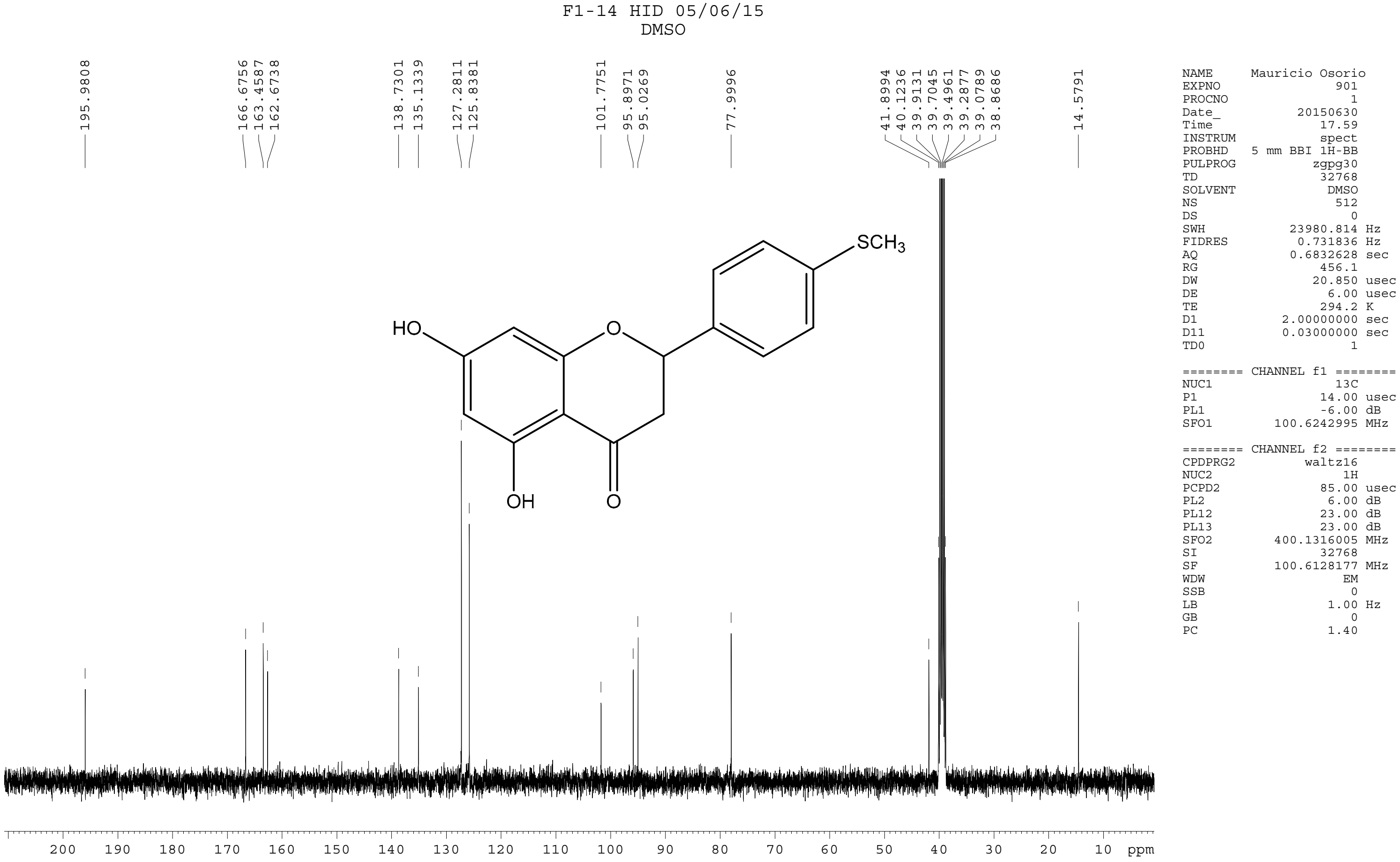

Figure S51. ^{13}C-NMR of **FV6** (DMSO-d_6).

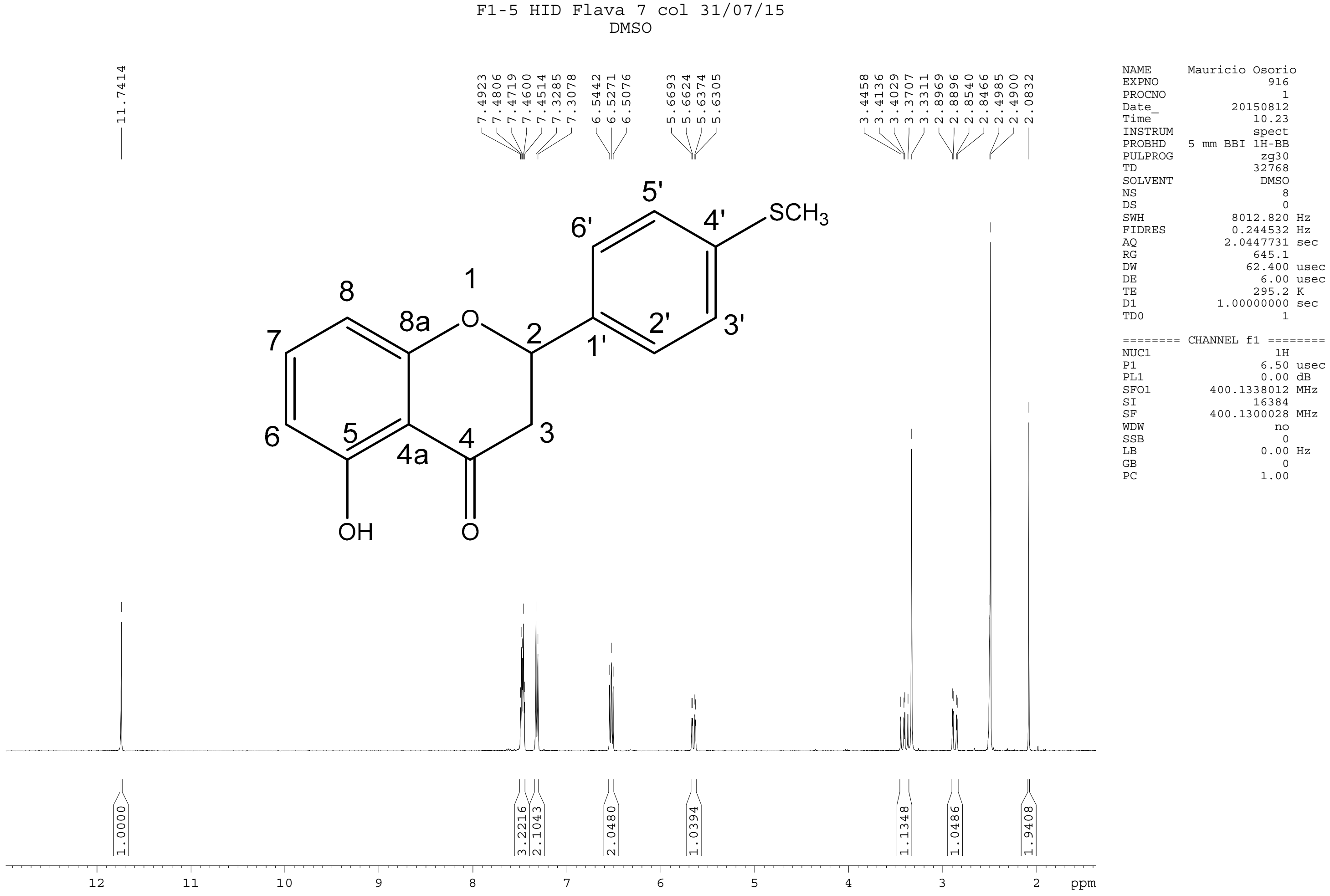

Figure S52. ^{1}H-NMR of **FV7** (DMSO-d_6).

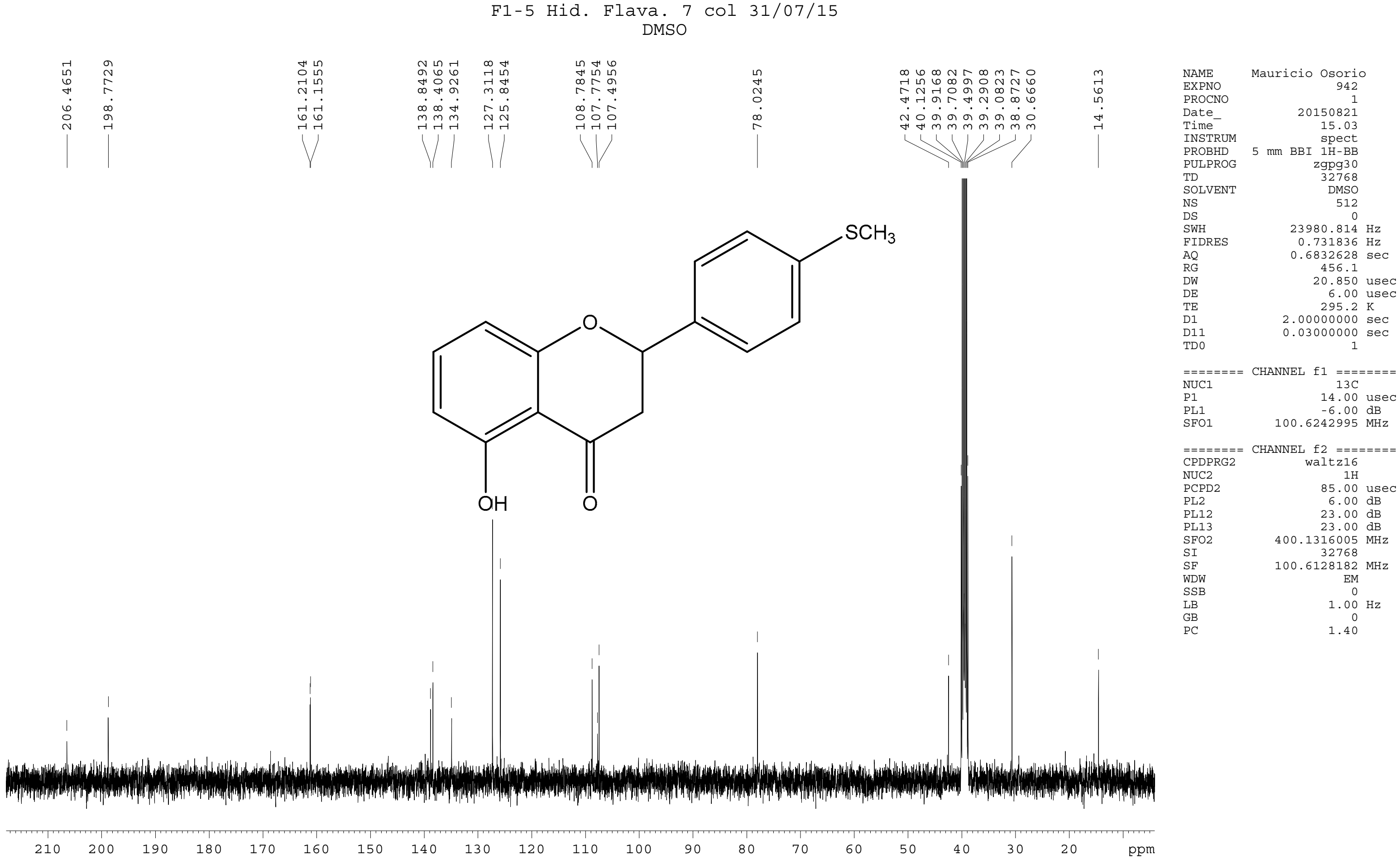

Figure S53. ^{13}C-NMR of **FV7** (DMSO-d_6).

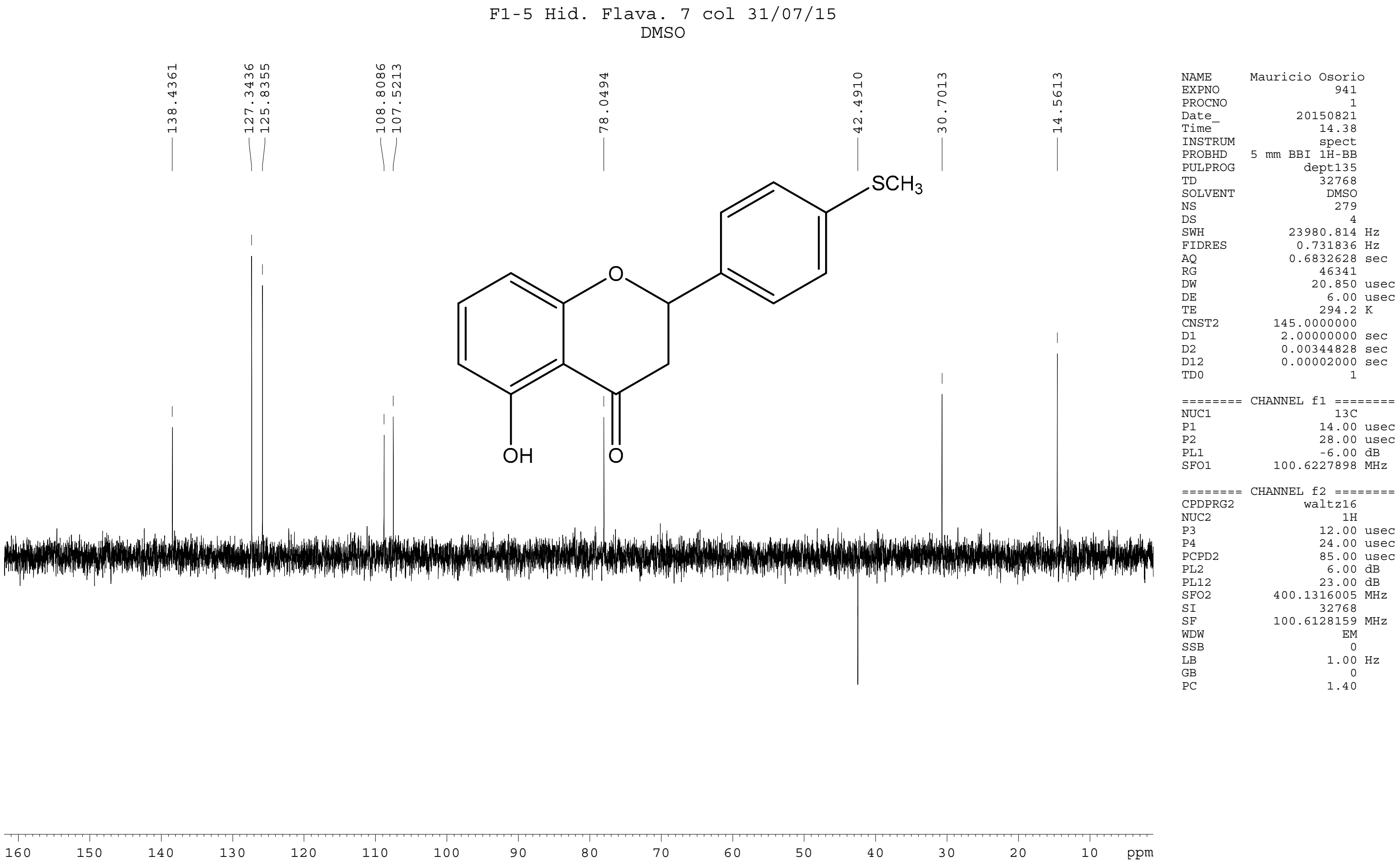

Figure S54. DEPT-135 of **FV7**.

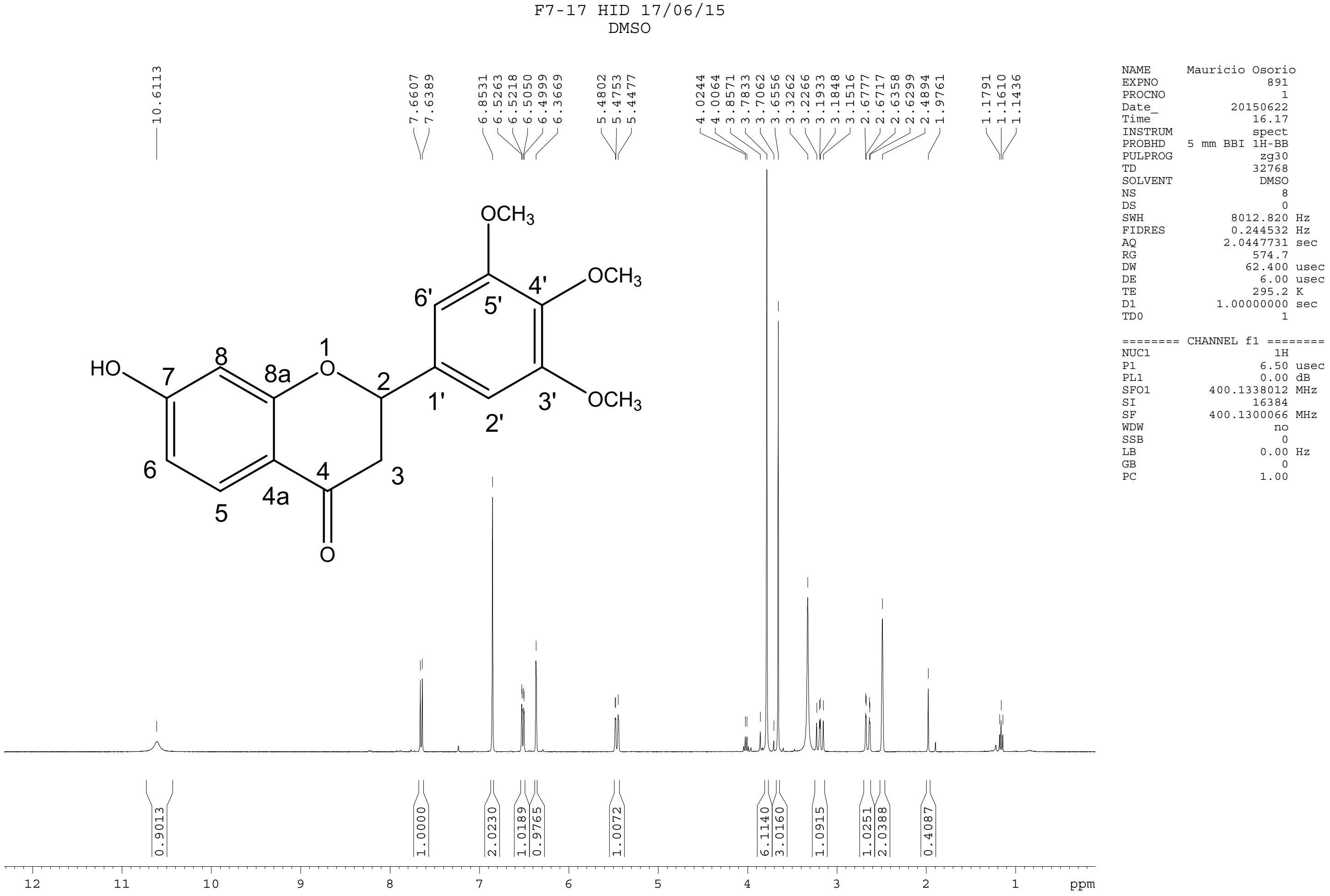

Figure S55. ^{1}H-NMR of **FV8** (DMSO-d_6).

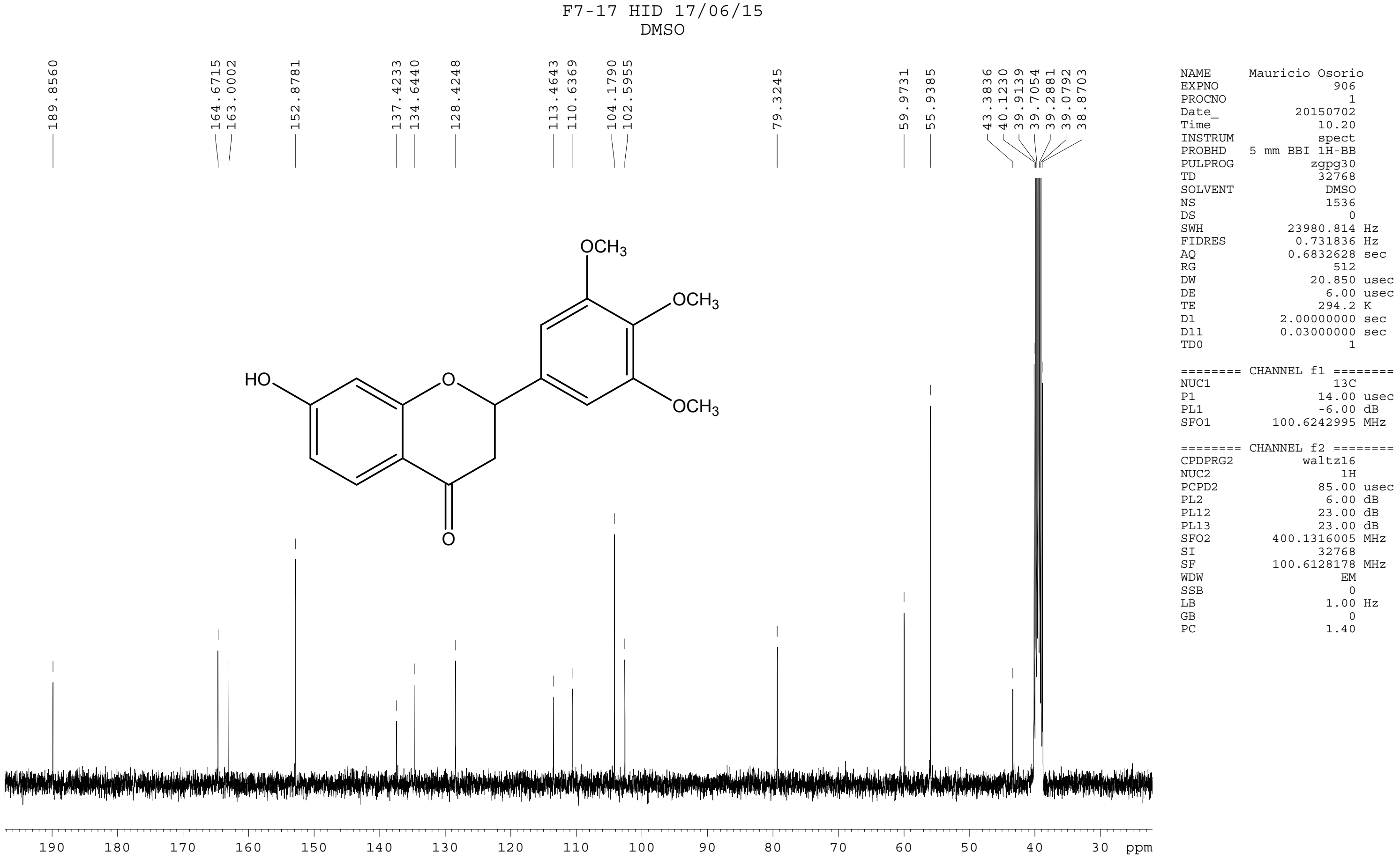

Figure S56. ^{13}C-NMR of **FV8** (DMSO-d_6).

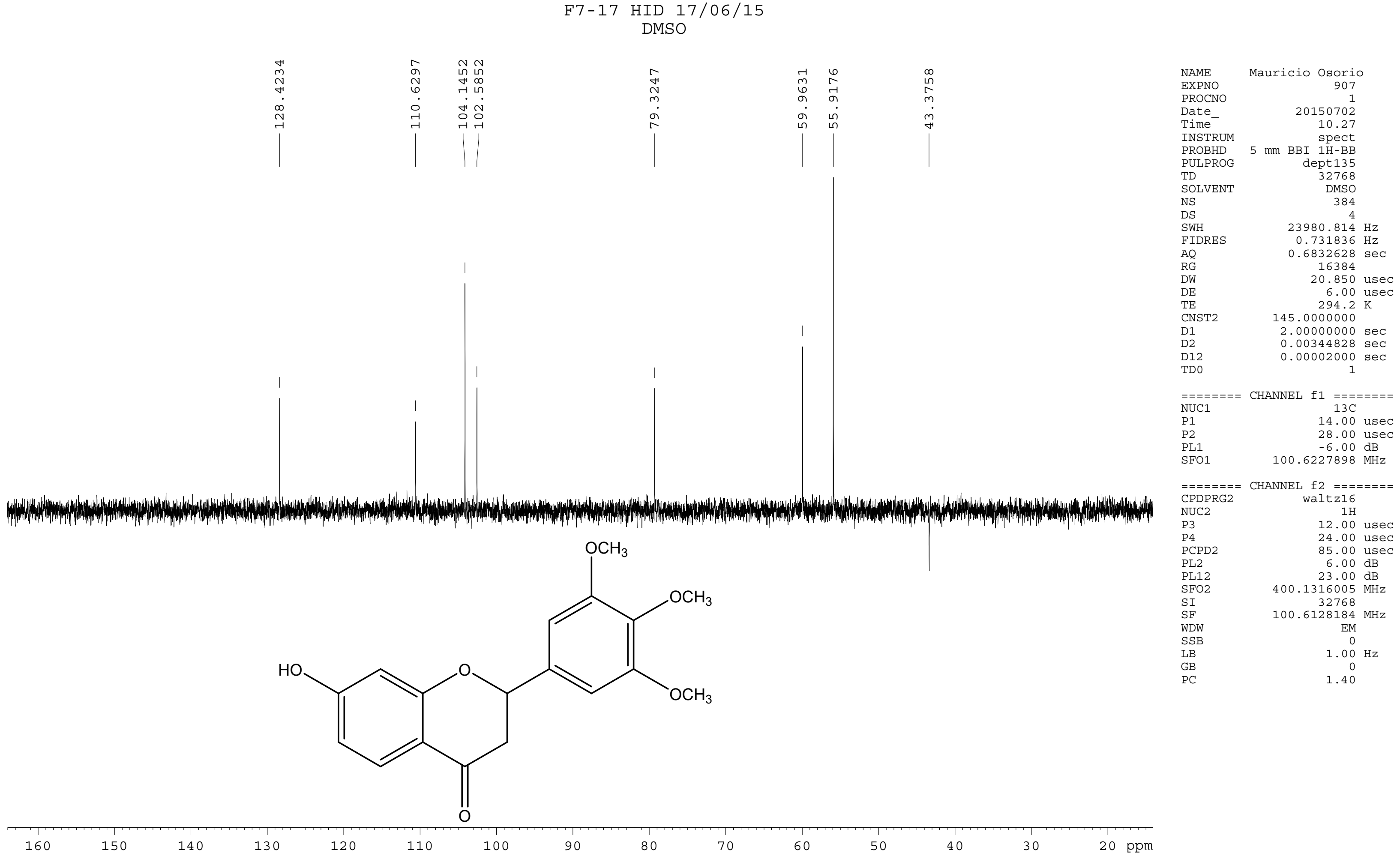

Figure S57. DEPT-135 of **FV8**.

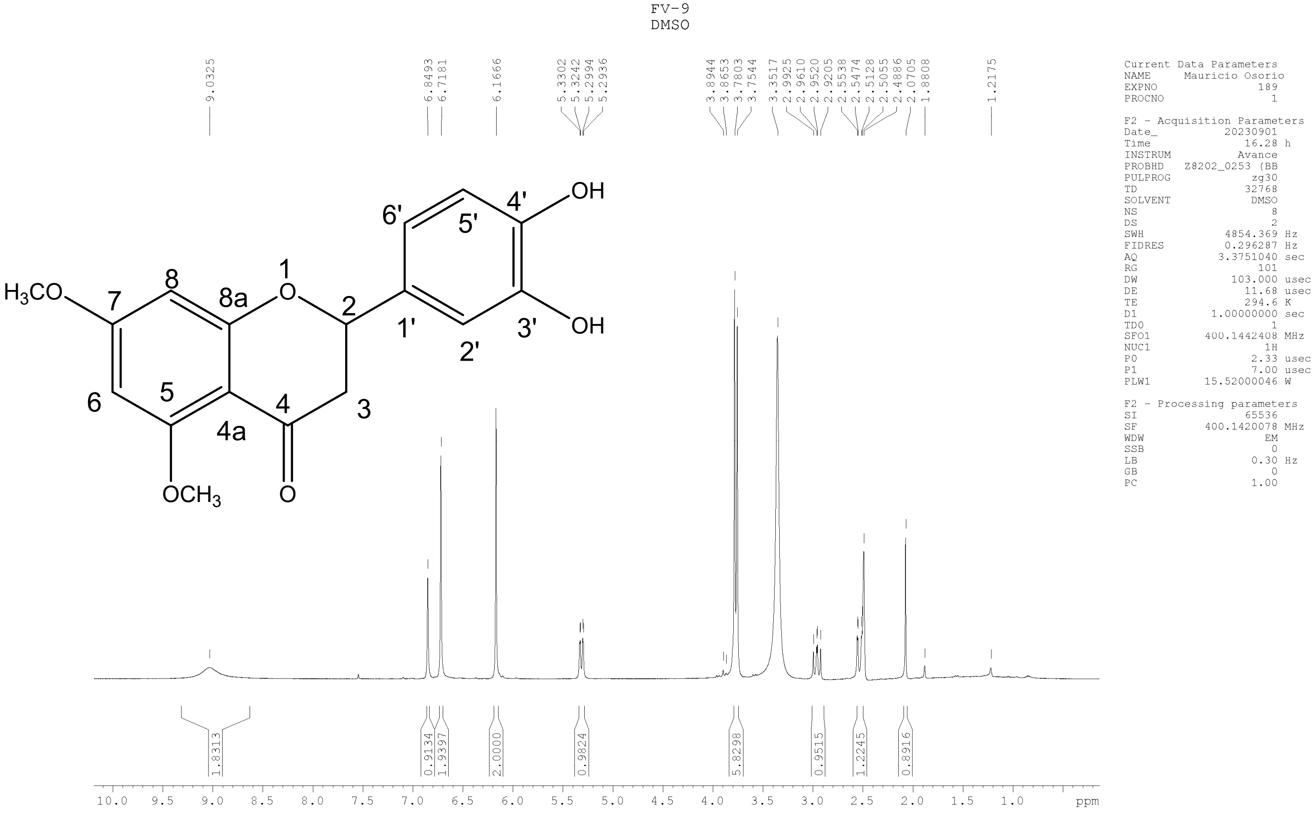

Figure S58. ^{1}H-NMR of **FV9** (DMSO-d_6).

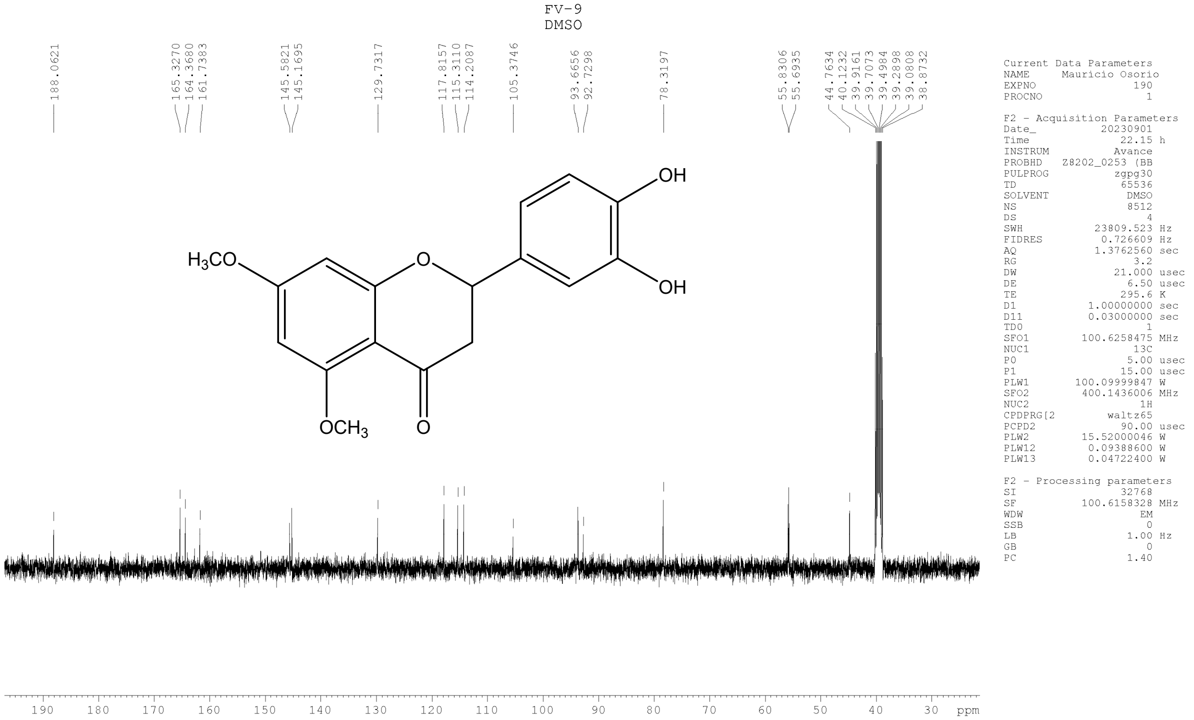

Figure S59. ^{13}C-NMR of **FV9** (DMSO-d_6).

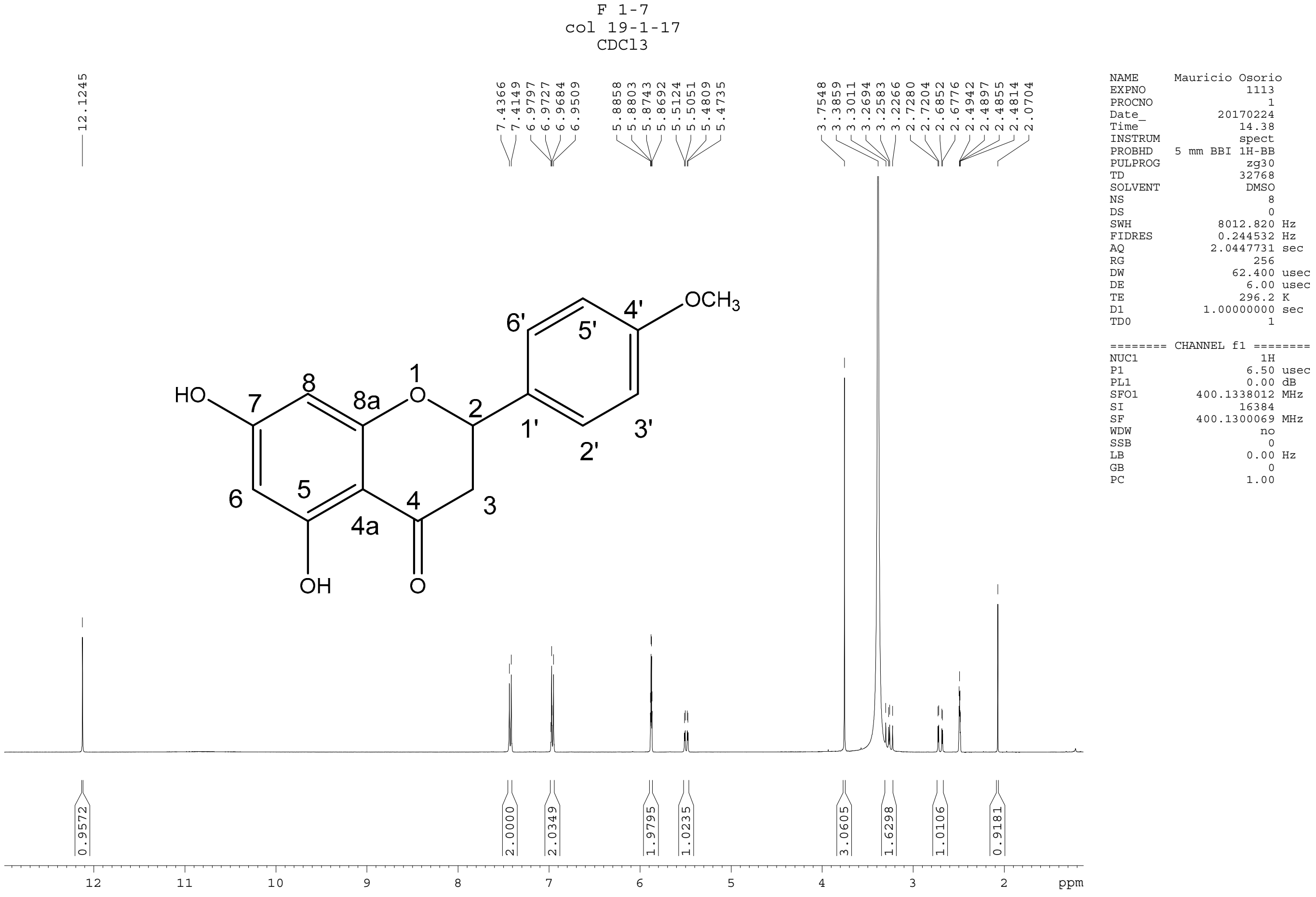

Figure S60. ^{1}H-NMR of **FV10** (DMSO-d_6).

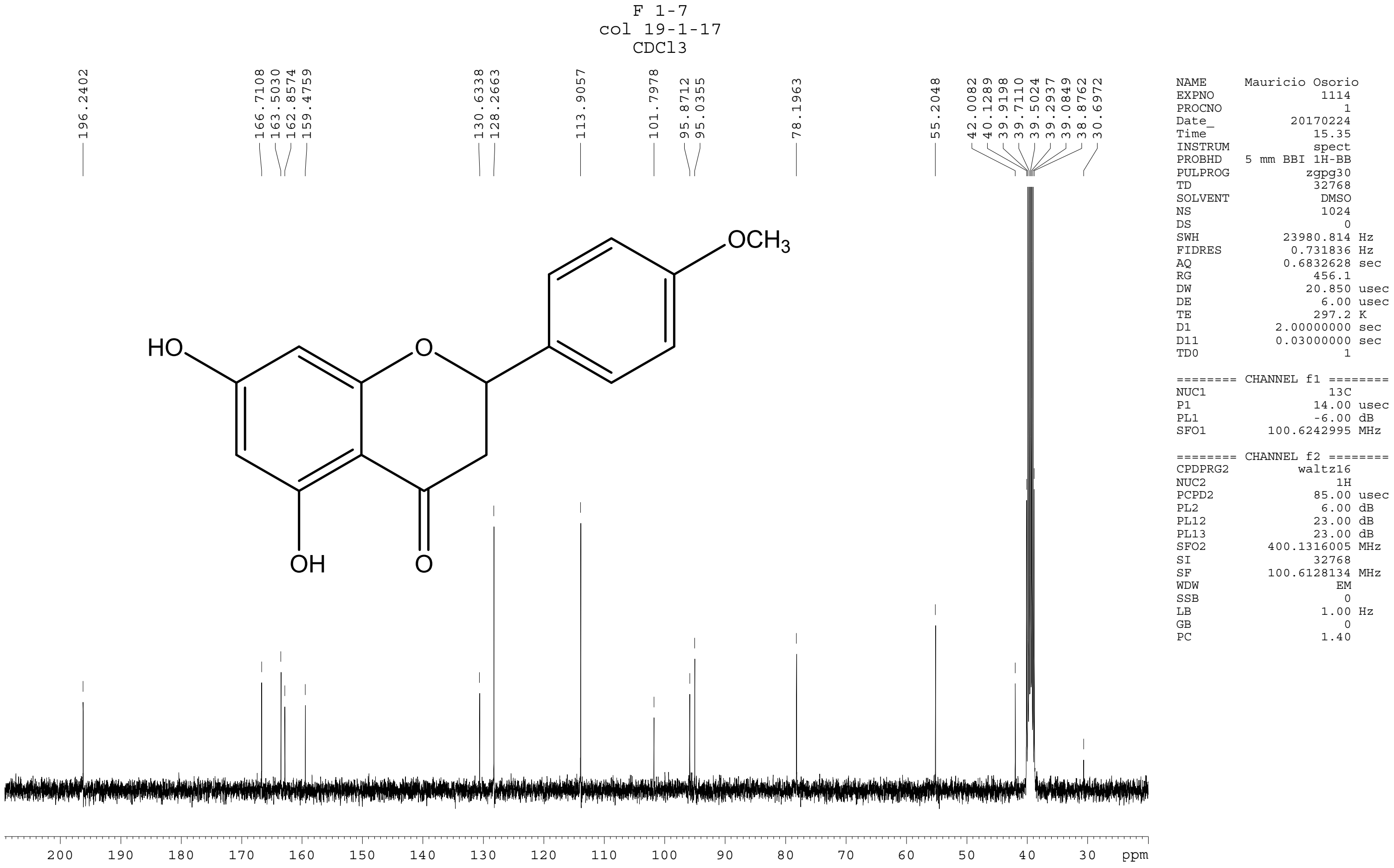

Figure S61. ^{13}C-NMR of **FV10** (DMSO-d_6).

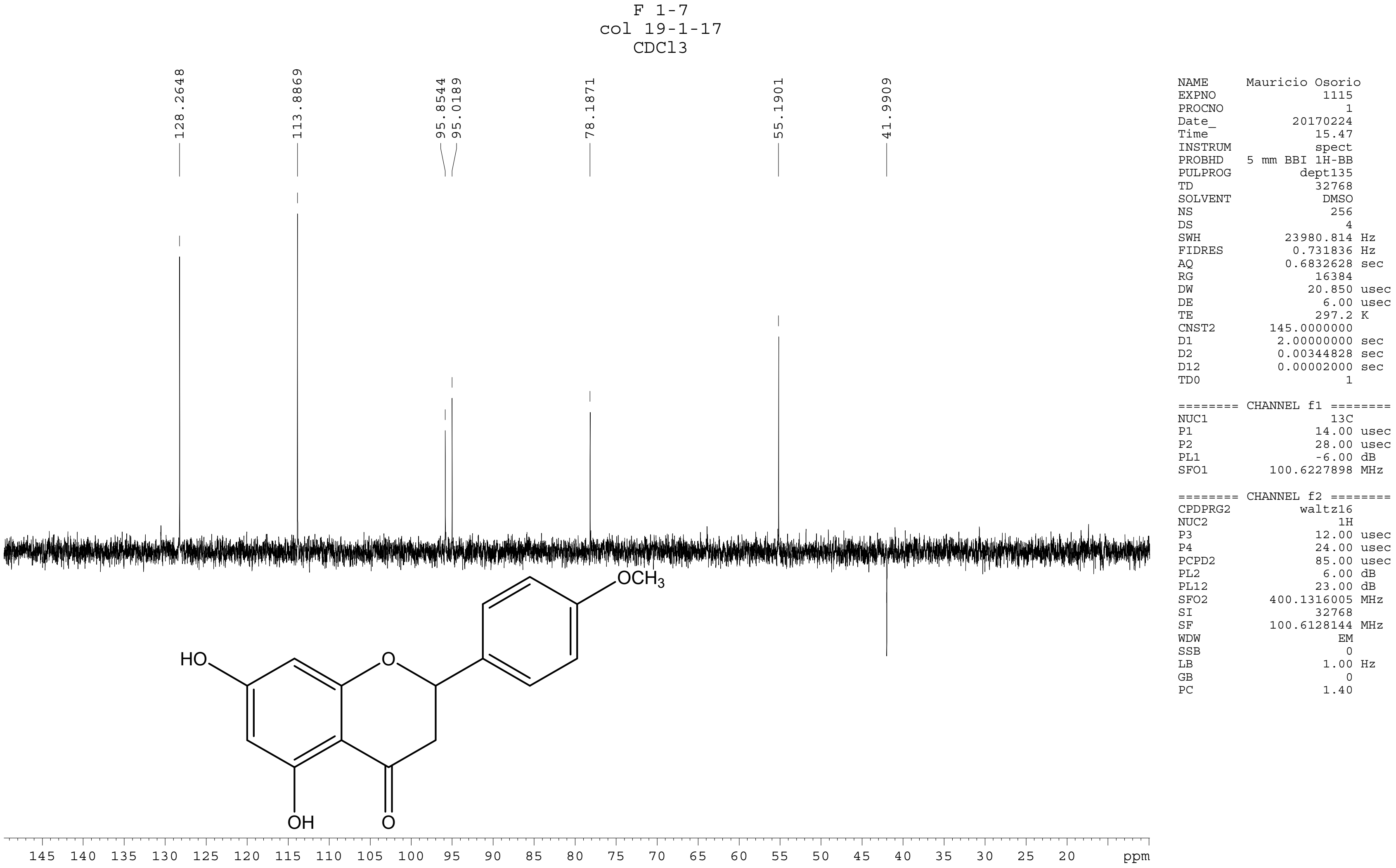

Figure S62. DEPT-135 of **FV10**.

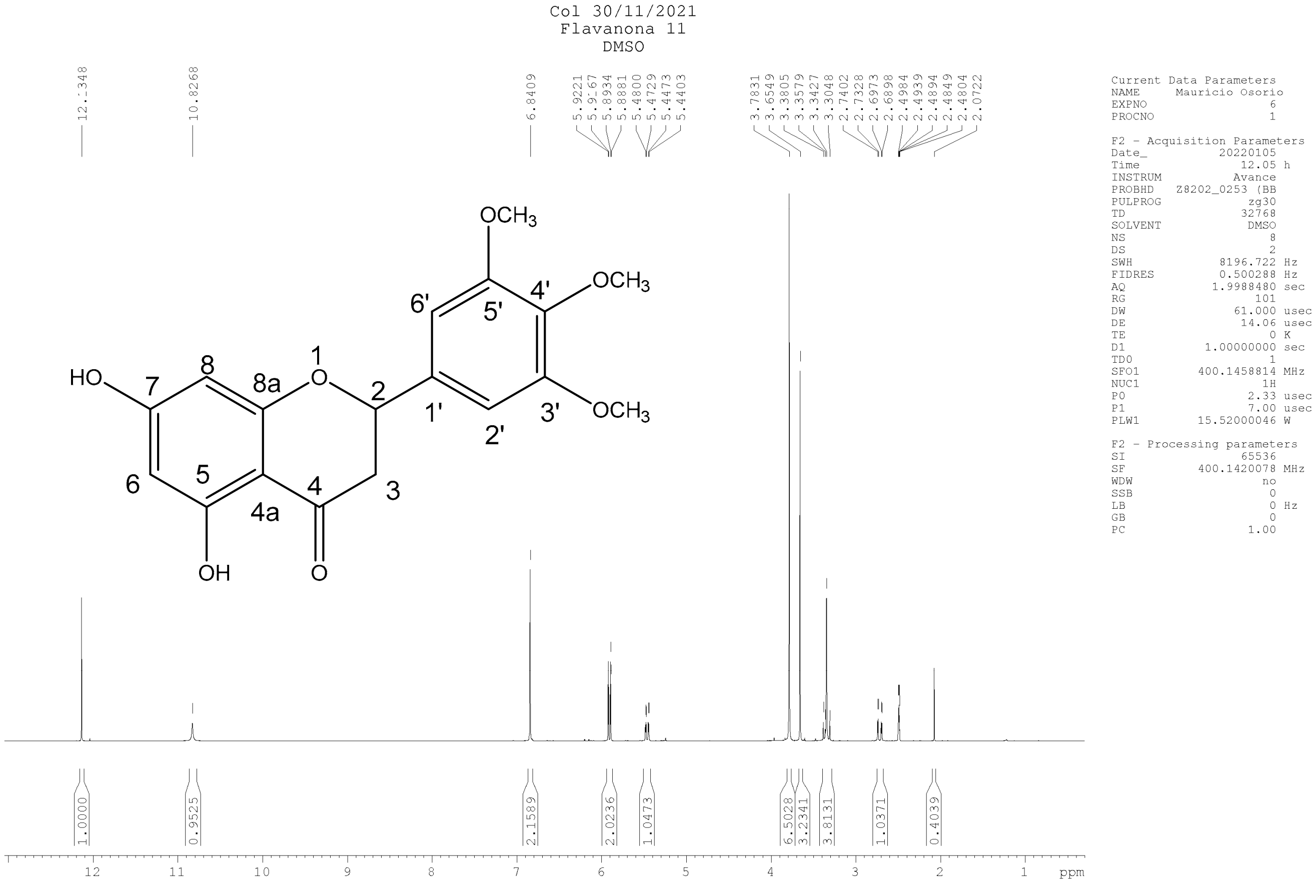

Figure S63. ^{1}H-NMR of **FV11** (DMSO-d_6).

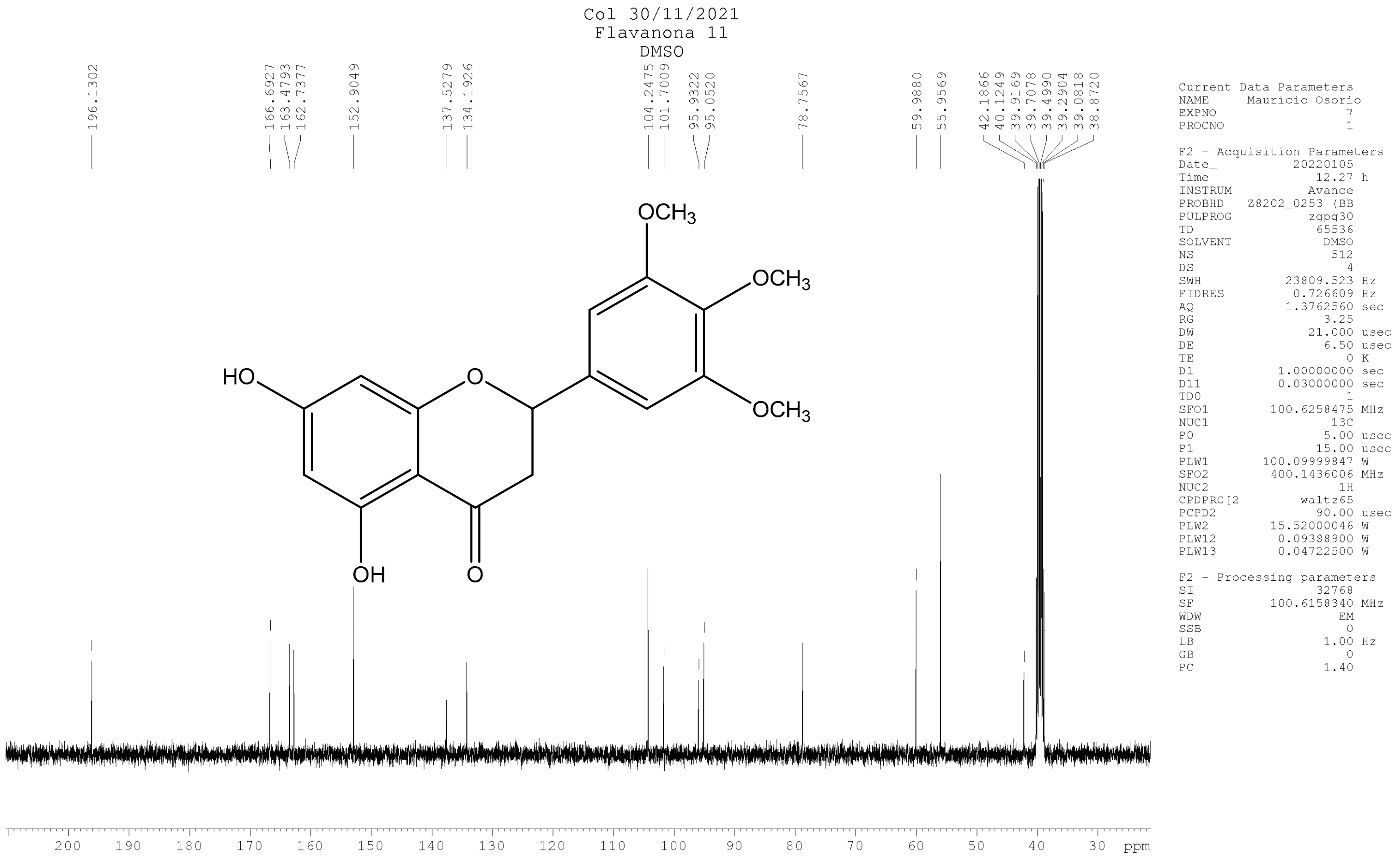

Figure S64. ^{13}C-NMR of **FV11** (DMSO-d_6).

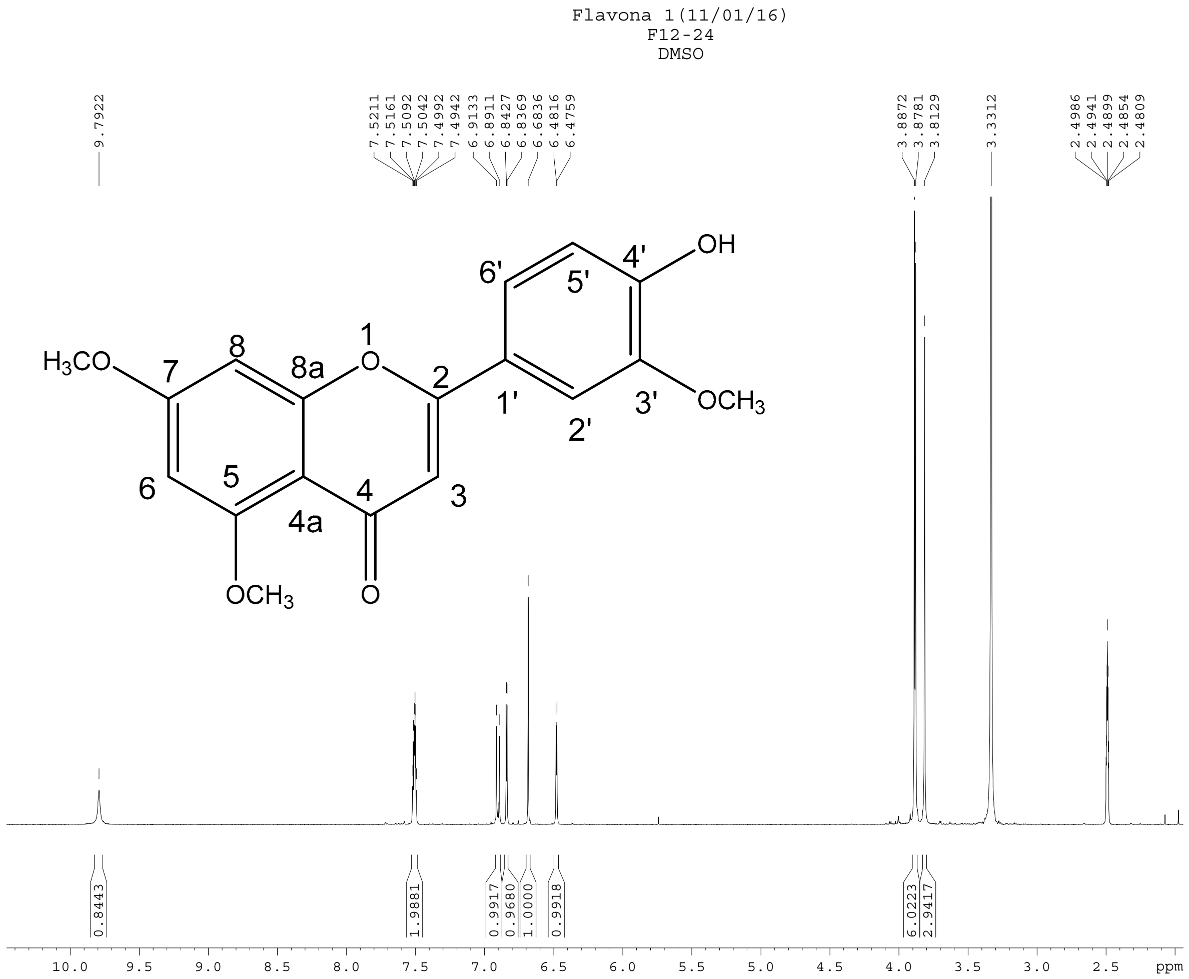

Figure S65. ^{1}H-NMR of **FO1** (DMSO-*d*$_6$).

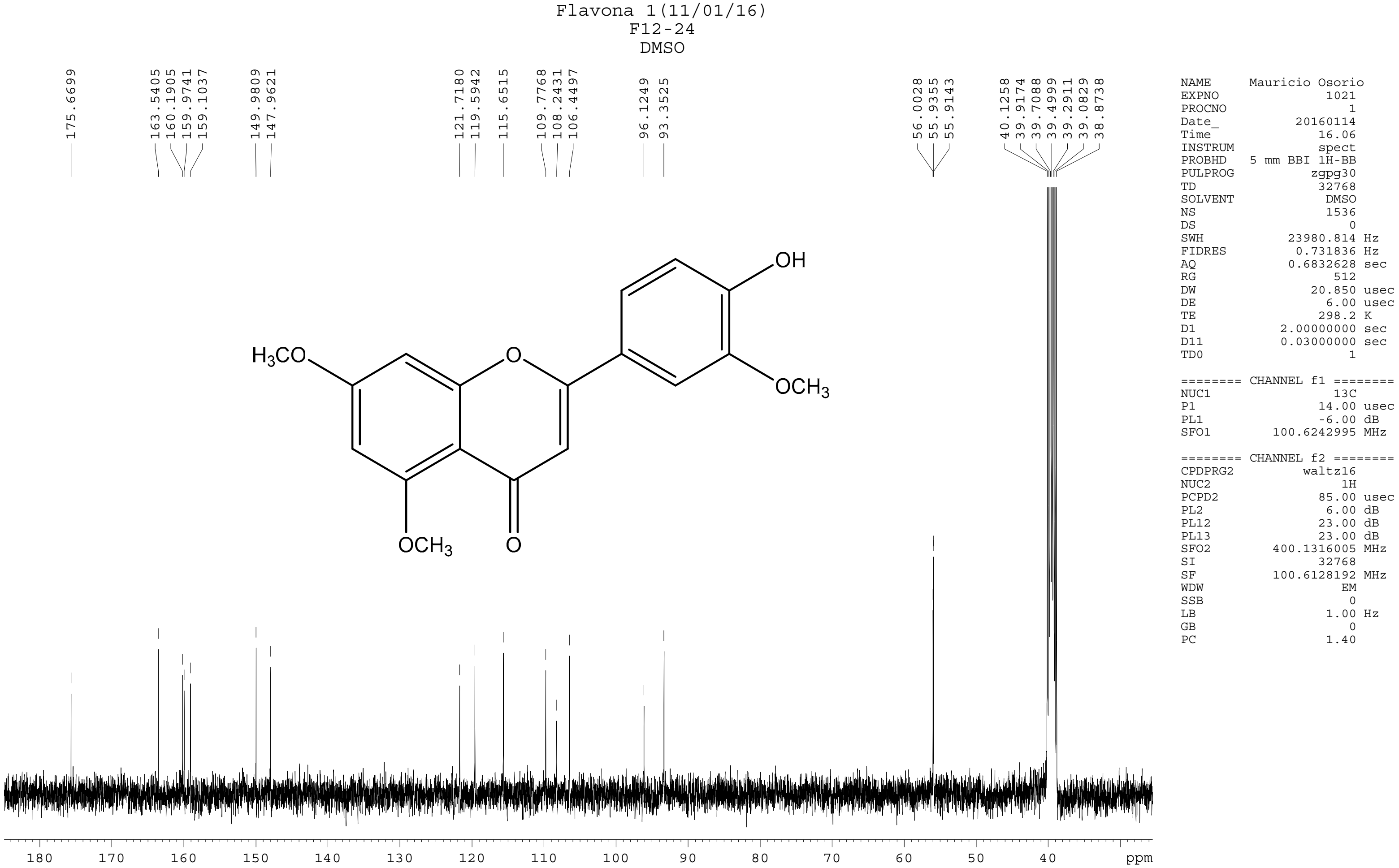

Figure S66. ^{13}C-NMR of **FO1** (DMSO-d_6).

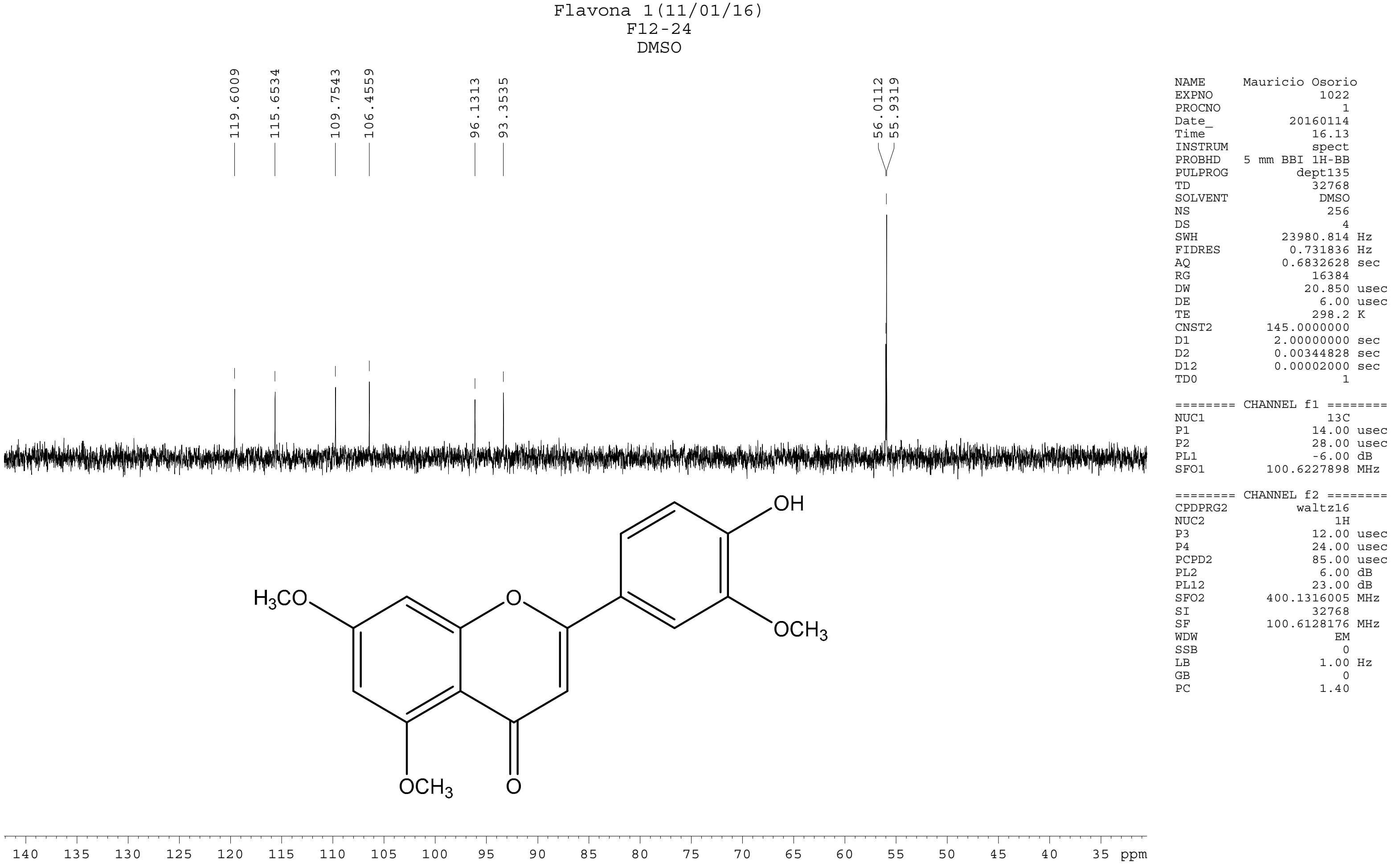

Figure S67. DEPT-135 of **FO1**.

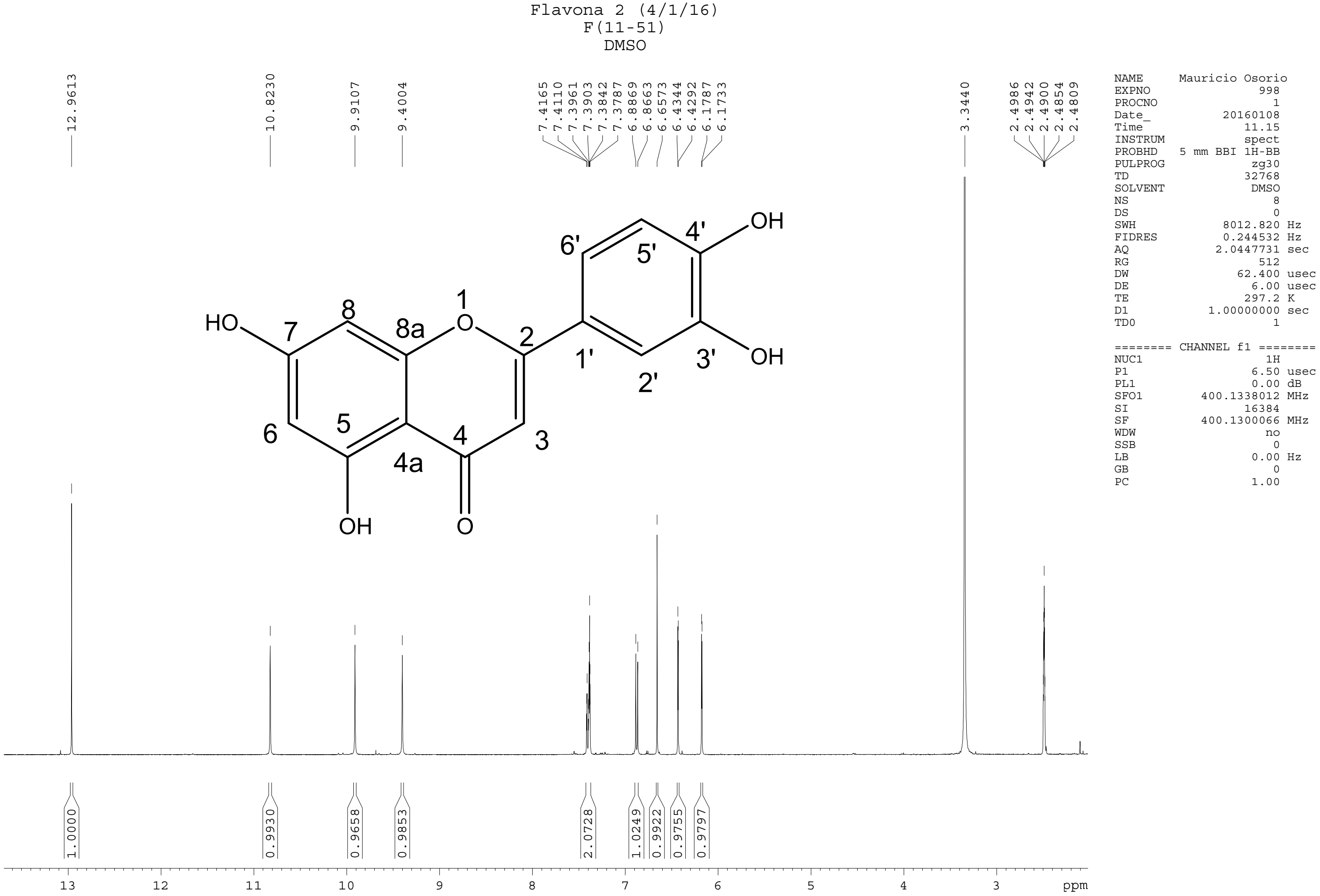

Figure S68. ^{1}H-NMR of **FO2** (DMSO-d_6).

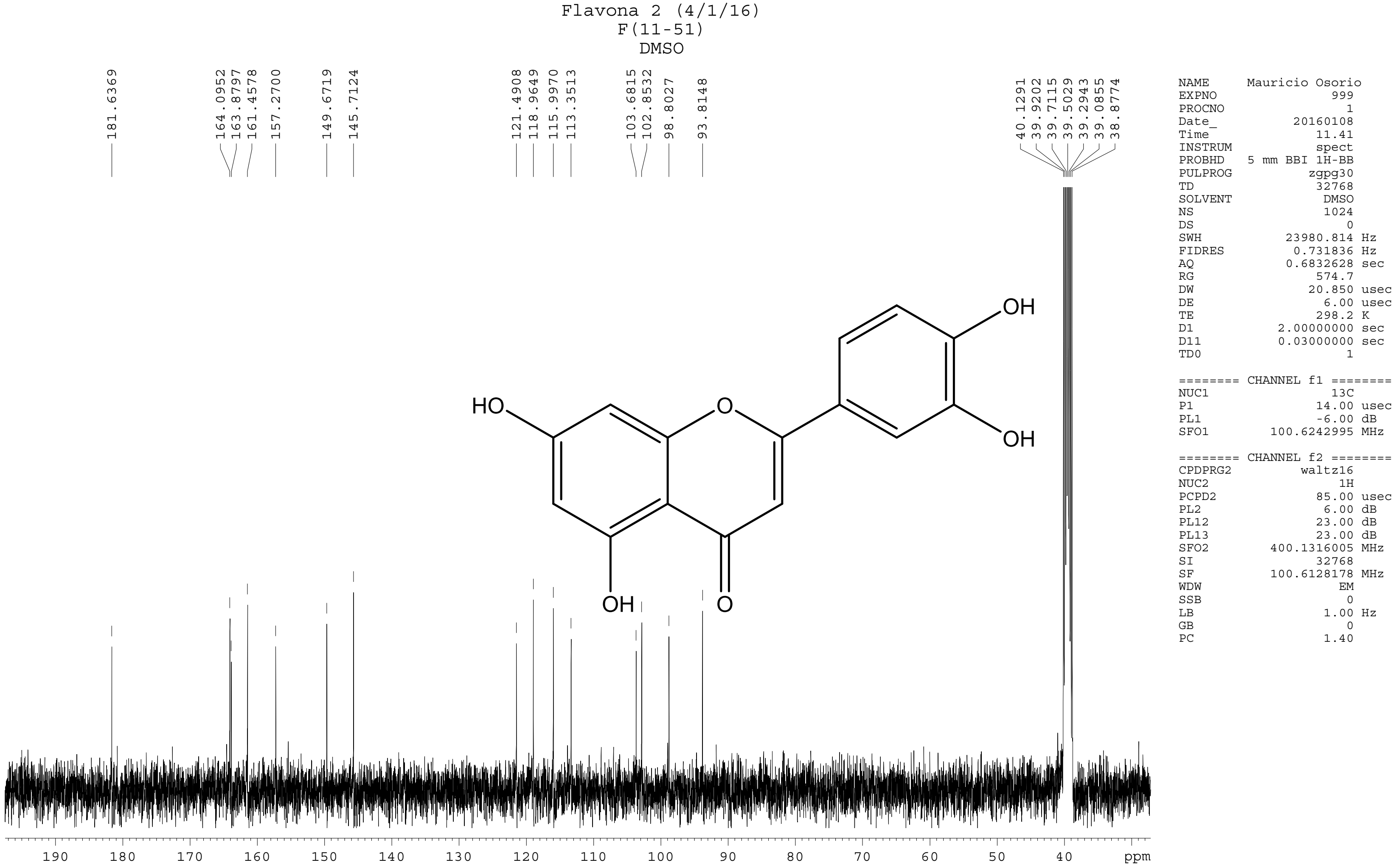

Figure S69. ^{13}C-NMR of **FO2** (DMSO-*d*$_6$).

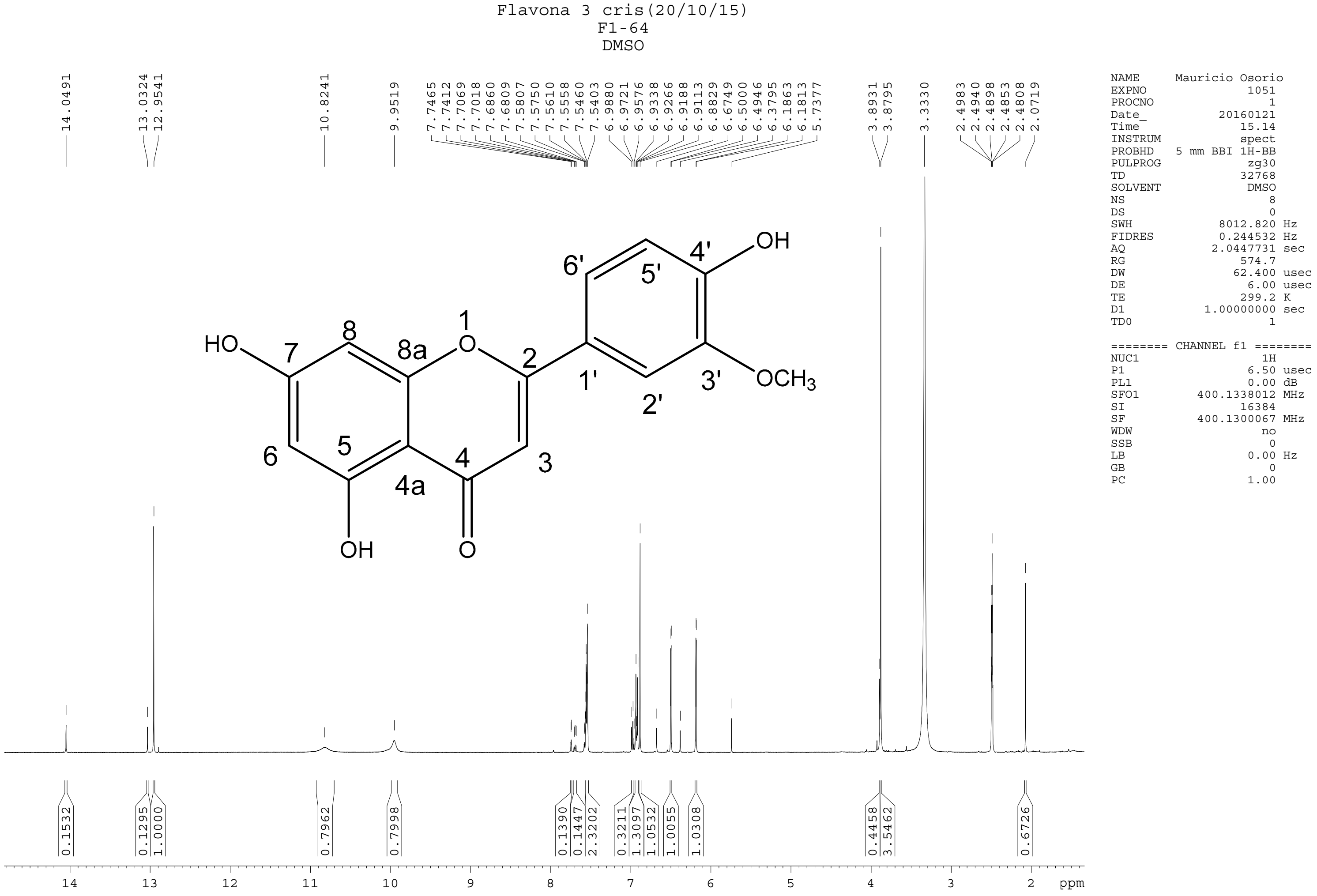

Figure S70. ^{1}H-NMR of **FO3** (DMSO-d_6).

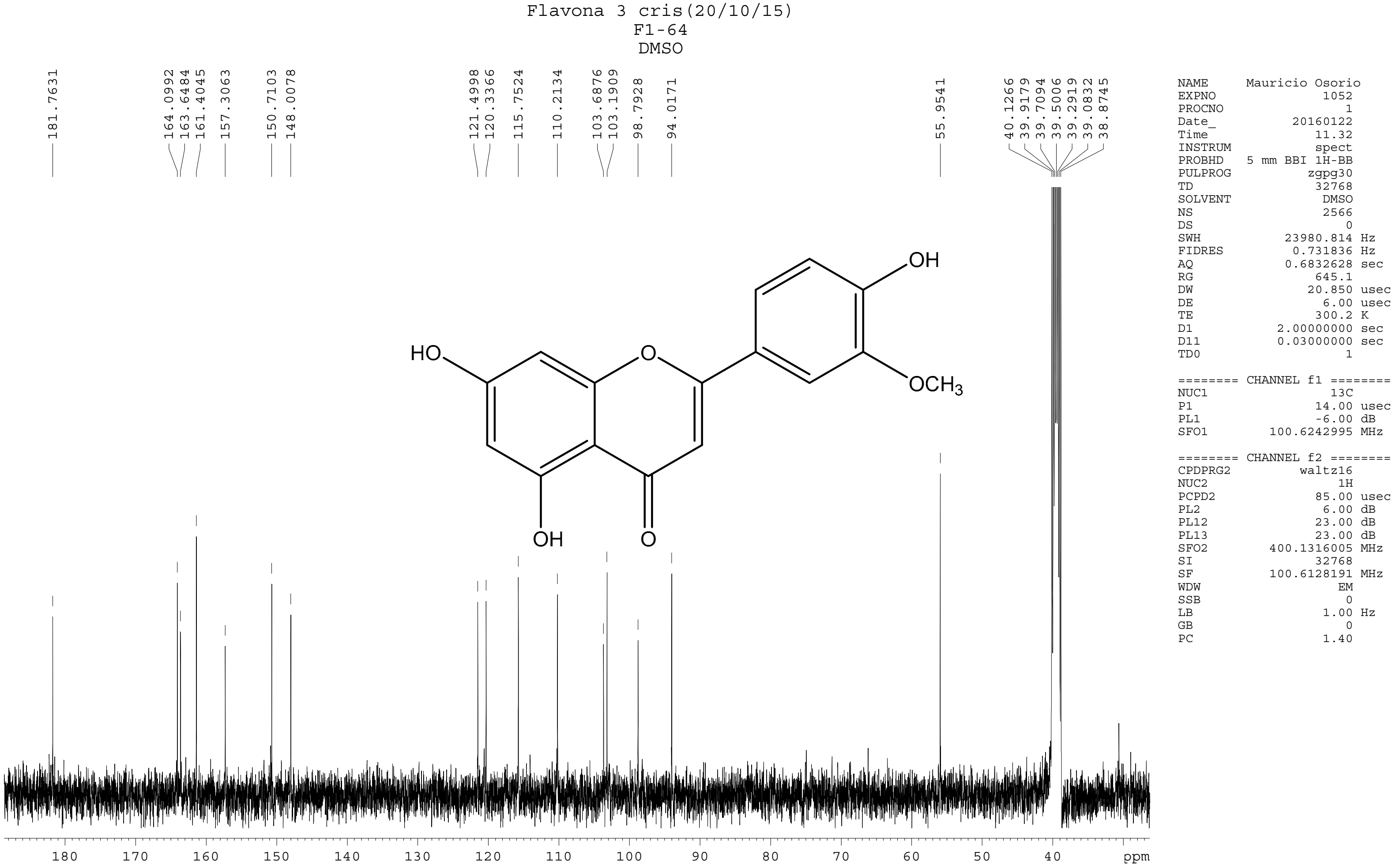

Figure S71. ^{13}C-NMR of **FO3** (DMSO-d_6).

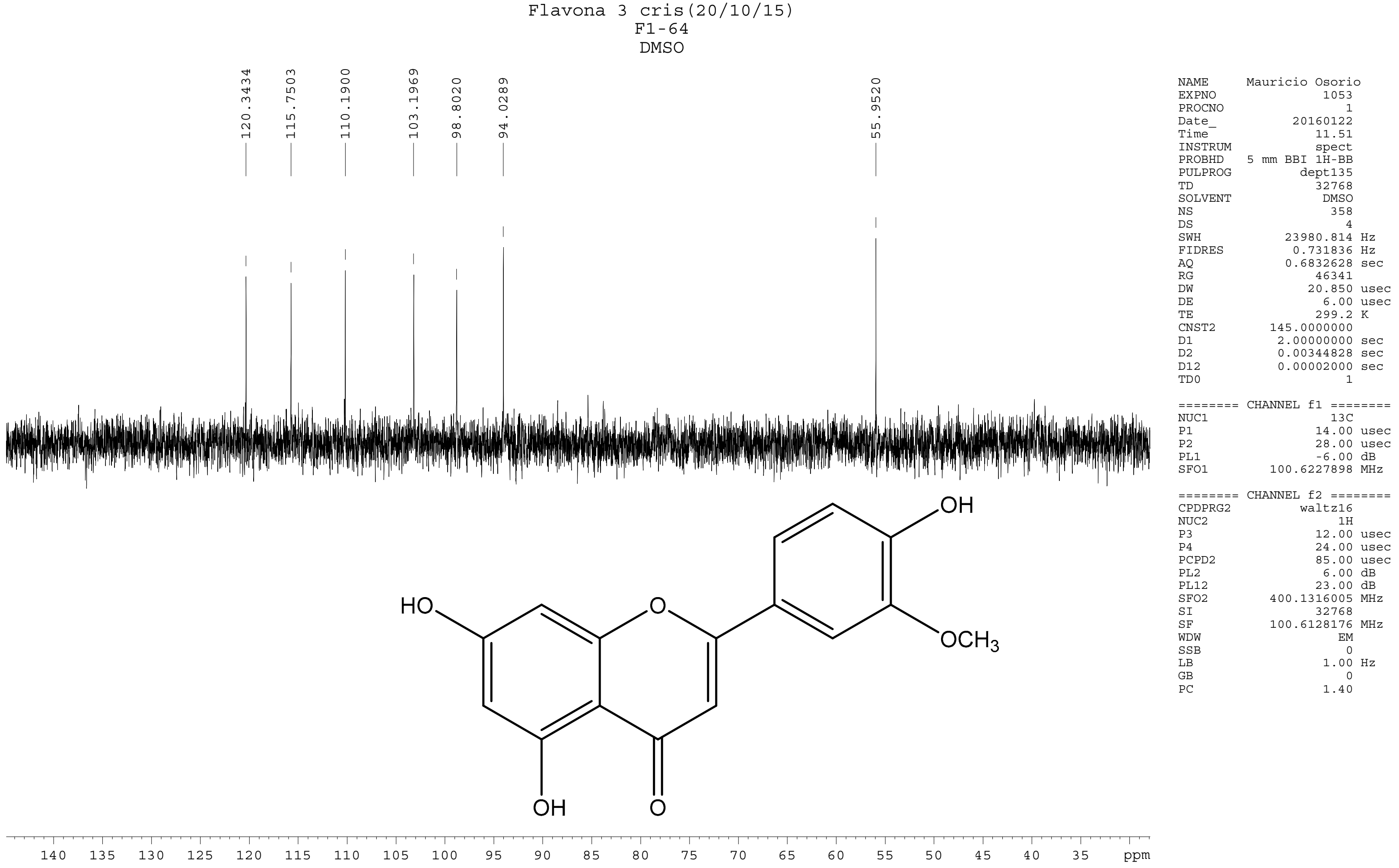

Figure S72. DEPT-135 of **FO3**.

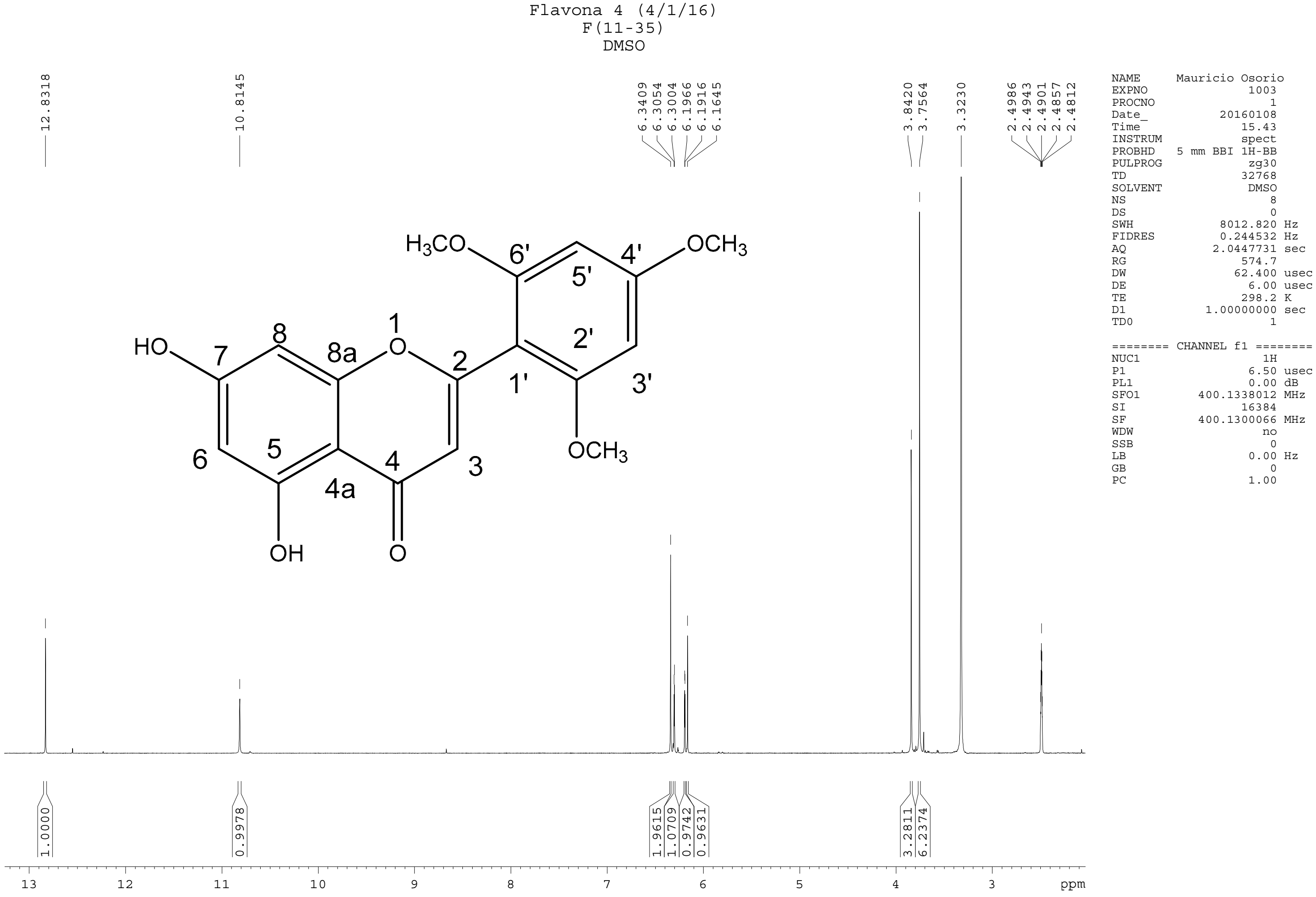

Figure S73. ^{1}H-NMR of **FO4** (DMSO-d_6).

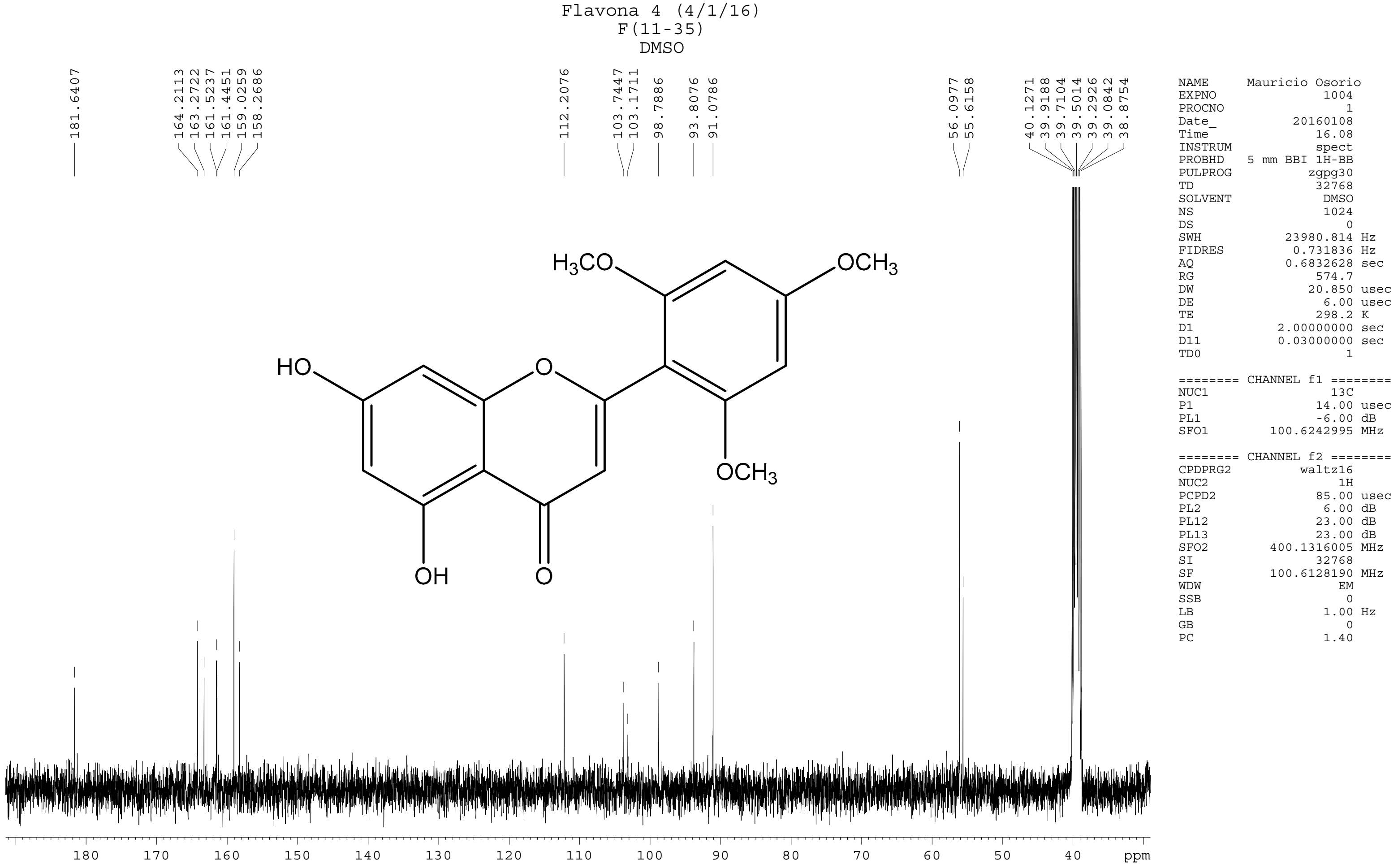

Figure S74. ^{13}C-NMR of **FO4** (DMSO-d_6).

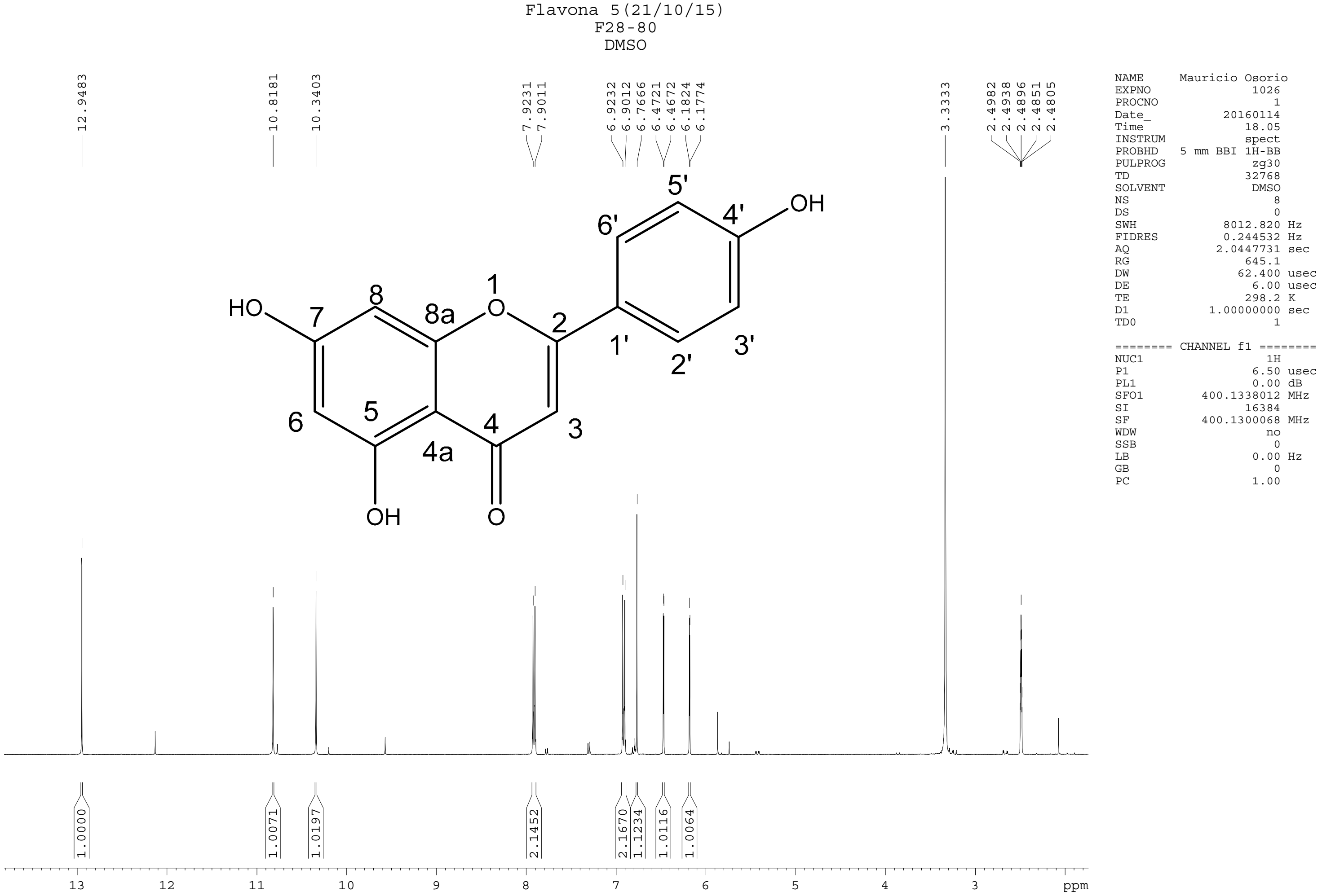

Figure S75. ^{1}H-NMR of **FO5** (DMSO-d_6).

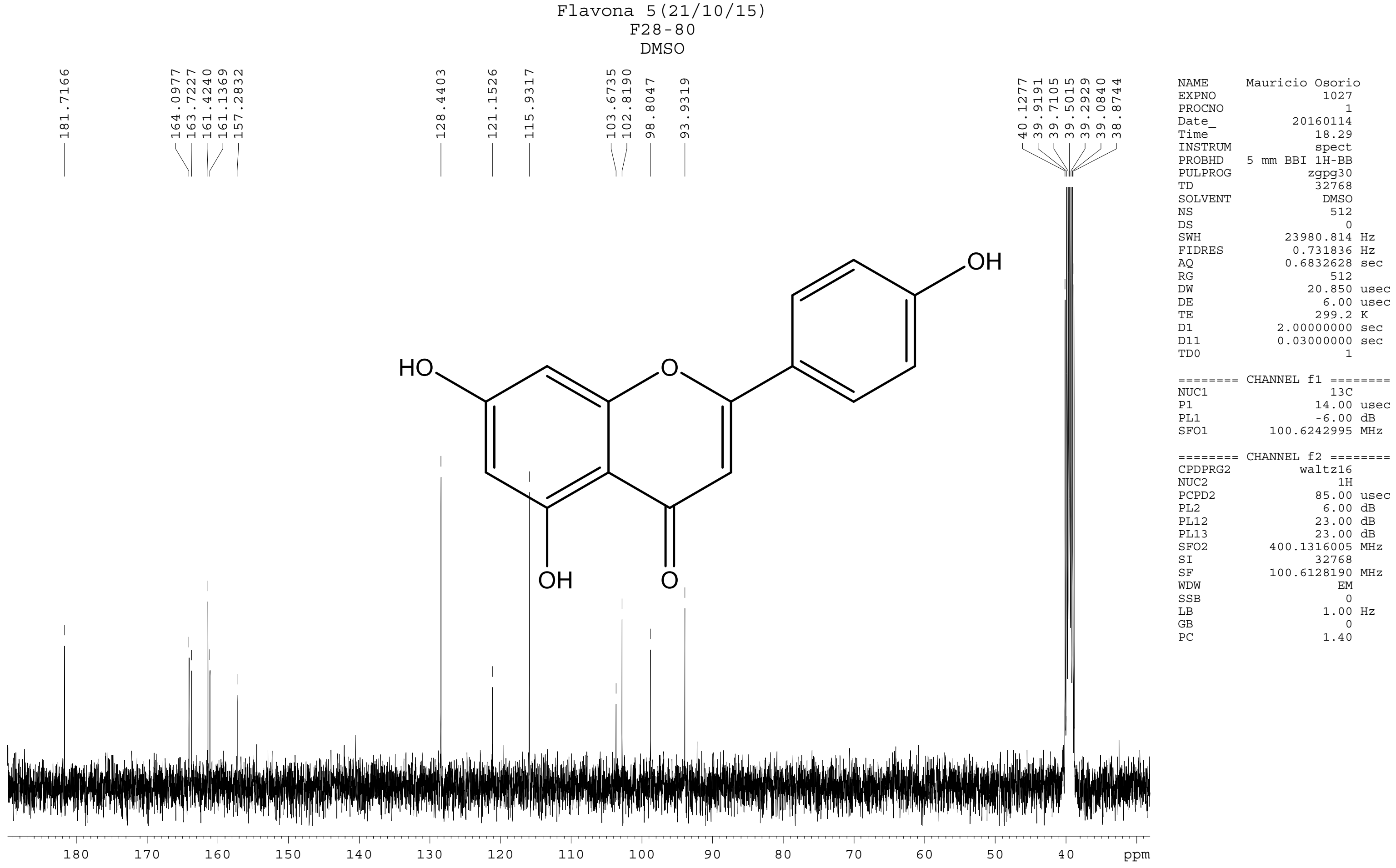

Figure S76. ^{13}C-NMR of **FO5** (DMSO-d_6).

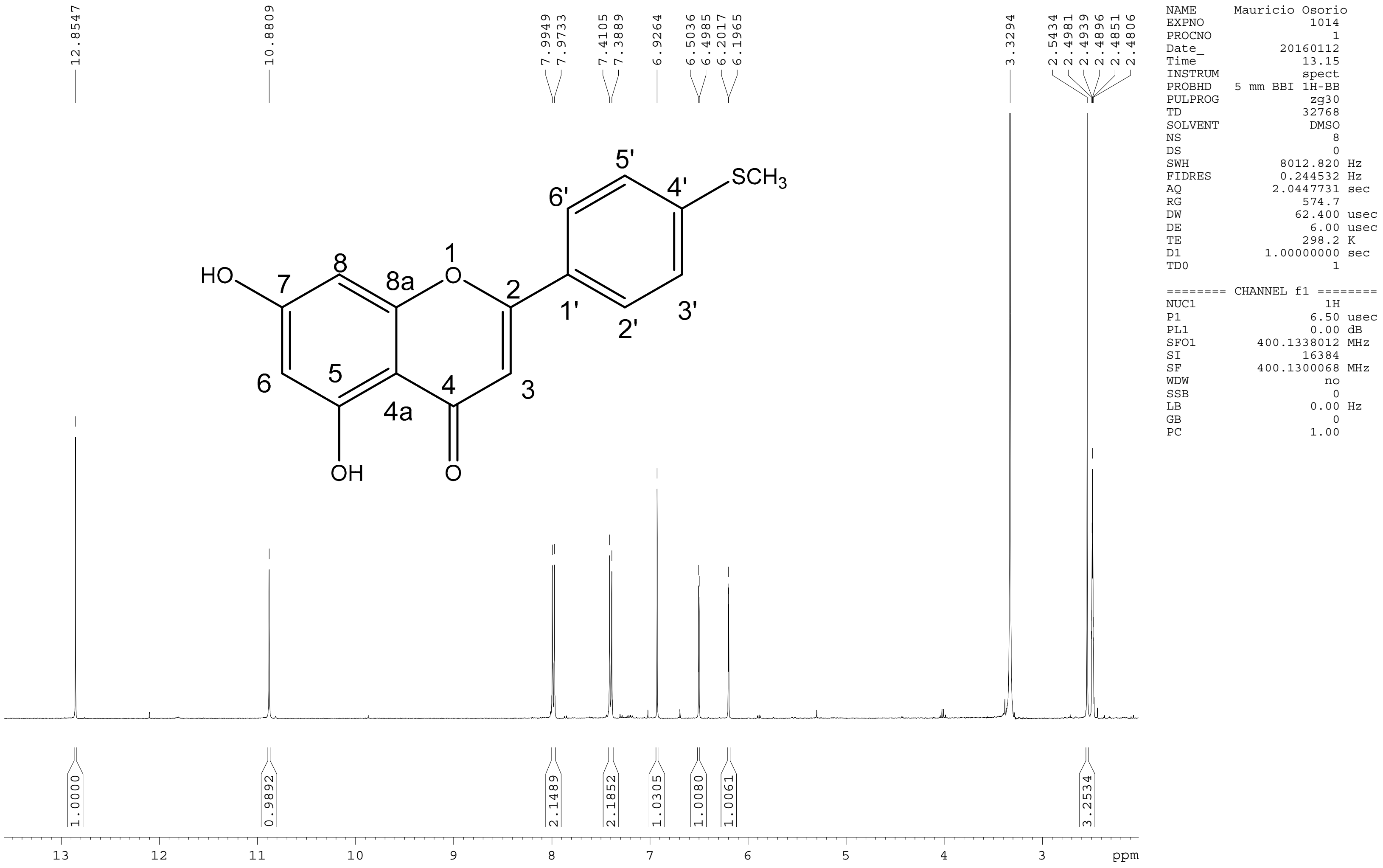

Figure S77. ^{1}H-NMR of **FO6** (DMSO-*d*$_6$).

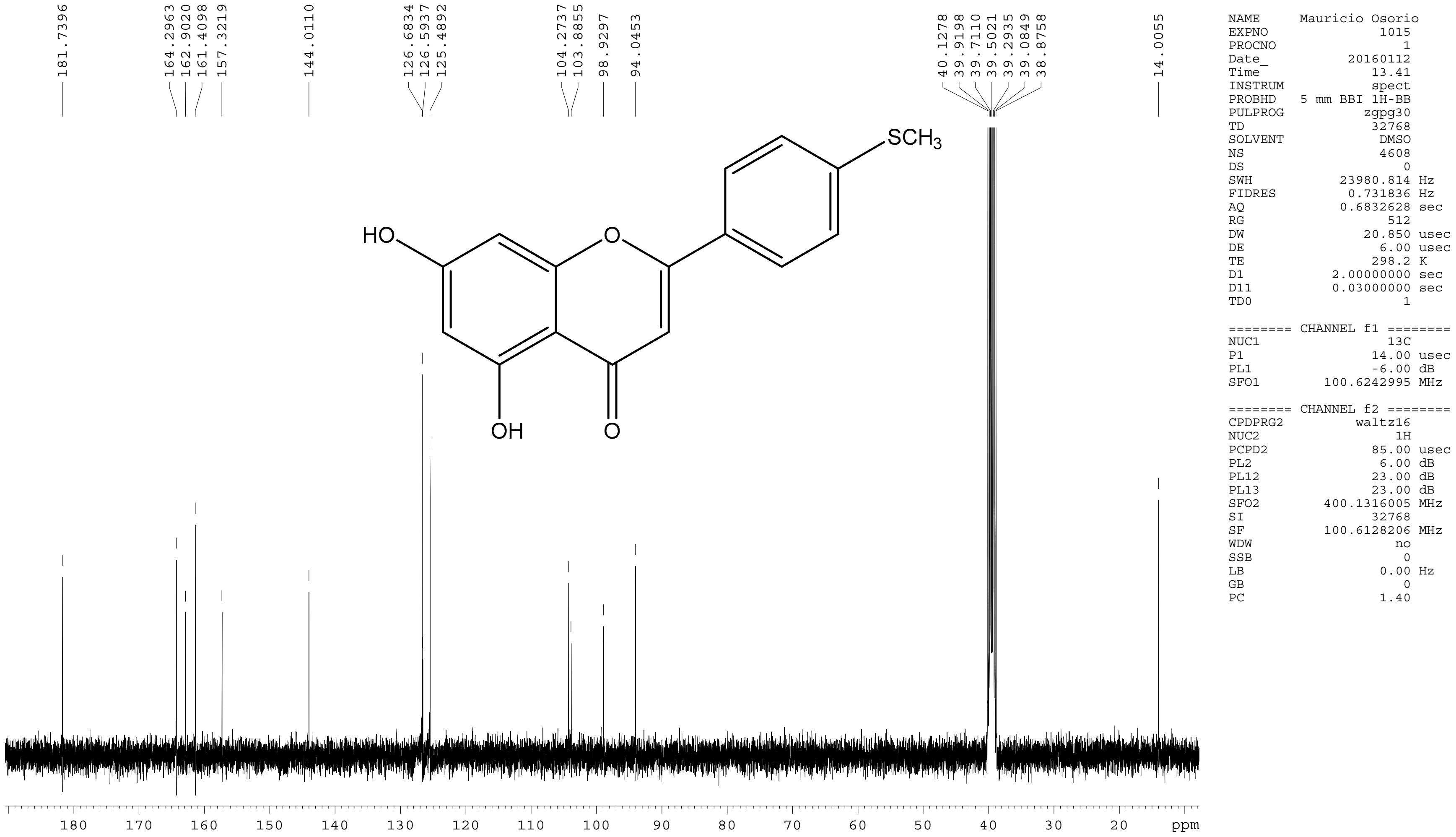

Figure S78. ^{13}C-NMR of **FO6** (DMSO-d_6).

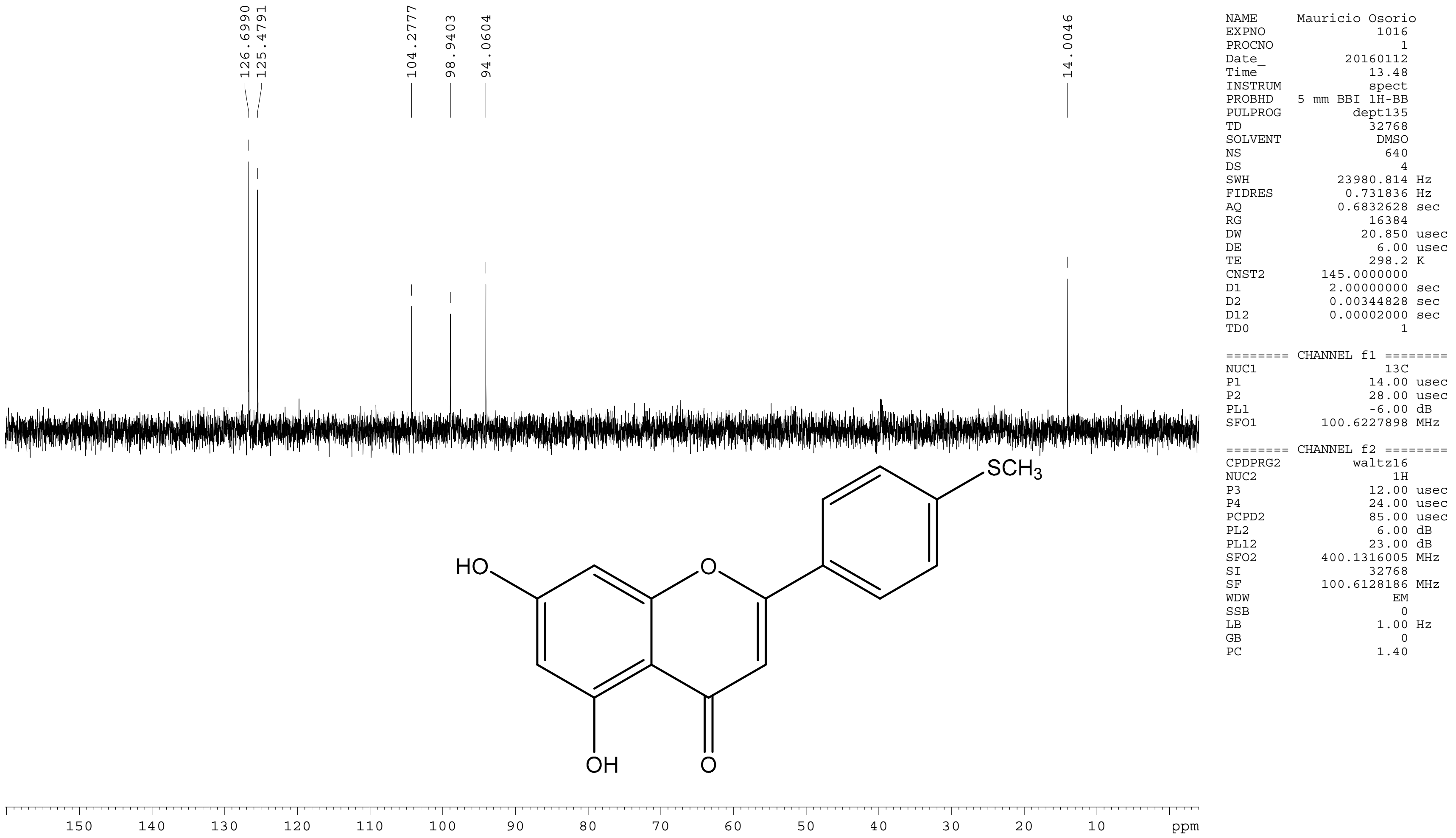

Figure S79. DEPT-135 of **FO6**.

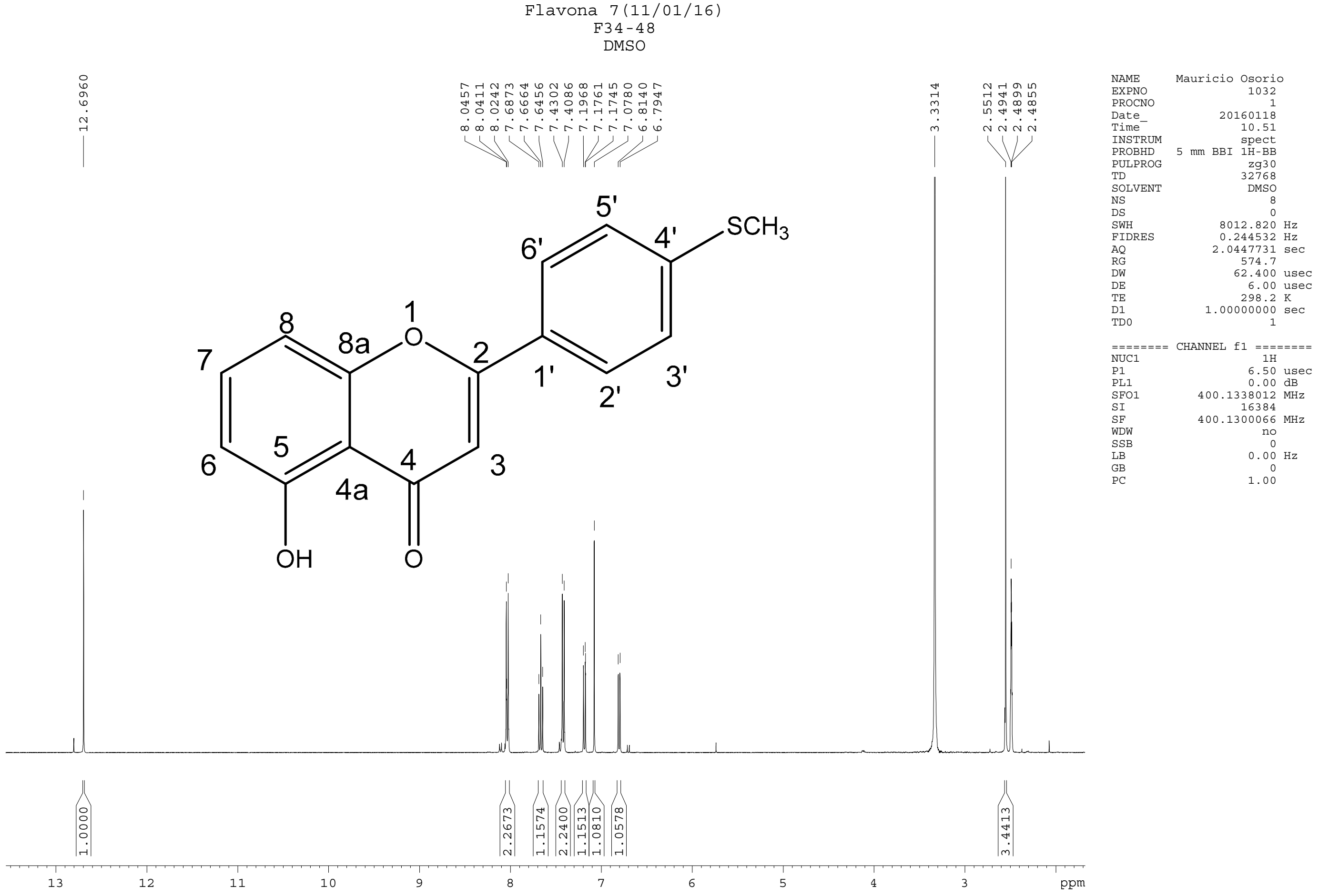

Figure S80. ^{1}H-NMR of **FO7** (DMSO-d_6).

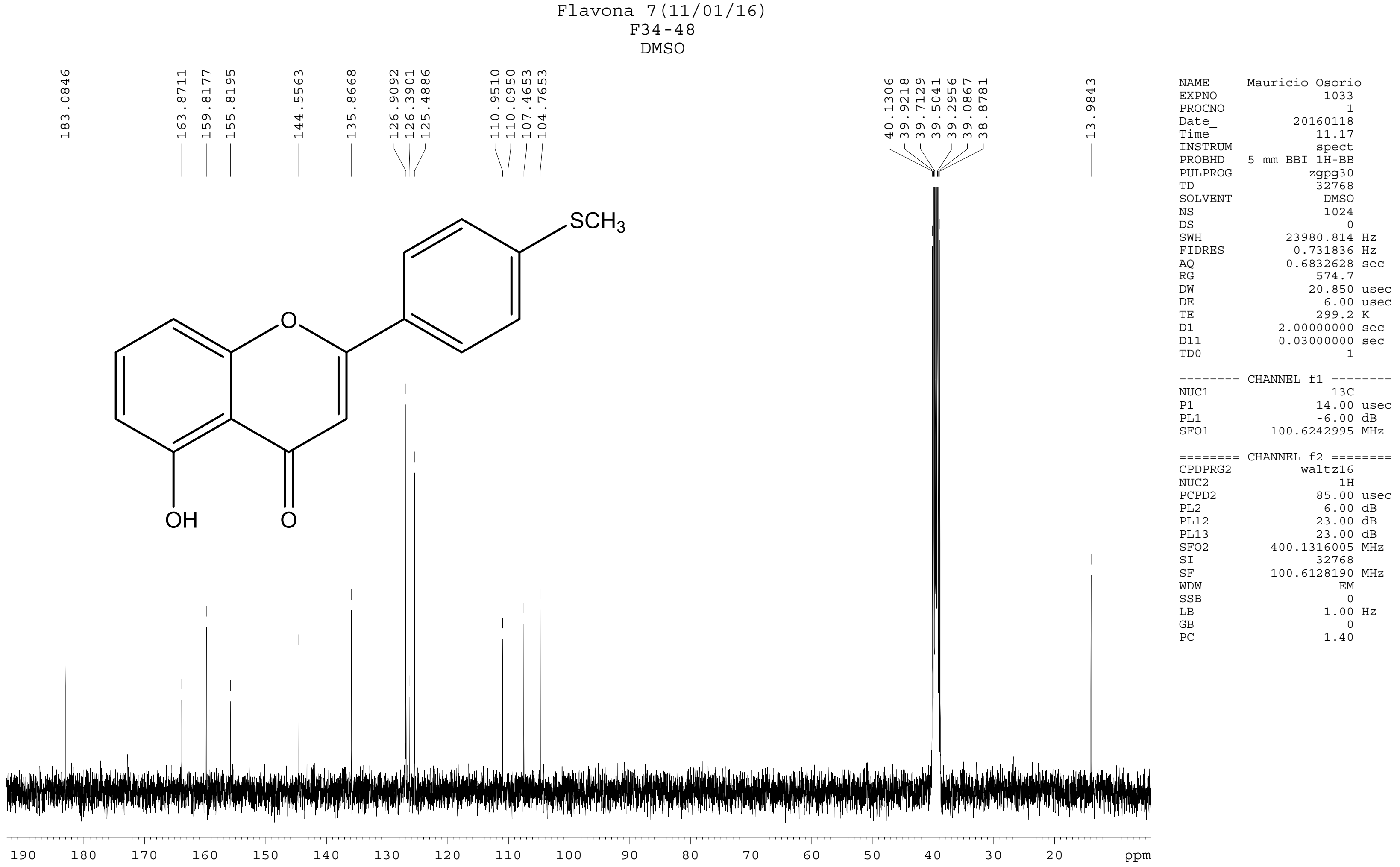

Figure S81. ^{13}C-NMR of **FO7** (DMSO-d_6).

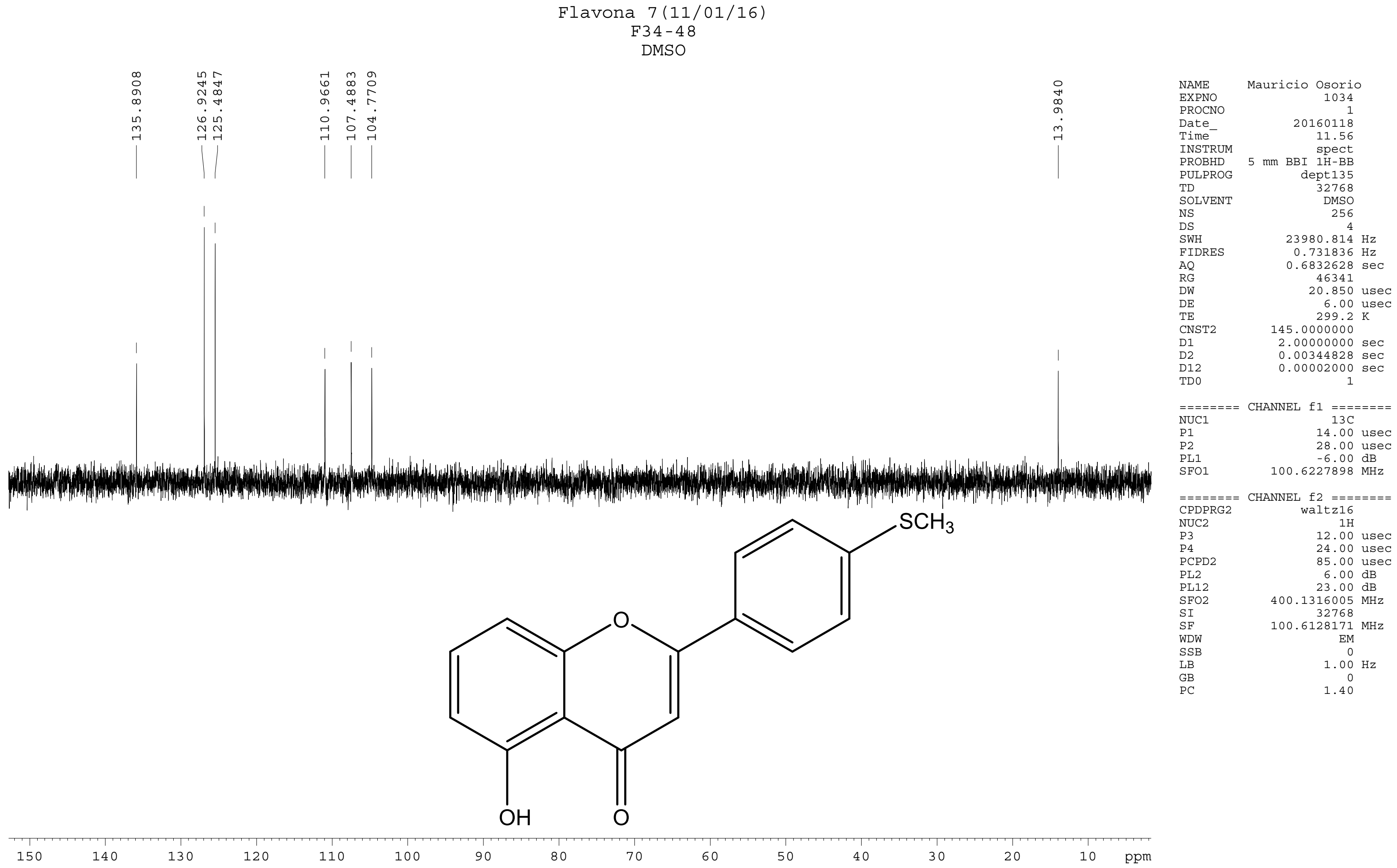

Figure S82. DEPT-135 of **FO7**.

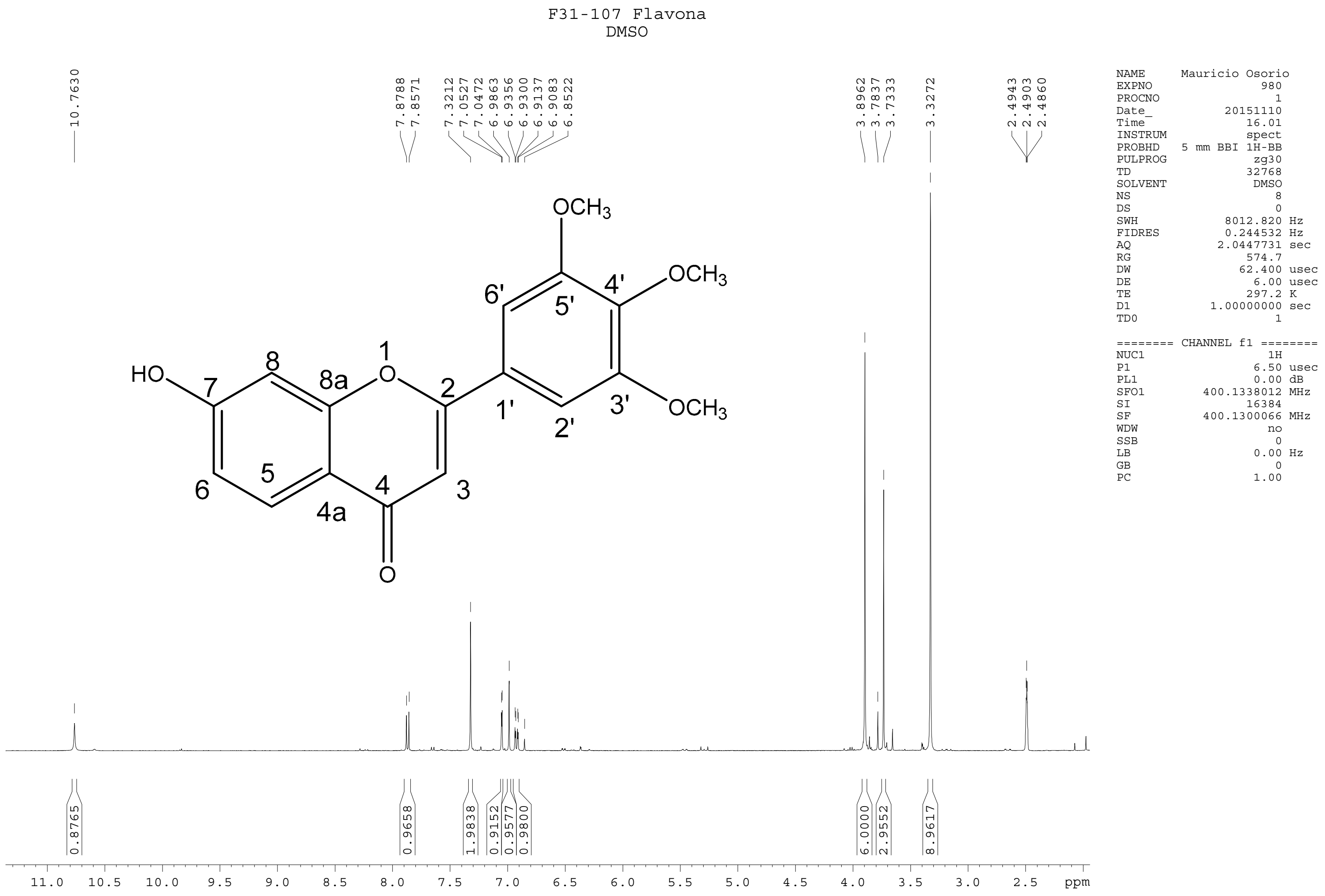

Figure S83. ^{1}H-NMR of **FO8** (DMSO-d_6).

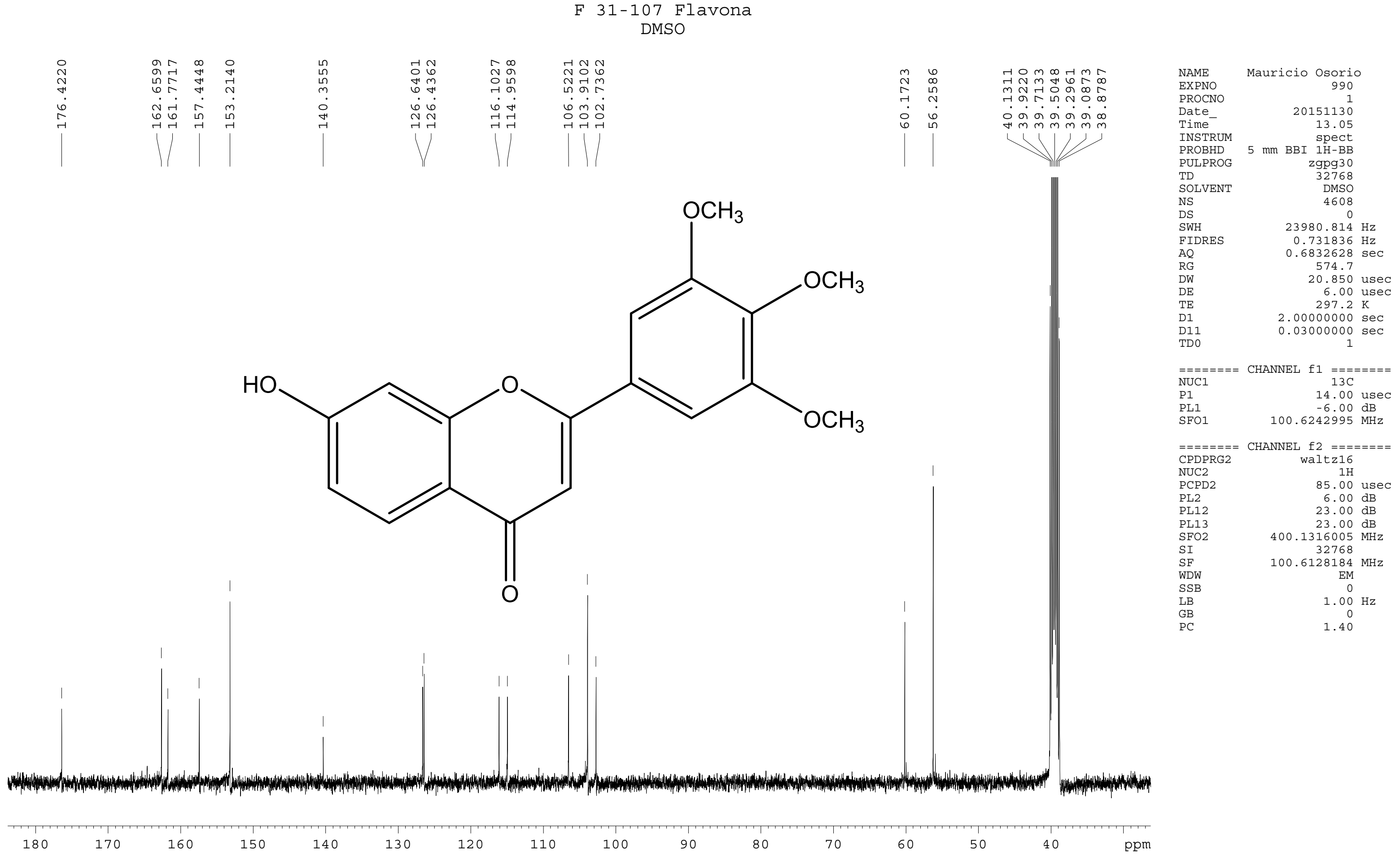

Figure S84. ^{13}C-NMR of **FO8** (DMSO-d_6).

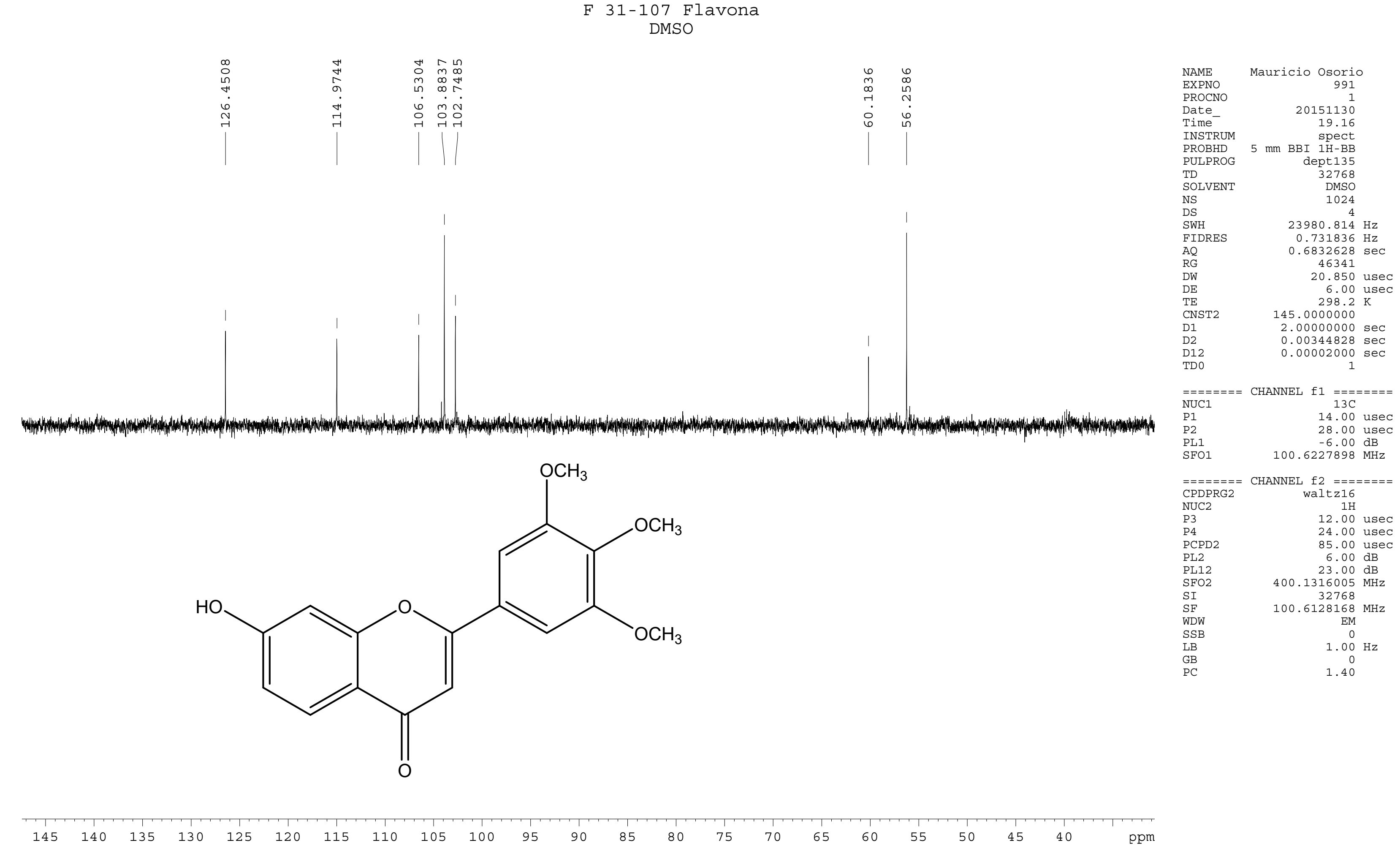

Figure S85. DEPT-135 of **FO8**.

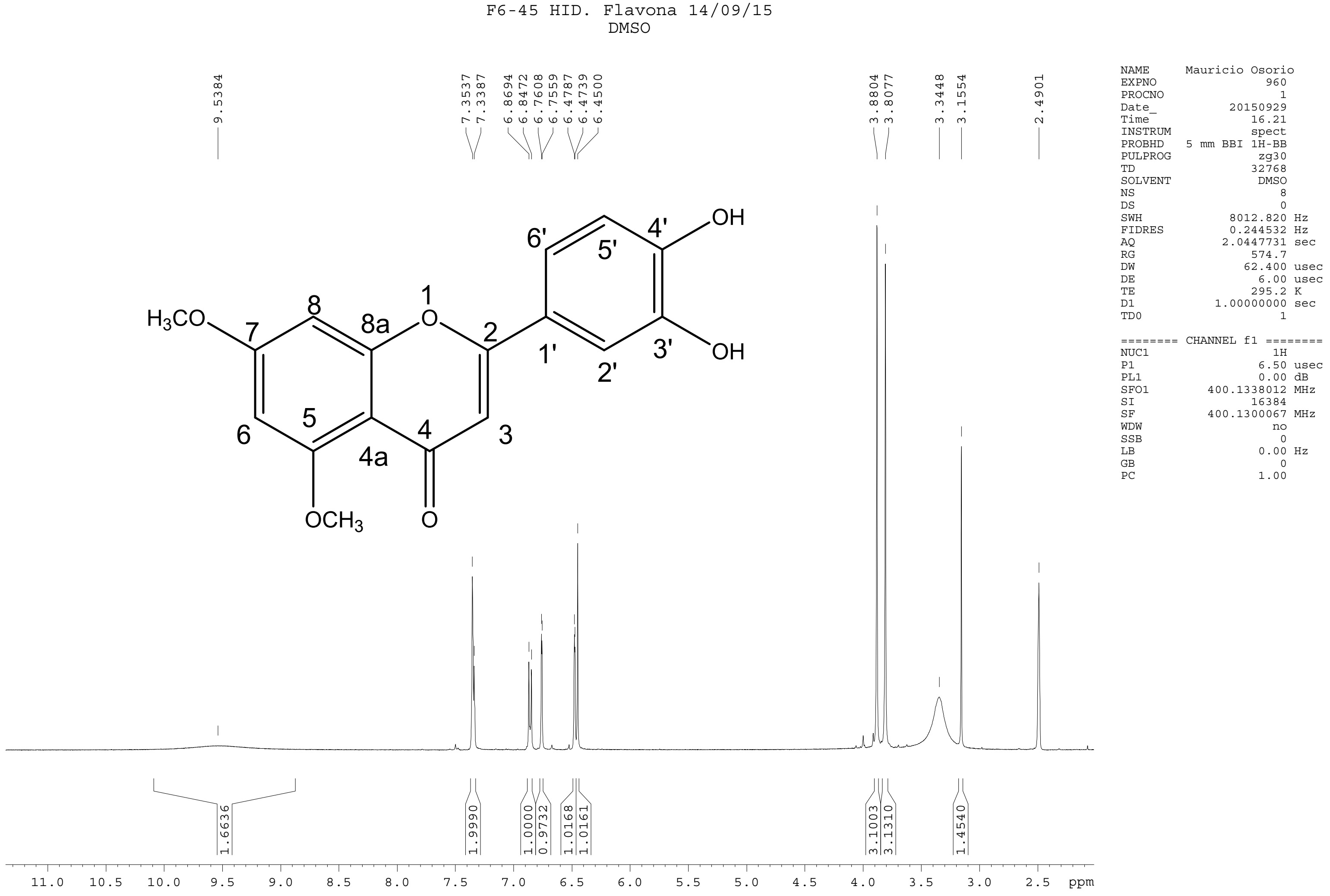

Figure S86. ^{13}C-NMR of **FO9** (DMSO-*d*$_6$).

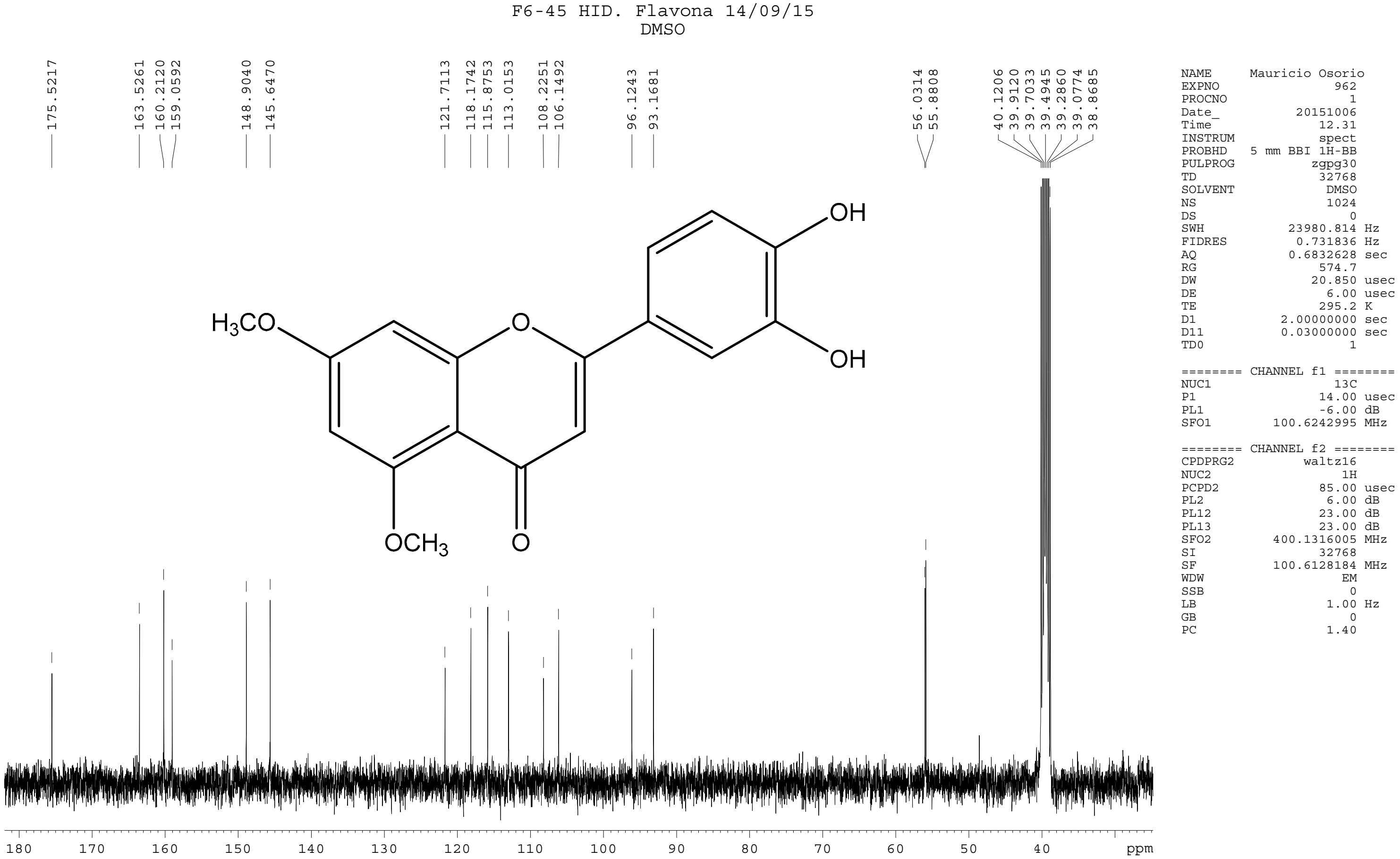

Figure S87. ^{13}C-NMR of **FO9** (DMSO-d_6).

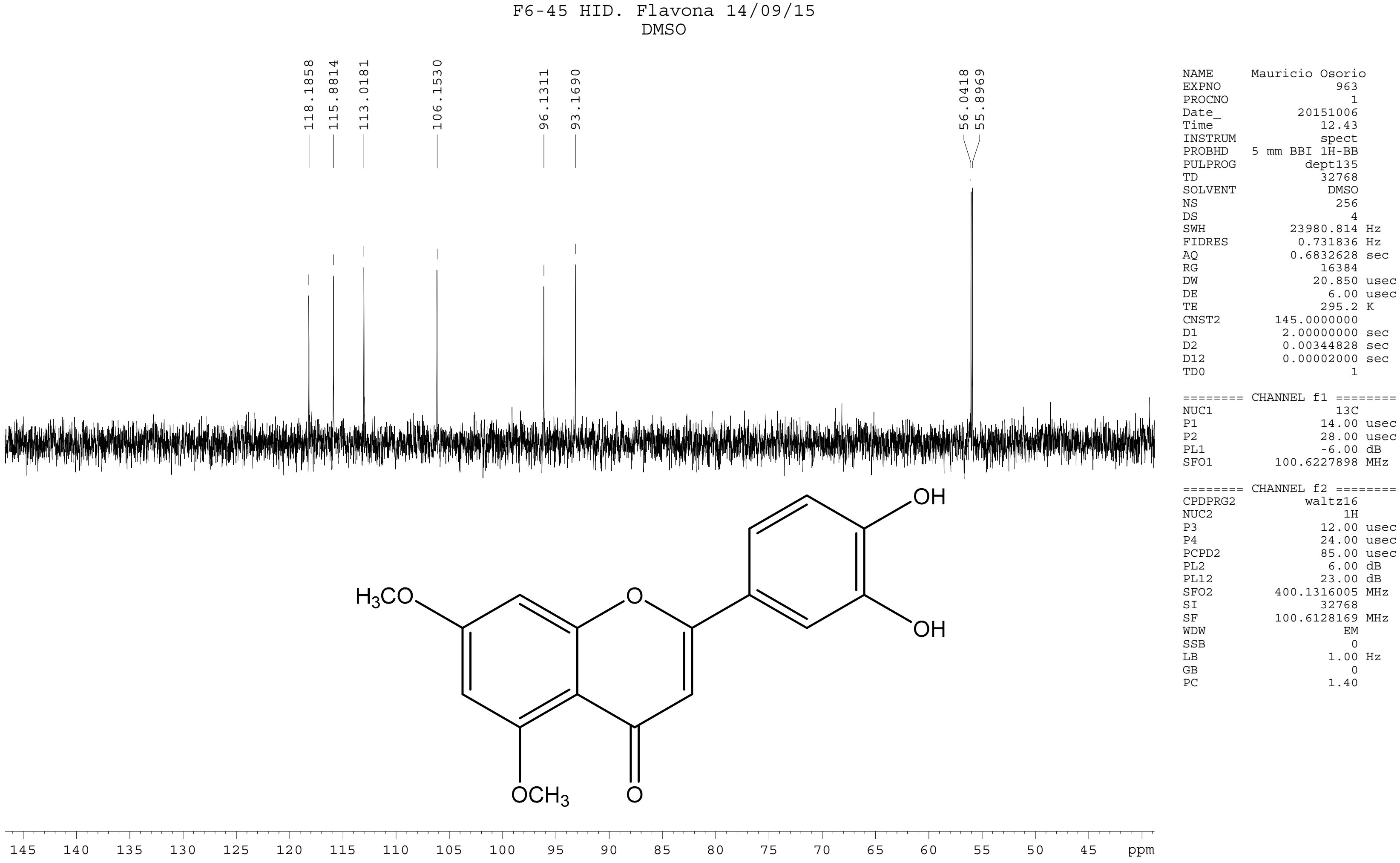

Figure S88. DEPT-135 of **FO9**.

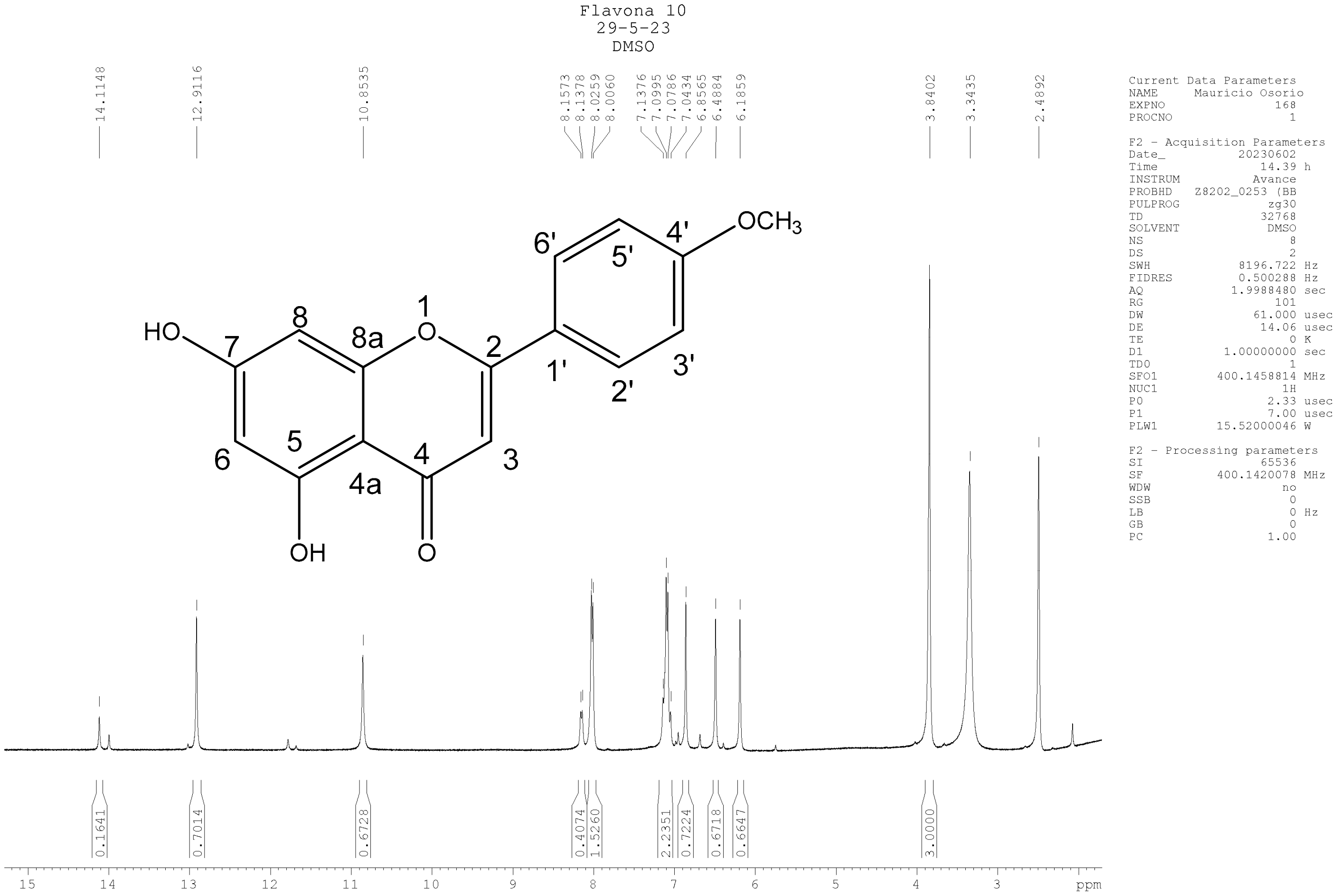

Figure S89. ^{1}H-NMR of **FO10** (DMSO-d_6).

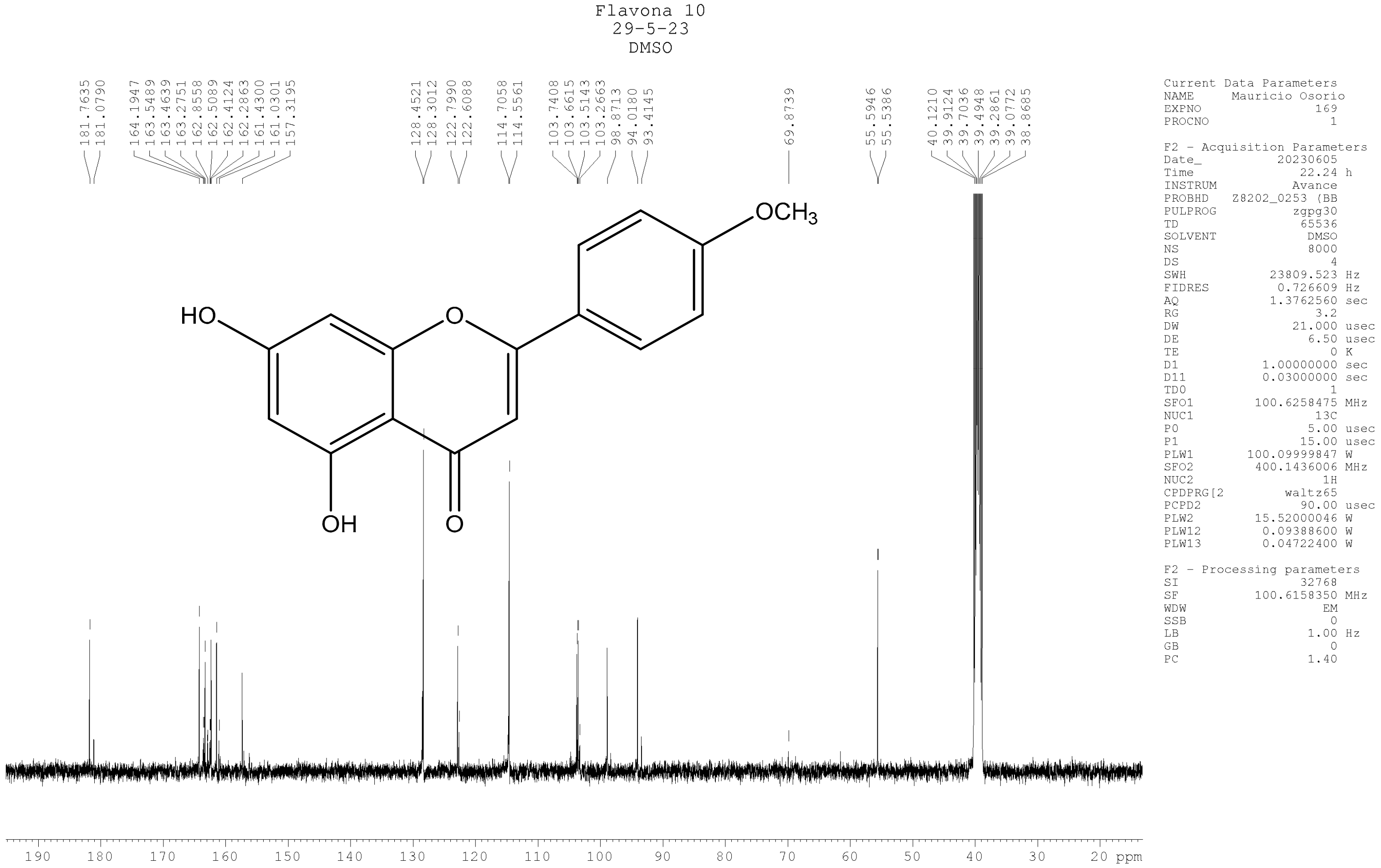

Figure S90. ^{13}C-NMR of **FO10** (DMSO-d_6).

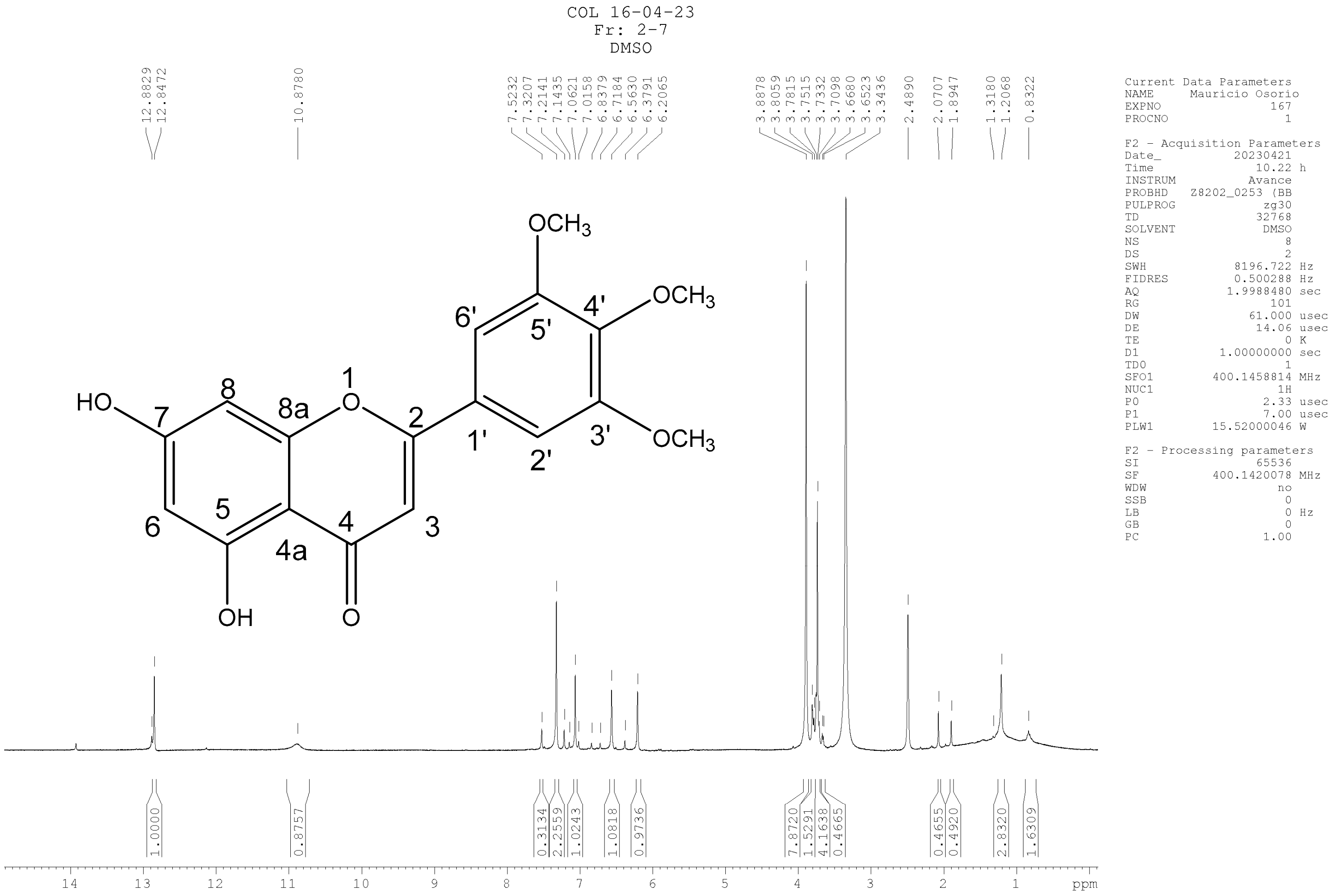

Figure S91. ^{1}H-NMR of **FO11** (DMSO-d_6).

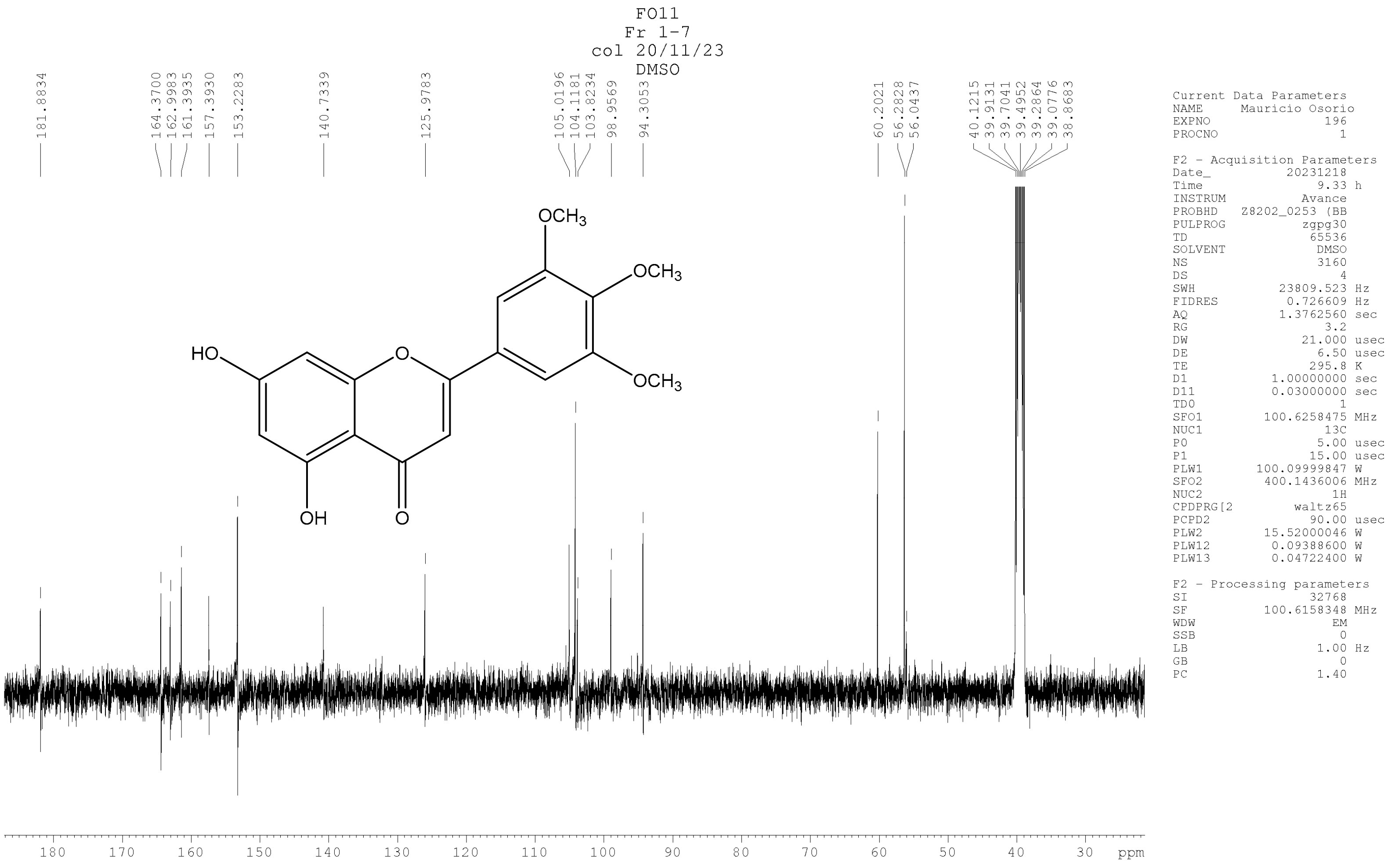

Figure S92. ^{13}C-NMR of **FO11** (DMSO-d_6).

High Resolution Mass Spectra of the new synthesized compounds.

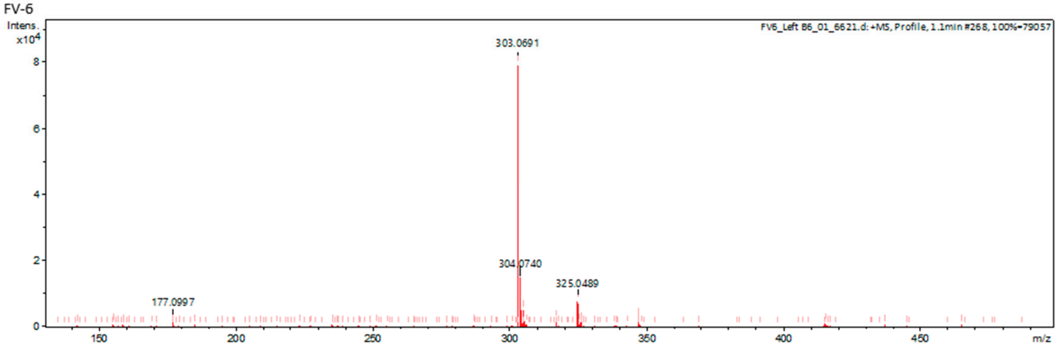

Figure S93. HRMS of **FV6**.

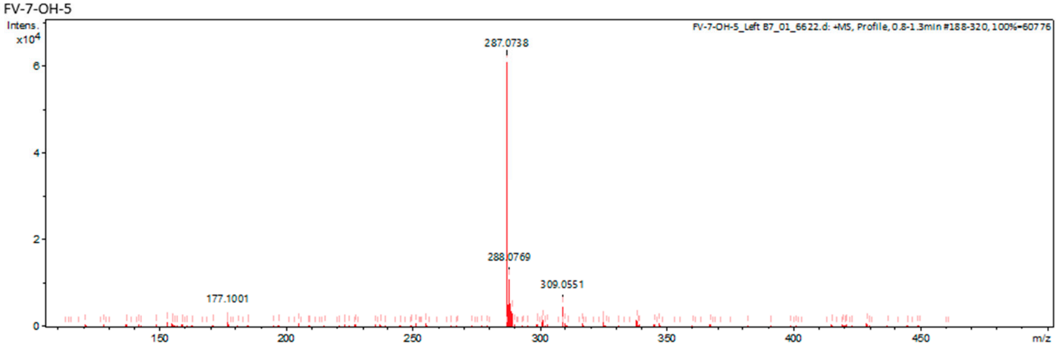

Figure S94. HRMS of **FV7**.

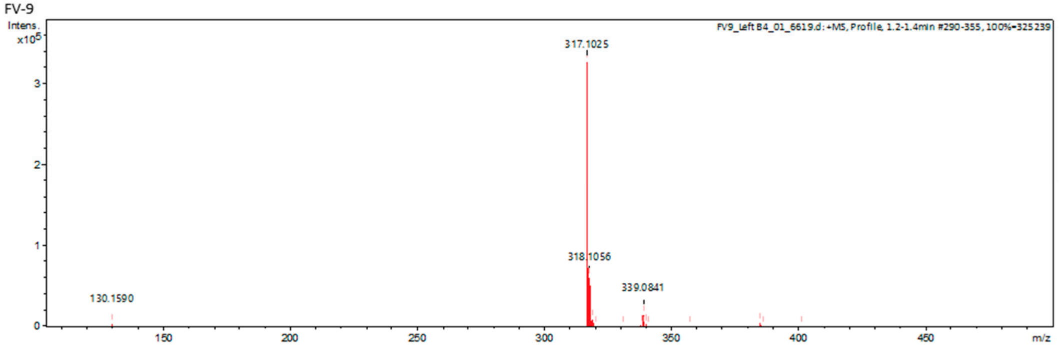

Figure S95. HRMS of **FV9**.

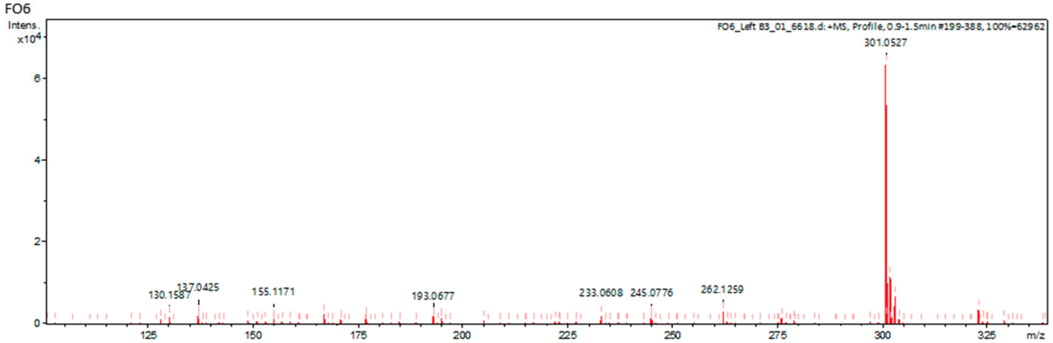

Figure S96. HRMS of **FO6**.

FO7

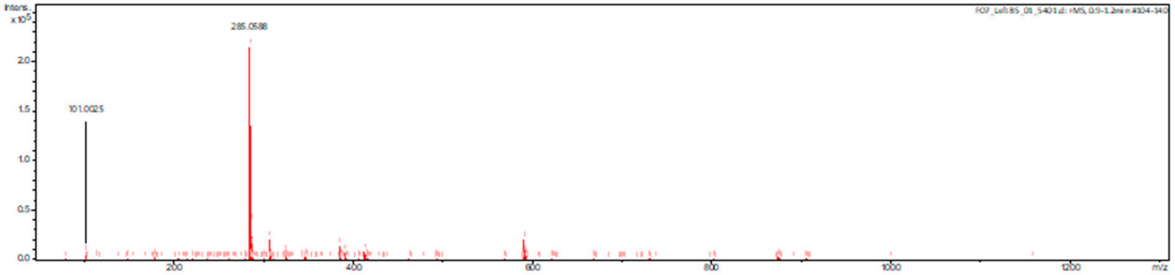

Figure S97. HRMS of **FO7**.
